# Integrated synthesis of 3,4-carbazoquinone alkaloids *N*-Me-carbazoquinocin A, B and D–F†

**DOI:** 10.1039/d4ra05347h

**Published:** 2024-08-28

**Authors:** Basavaiah Bommanaboina, Debayan Roy, Beeraiah Baire

**Affiliations:** a Department of Chemistry, Indian Institute of Technology Madras Chennai-600036 Tamil Nadu India beeru@iitm.ac.in

## Abstract

Carbazole alkaloids carbazoquinocin A–F possessing a 1-alkyl-2-methyl-3,4-*ortho*-carabazoquinone framework were isolated from the microorganism *Streptomyces violaceus 2448-SVT2* in 1995. Furthermore, they were found to exhibit strong inhibitory activity against lipid peroxidation. Herein, we report the integrated synthesis of *N*-Me-analogues of 5 members of the carbazoquinocin family of natural products, namely, *N*-Me-carbazoquinocin A, B and D–F. We employed an acid-catalyzed, intramolecular benzannulation of indole-appended *Z*-enoate propargylic alcohols, which was developed earlier in our laboratory, for the construction of the required carbazole framework. All five natural products were obtained in an overall yield of 50–60%, starting from a commercially available indole.

## Abstract 2

A unified synthesis of *N*-Me-carbazoquinocin A, B and D–F is reported. An acid-catalyzed intramolecular benzannulation of indole-appended *Z*-enoate propargylic alcohols, is employed for the construction of the carbazole framework.

## Graphical abstract

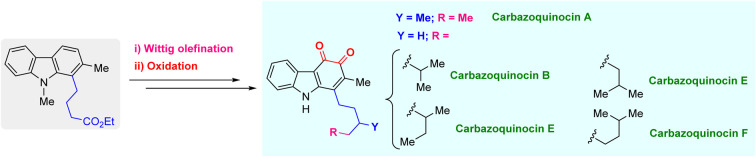

## Introduction

**Fig. 1:**
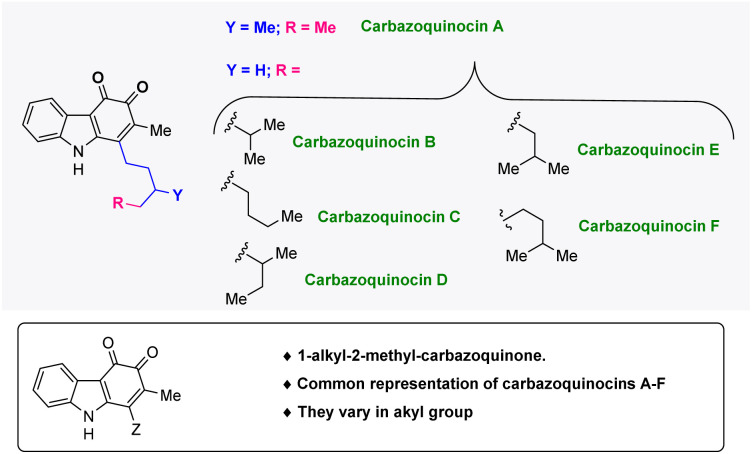
Structures of carbazoquinocin A–F natural products.

Carbazoles (dibenzopyrroles) are nitrogen-based tricyclic frameworks, which are essential in medicinal chemistry^1^ and materials chemistry.^2^ They are also part of many structurally complex bioactive natural products and drug molecules.^3^ Among the many structurally diverse carbazole natural products, a 1-alkyl-2-methyl-carbazole framework has attracted special attention as it has been found to be present in different families of bioactive carbazole natural products such as lipocarbazoles, carbazoquinocins, and carazostatin.^4^ In 1995, the research group of Seto isolated six carbazoquinocins A–F, from the microorganism *Streptomyces violaceus 2448-SVT2* (Fig. 1).^5^ These natural products share a common 1-alkyl-2-methyl-3,4-*ortho*-carbazoquinone framework and have been found to exhibit strong inhibitory activity against lipid peroxidation.^6^

Several research groups have reported the total synthesis of some of these novel alkaloids.^7^ For example, the total synthesis of carbazoquinocins A and D was achieved by the research group of Ogasawara in 1996.^7*a*^ Alternatively, Hibino *et al.* reported the total synthesis of carbazoquinocins B–F.^7*b*^ In 2003, Wulff and coworkers reported the total synthesis of carbazoquinocin C.^7*c*^

**Scheme 1:**
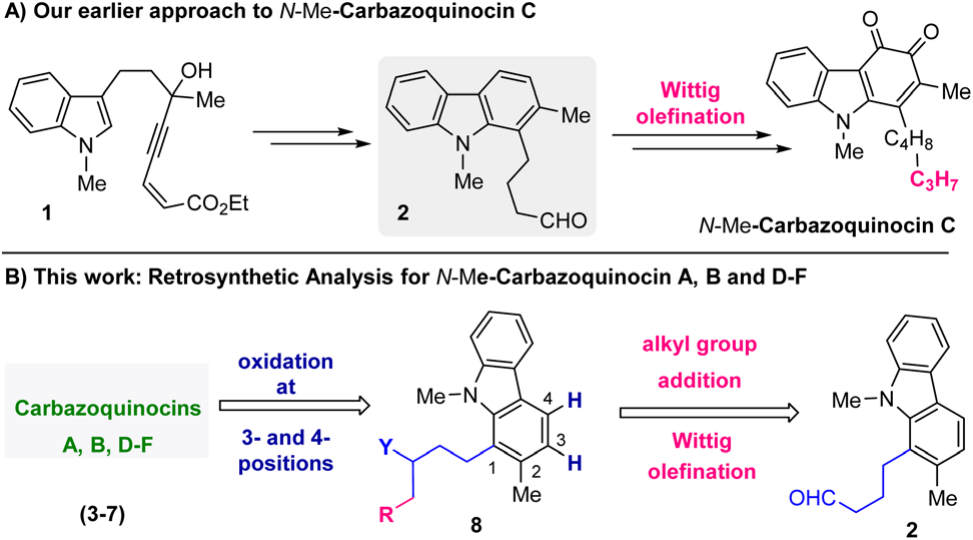
(A) Our earlier work: total synthesis of *N*-Me-carbazoquinocin C. (B) Retrosynthetic analysis for the total synthesis of carbazoquinocins A, B, and D–F (3–7) from aldehyde 2.

Recently, in 2022, we developed an approach for the total synthesis of the 1-alkyl-2-methyl-3,4-carbazoquinone natural product *N*-Me-carbazoquinocin C together with *N*-Me-carazostatin and *N*-Me-lipocarbazole A4.^7*d*^ This approach employs a Brønsted acid-catalyzed intramolecular benzannulation of C-3-tethered indole-propargylic alcohols (1) (Scheme 1A) as the key step for the construction of a carbazole unit (2). In continuation, we envisioned an opportunity to extend this methodology for the total synthesis of other members of the carbazoquinocin family, *i.e.*, carbazoquinocin A, B, and D–F (3–7) (Fig. 1). According to our retrosynthetic analysis (Scheme 1B), butyraldehyde present in 2 can be converted into the respective alkyl chain (as in 8) required for carbazoquinocin A, B and D–F through the Wittig olefination reaction using suitable phosphorous ylides. Further, oxidation at the C-3 and C-4 positions of the resultant carbazoles 8 to generate the natural products carbazoquinocin A, B and D–F (3–7) can be achieved through C-3-methoxylation, followed by selenium promoted oxidations.

## Results and discussion

**Scheme 2:**
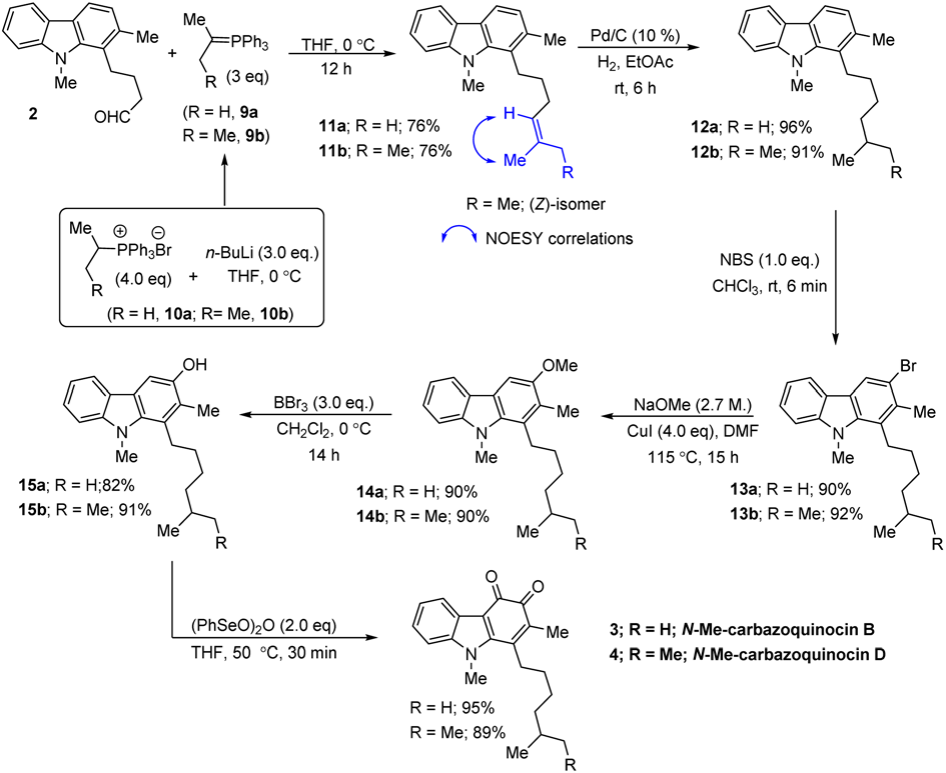
Total synthesis of *N*-Me-carbazoquinocin B and D.

Our approach began with the synthesis of the required carbazole–butyraldehyde (2) from *N*-Me-indole, following our earlier procedure.^7*d*^ Initially, we aimed at the total synthesis of *N*-Me-carbazoquinocin B (3) (Scheme 2, R = H). Accordingly, Wittig olefination of aldehyde 2 with phosphorous ylide (9a) at 0 °C (generated *in situ* from (iso-propyl)PPh_3_Br^8^ (10a) and ^*n*^BuLi at 0 °C) afforded olefin (11a) in 76% yield. The hydrogenation reaction of 11a with Pd/C (10%) under an H_2_ atmosphere in ethyl acetate at room temperature (rt) for 6 h gave 1-isoheptylcarbazole (12a) (96% yield).

**Scheme 3:**
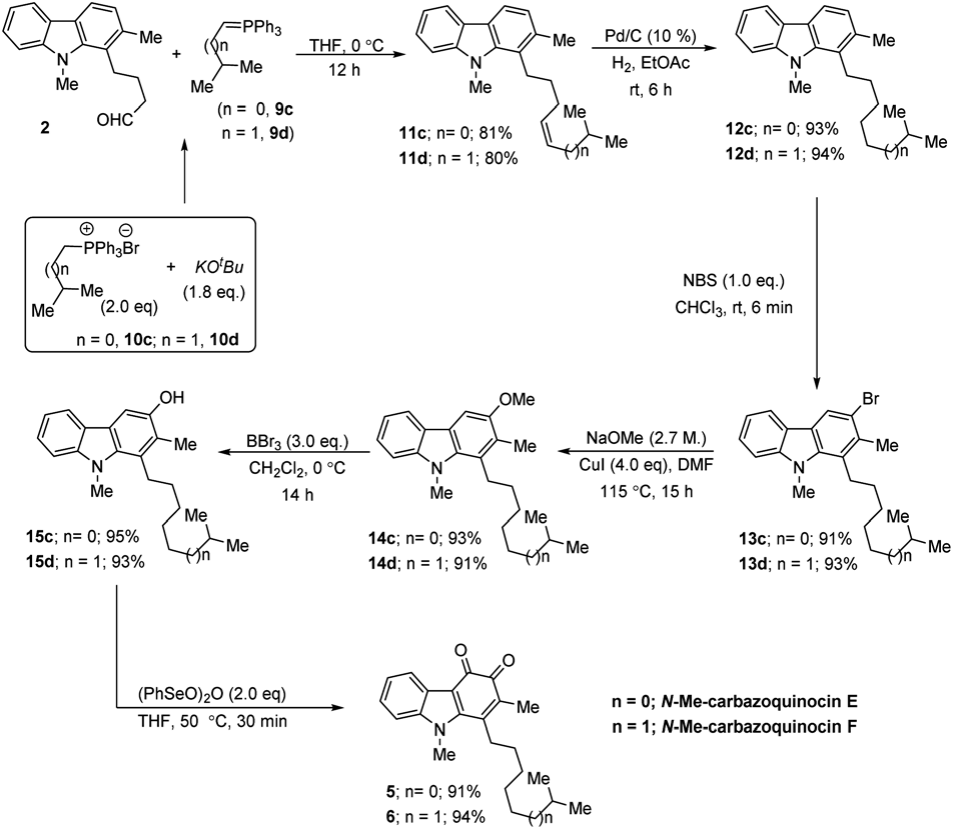
Unified total synthesis of *N*-Me-carbazoquinocin E and F.

Next, the regioselective electrophilic bromination of carbazole 12a with NBS in CHCl_3_ at rt gave the corresponding 3-bromocarbazole (13a) (90%) within 6 min reaction time. Next, we employed a two-step strategy for the efficient conversion of bromine in 13a into a hydroxyl group. Initially, heating a DMF suspension of 13a, NaOMe and CuI at 115 °C gave 3-methoxy carbazole (14a) in 90% yield. Subsequent treatment of 14a with BBr_3_ in CH_2_Cl_2_ at 0 °C to rt for 14 h resulted in the formation of the phenol product (15a) in 82% yield *via* deprotection of the methoxy group. Finally, the synthesis of *N*-Me-carbazoquinocin B 3 was achieved by employing the (PhSeO)_2_O-promoted oxidation of 15a. Accordingly, heating (50 °C) a solution of 15a and (PhSeO)_2_O in THF gave *N*-Me-carbazoquinocin B 3 in 95% yield. After successfully completing the total synthesis of *N*-Me-carbazoquinocin B 3, we extended this strategy for the synthesis of *N*-Me-carbazoquinocin D 4 by employing the carbazole–butyraldehyde 2 as the starting point (Scheme 2, R = Me). According to our retrosynthetic analysis (Scheme 1B), the only difference between carbazoquinocin B 3 and carbazoquinocin D 4 is the incorporation of an iso-propyl group (R = H) *vs. sec*-butyl group (R = Me) in aldehyde 2. Therefore, olefination of aldehyde 2 using *sec*-butylidene phosphorous ylide 9b (generated from (*sec*-butyl)PPh_3_Br^8^10b and ^*n*^BuLi at 0 °C) afforded *Z*-olefin 11b (R = Me) in 76% yield. The geometry of the olefin in 11b was identified by using the correlations observed between =CMe and =C–H in the corresponding NOESY NMR spectrum. Further, by operating the same sequence of five functional group transformations, as depicted in Scheme 2 (R = Me), *i.e.*, hydrogenation (12b, 91%)–bromination (13b, 92%)–methoxylation (14b, 90%)–demethylation (15b, 91%)–oxidation (4, 89%), we efficiently converted olefin 11b into *N*-Me-carbazoquinocin D 4, with an overall yield of 61% for five steps. Subsequently, we envisioned the extension of this methodology for the total synthesis of *N*-Me-carbazoquinocin E 5 and F 6 (Scheme 3). The required alkyl chains at the C_1_-position present in compounds 5 and 6 could be achieved by incorporating an iso-butyl group (*n* = 0) and iso-pentyl group (*n* = 1) in butyraldehyde 2*via* Wittig olefination reaction, respectively. Wittig olefination of aldehyde 2 using iso-butylidene phosphorous ylide 9c (ref. 8) and iso-pentylidene phosphorous ylide 9d (ref. 8) exclusively afforded *Z*-olefins 11c (*n* = 0; 81%) and 11d (*n* = 1; 80%), respectively. After having both olefins 11c and 11d in hand, we employed the five-step synthetic strategy (as shown for *N*-Me-carbazoquinocin B 3 and D 4 in Scheme 2), *i.e.*, hydrogenation (12c & 12d)–bromination (13c & 13d)–methoxylation (14c & 14d)–demethylation (15c & 15d)–oxidation (5 & 6), respectively. These sequential synthetic transformations efficiently converted olefins 11c & 11d into the corresponding natural products *N*-Me-carbazoquinocin E 5 and *N*-Me-carbazoquinocin F 6, respectively (Scheme 3).

**Scheme 4:**
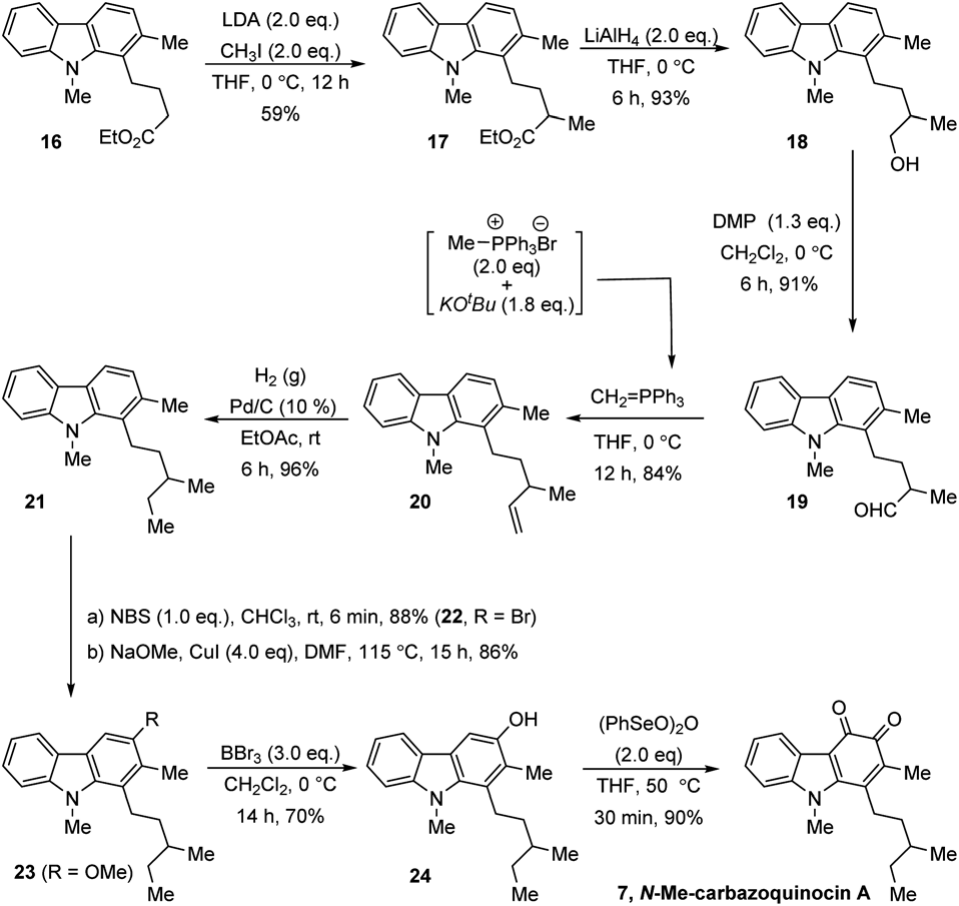
Extension to the total synthesis of *N*-Me-carbazoquinocin A.

Finally, we also extended this strategy for the total synthesis of *N*-Me-carbazoquinocin A 7, which possesses a 3-methylpentyl alkyl chain at the C_1_-position of the carbazoquinone (Scheme 4). We envisioned carbazole ester (16) as a suitable starting material for this purpose. α-Methylation (for *sec*-methyl) followed by conversion of the carboxylate group into an ethyl group generates the required 3-methylpentyl chain at the C_1_-position. Accordingly, the LDA-mediated enolate formation of ester 16 followed by quenching with MeI gave α-methyl ester (17) in 59% yield. The reduction of 17 with LiAlH_4_ followed by oxidation of the resultant primary alcohol (18) with Dess–Martin periodinane (DMP) gave aldehyde (19) in 84% overall yield for two steps.

Further, olefination of aldehyde 19 using methylidene-phosphorous ylide, followed by hydrogenation of the resultant olefin (20) gave 1-[3-methylpentyl] carbazole (21). Subsequently, we employed a four-step synthetic strategy, which involved bromination (22)–methoxylation (23)–demethylation (24)–oxidation (7). These sequential synthetic transformations efficiently converted carbazole 21 into *N*-Me-carbazoquinocin A 7 with an overall yield of 48% for four steps.

## Conclusions

In conclusion, we developed an efficient and practically viable synthetic approach for the unified synthesis of 3,4-carbazoquinone-based carbazole alkaloids *N*-Me-carbazoquinocins A, B and D–F. Our earlier methodology, an acid-catalyzed, intramolecular benzannulation of indole-appended *Z*-enoate propargylic alcohols, was employed for the construction of the required carbazole framework. All five natural products were obtained in an overall yield of 50–60%, starting from the commercially available indole.

## Experimental section

### General information

All solvents were distilled prior to use and anhydrous solvents were prepared according to the standard drying procedures. All non-aqueous reactions were carried out under an atmosphere of nitrogen in flame-dried glassware. Commercially available chemicals were purchased from Sigma-Aldrich, Alfa Aesar and Spectrochem Pvt. Ltd and were used as received without further purification. Infrared (IR) spectra were recorded on a JASCO 4100 FT-IR spectrometer. ^1^H NMR spectra were measured on a Bruker AVANCE 400 MHz or Bruker AVANCE 500 MHz spectrometer. Chemical shifts are reported in ppm relative to solvent signals. ^13^C NMR spectra were recorded on a Bruker AVANCE 100 MHz or Bruker AVANCE 125 MHz spectrometer with complete proton decoupling. Chemical shifts are reported in ppm from the residual solvent as an internal standard [CDCl_3_*δ* = 7.26 ppm for ^1^H, *δ* = 77.16 ppm for ^13^C or calibrated to tetramethylsilane (*δ* = 0.00)]. The following abbreviations are used to indicate multiplicities: s-singlet; d-doublet; t-triplet; q-quartet; quint-quintet; sext-sextet; sept-septet; m-multiplet; dd-doublet of doublet; dt-doublet of triplet; dq-doublet of quartet; td-triplet of doublet; tt-triplet of triplet; dq-doublet of quartet; br-broad; *J*-coupling constant in Hz. The coupling constant *J* (Hz) was rounded to one decimal place for all compounds. When a coupling pattern can be assigned as a combination of multiplicities, the above-mentioned abbreviations were combined to describe the observed patterns (*i.e.*, dt, doublet of triplets). Mass spectra were recorded by the electrospray ionization (ESI) method on a Q-TOF Micro with a lock spray source. For thin layer chromatography (TLC) analysis throughout this work, E-Merck precoated TLC plates (silica gel 60 F254 grade, 0.25 mm) were used and visualized using a UV lamp (366 or 254 nm) or by using of one of the following visualization reagents: PMA: 1 g phosphomolybdic acid/10 mL ethanol; KMnO_4_: 0.15 g potassium permanganate, 1 g K_2_CO_3_/20 mL water. Acme (India) silica gel (100–200 mesh) was used for column chromatography.

#### General procedure I (GP-I): Wittig olefination with primary alkyl-based phosphonium salts and ^*t*^BuOK base^9,4*d*^

RPPh_3_X (2.0 equiv.) and anhydrous THF (8 mL) were added to a flame-dried round-bottom flask under an N_2_ atmosphere. After the mixture was cooled to 0 °C, ^*t*^BuOK (1.8 equiv.) was solubilized in anhydrous THF (4 mL) and added dropwise to the reaction mixture over a period of 5 min. The resulting mixture was warmed to room temperature and stirred for 1 h. Then the reaction mixture was cooled back 0 °C, and a solution of aldehyde 2 (1.0 equiv.) in anhydrous THF (5 mL) was added dropwise. The final reaction mixture was stirred at room temperature until the TLC showed the complete consumption of the aldehyde. After completion, saturated NH_4_Cl solution (6 mL) was added to quench the reaction at 0 °C. Next, diethyl ether (5 mL) and water (5 mL) were added. The color of the mixture changed from pale-yellow to white. After separation of the layers, the residual compound from the aqueous layer was extracted with EtOAc (3 × 5 mL). The combined organic layers were dried over anhydrous Na_2_SO_4_, filtered, and concentrated *in vacuo*. Purification of the crude product using silica gel column chromatography (9 : 1 hexanes/EtOAc) provided the corresponding alkene.

#### General procedure II (GP-II): Wittig olefination with secondary alkyl-based phosphonium salts and *n*-BuLi base^9^

RPPh_3_X (2.0 equiv.) and anhydrous THF (8 mL) were added to a flame-dried round-bottom flask under an N_2_ atmosphere. After the mixture was cooled to 0 °C, *n*-BuLi (3.0 equiv., 1.6 M in hexane) was solubilized in anhydrous THF (4 mL) and added dropwise to the reaction mixture over a period of 5 min. The resulting mixture was warmed to room temperature and stirred for 1 h. Then the reaction mixture was cooled back 0 °C, and a solution of aldehyde 2 (1.0 equiv.) in anhydrous THF (5 mL) was added dropwise. The final reaction mixture was stirred at room temperature until TLC showed the complete consumption of the aldehyde. After completion, saturated NH_4_Cl solution (6 mL) was added to quench the reaction at 0 °C. Next, diethyl ether (5 mL) and water (5 mL) were added. The colour of the mixture changed from pale yellow to white. After separation of the layers, the residual compound from the aqueous layer was extracted with EtOAc (3 × 5 mL). The combined organic layers were dried over anhydrous Na_2_SO_4_, filtered, and concentrated *in vacuo*. Purification of the crude product using silica gel column chromatography (9 : 1 hexanes/EtOAc) provided the corresponding alkene.

#### General procedure III (GP-III): hydrogenation of internal alkenes using Pd/C

Pd/C (10 w/w%) was added to a well-stirred solution of internal alkenes (1.0 equiv.) in EtOAc (10 mL). The resulting reaction mixture was stirred under hydrogen (1 atm) atmosphere for an appropriate time at room temperature. After completion, the reaction mixture was filtered through a Celite® pad by using EtOAc (20 mL). The filtrate was concentrated *in vacuo*. Purification of crude product using a silica gel column chromatography (hexanes/EtOAc) provided the desired reduced product.

#### General procedure IV (GP-IV): bromination of carbazoles using *N*-bromosuccinimide (NBS)

*N*-Bromosuccinimide (1.0 equiv.) was added a solution of carbazole derivative (1.0 equiv.) in chloroform (CHCl_3_, 10 mL) at room temperature under a nitrogen atmosphere. The reaction mixture was stirred at the same temperature for 6 min until TLC showed the complete consumption of the starting material. After completion, water (10 mL) was added. The residual compound from the aqueous layer was extracted with CH_2_Cl_2_ (3 × 5 mL). The combined organic layers were dried over anhydrous Na_2_SO_4_, filtered, and concentrated *in vacuo*. Purification of the crude product using silica gel column chromatography (hexanes/EtOAc) provided the desired 3-bromocarbazole derivative.

#### General procedure V (GP-V): methoxylation using NaOMe

A 15 mL Schlenk tube equipped with a magnetic stirrer was evacuated, and then backfilled with nitrogen gas. This process was repeated three times. Next, 2 mL of anhydrous MeOH was added, and the reaction vessel was cooled to 0 °C. To this stirring MeOH solvent, sodium metal (54 equiv.) was carefully added in portions under a positive nitrogen pressure to form an ∼2.7 M solution of sodium methoxide (NaOMe) in MeOH. After the complete dissolution of Na in MeOH, the solution became thick and light yellowish. Next, the corresponding 3-bromocarbazole (1.0 equiv.) dissolved in DMF (1.4 mL) and CuI (4.0 equiv.) were added to this freshly prepared NaOMe solution under a nitrogen atmosphere. The resulting reaction mixture was transferred to a preheated oil bath and stirred at 115 °C for 15 h. After the complete consumption of the starting material, as indicated by TLC, the crude reaction mixture was filtered through a short plug of Celite® and washed with EtOAc. The filtrate was sequentially washed with saturated NH_4_Cl solution (5 mL), water (10 mL) and brine (5 mL), dried over anhydrous Na_2_SO_4_ and concentrated *in vacuo*. Purification of the crude product using a silica gel column chromatography (9 : 1 hexanes/EtOAc) provided the desired 3-methoxycarbazole derivatives.

#### General procedure VI (GP-VI): BBr_3_-mediated deprotection of the methoxy group to phenol

A 1 M solution of boron tribromide (BBr_3_) in CH_2_Cl_2_ (3.0 equiv.) was added dropwise to a stirred solution of the corresponding 3-methoxycarbazole derivative (1.0 equiv.) in anhydrous CH_2_Cl_2_ (4 mL) at 0 °C. The reaction mixture was allowed to warm up to room temperature and further stirred for the appropriate time. After completion of the reaction, the reaction mixture was quenched with H_2_O (5 mL). The organic layer was separated, and the residual compound from the aqueous layer was extracted with EtOAc (3 × 5 mL). The combined organic layers were washed with H_2_O, brine and dried (Na_2_SO_4_) and concentrated *in vacuo*. Purification of the crude product using silica gel column chromatography (4 : 1 hexane/EtOAc) provided the desired 3-hydroxycarbazole derivative.

#### General procedure VII (GP-VII): synthesis of *N*-Me carbazoquinocin natural products

(PhSeO)_2_O (2.0 equiv.) was added to a solution of *N*-methyl-2-alkyl-3-hydroxy carbazole (1.0 equiv.) in THF (4 mL) at rt under a nitrogen atmosphere. The reaction mixture was stirred at 50 °C for 30 min. After cooling to room temperature, the mixture was quenched with water, and the residual compound from the aqueous layer was extracted with EtOAc (3 × 5 mL). The combined organic layers were washed with H_2_O, brine and dried (Na_2_SO_4_) and concentrated *in vacuo*. Purification of the crude product using silica gel column chromatography (hexane/EtOAc) provided the desired *N*-methyl-carbazoquinocin natural products.

#### General procedure VIII (GP-VIII): preparation of required Wittig salts^8^

Triphenylphosphine (1.0 mmol) was placed in an oven-dried Schlenk tube and dissolved in 2 mL toluene at room temperature under an N_2_-atmosphere. To this reaction mixture, the required alkyl bromide (1.1 mmol) was added. A white precipitate was formed after stirring and refluxing at 110 °C for 48 h using an oil bath. The reaction mixture was cooled to room temperature, and the phosphonium salt was recrystallized from petroleum ether and ethyl acetate solvent mixture. The salt was separated, washed with Et_2_O, and dried *in vacuo* to give the corresponding phosphonium bromide.

#### 2,9-Dimethyl-1-(5-methylhex-4-en-1-yl)-9*H*-carbazole (11a)

Following GP-II. *n*-BuLi (0.22 mL, 0.36 mmol, 3.0 equiv., 1.6 M in hexane) was added to a solution of isopropyltriphenylphosphonium bromide 10a (208 mg, 0.48 mmol, 4.0 equiv.) in anhydrous THF (5 mL), followed by aldehyde 2 (32 mg, 0.12 mmol, 1.0 equiv.) and stirred for 12 h. The corresponding alkene product 11a was isolated as a colourless gummy compound (26 mg, 0.08 mmol, 76%) using hexane/EtOAc (9 : 1) as the eluent.

^1^H NMR (400 MHz, CDCl_3_) *δ* 8.02 (d, *J* = 7.7 Hz, 1H), 7.84 (d, *J* = 7.8 Hz, 1H), 7.44 (dd, *J*_1_ = 7.6 Hz and *J*_2_ = 7.2 Hz, 1H), 7.36 (d, *J* = 8.2 Hz, 1H), 7.20 (dd, *J*_1_ = 7.6 Hz and *J*_2_ = 7.2 Hz, 1H), 7.06 (d, *J* = 7.8 Hz, 1H), 5.26 (t, *J* = 7.0 Hz, 1H), 4.07 (s, 3H), 3.17–3.07 (m, 2H), 2.51 (s, 3H), 2.22 (q, *J* = 7.1 Hz, 2H), 1.75 (s, 3H), 1.72 (d, *J* = 8.6 Hz, 1H), 1.68 (s, 3H) and 1.61–1.57 (m, 1H) ppm.

^13^C[^1^H] NMR (100 MHz, CDCl_3_) *δ* 142.2, 140.0, 134.6, 132.5, 125.3, 124.1, 124.1, 123.2, 122.7, 122.4, 119.6, 118.9, 117.6, 108.7, 32.6, 31.7, 28.4, 28.1, 25.9, 20.2 and 17.9 ppm.

IR (ATR): 2956, 1423, 1421, 1389, 1265 and 749 cm^−1^.

HRMS (ESI) *m*/*z*: [M + H]^+^ calcd for C_21_H_26_N 292.2060; found 292.2063 (1.0 ppm).

TLC: *R*_f_ = 0.4 (19 : 1 hexane/EtOAc).

#### 2,9-Dimethyl-1-(5-methylhexyl)-9*H*-carbazole (12a)

Following GP-III. Pd/C (3 mg, 10 w/w%) was added to a stirred solution of olefin 11a (24 mg, 0.08 mmol, 1.0 equiv.) in EtOAc (4 mL). The resulting reaction mixture was stirred under a hydrogen (1 atm) atmosphere for 6 h at room temperature. This reaction mixture was filtered and purified using silica gel column chromatography (19 : 1 hexanes/EtOAc) to provide the desired hydrogenation product 12a (23 mg, 0.07 mmol, 96%) as a gummy compound.

^1^H NMR (500 MHz, CDCl_3_) *δ* 7.89 (d, *J* = 7.7 Hz, 1H), 7.71 (d, *J* = 7.8 Hz, 1H), 7.30 (dd, *J*_1_ = 7.0 Hz and *J*_2_ = 8.0 Hz, 1H), 7.21 (d, *J* = 8.2 Hz, 1H), 7.08 (dd, *J*_1_ = 7.0 Hz and *J*_2_ = 7.5 Hz, 1H), 6.93 (d, *J* = 7.8 Hz, 1H), 3.90 (s, 3H), 3.00–2.93 (m, 2H), 2.39 (s, 3H), 1.54–1.52 (m, 3H), 1.40–1.38 (m, 2H), 1.25 (q, *J* = 7.8 Hz, 2H) and 0.81 (d, *J* = 6.6 Hz, 6H) ppm.

^13^C[^1^H] NMR (125 MHz, CDCl_3_) *δ* 142.3, 140.0, 134.5, 125.3, 124.3, 123.2, 122.7, 122.5, 119.6, 118.9, 117.6, 108.7, 39.0, 32.6, 32.0, 28.5, 28.2, 27.9, 22.8 and 20.2 ppm.

IR (ATR): 3451, 2928, 2854, 1666, 1589, 1453, 1443, 1362, 1324, 1243, 1096, 826 and 738 cm^−1^.

HRMS (ESI) *m*/*z*: [M + H]^+^ calcd for C_21_H_28_N 294.2216; found 294.2224 (3.0 ppm).

TLC: *R*_f_ = 0.5 (hexane).

#### 3-Bromo-2,9-dimethyl-1-(5-methylhexyl)-9*H*-carbazole (13a)

Following GP-IV. *N*-Bromosuccinimide (26 mg, 0.14 mmol, 1.0 equiv.) was added to a solution of carbazole 12a (42 mg, 0.14 mmol, 1.0 equiv.) in chloroform (CHCl_3_) (6 mL) under a nitrogen atmosphere. The reaction mixture was stirred for 6 min. After completion, water (10 mL) was added and extracted with CH_2_Cl_2_ (3 × 5 mL). The combined organic layers were dried and concentrated *in vacuo*. Purification of the crude product using silica gel column chromatography (19 : 1 hexanes/EtOAc) provided the desired product 13a (48 mg, 0.12 mmol, 90%) as a white solid.

^1^H NMR (400 MHz, CDCl_3_) *δ* 8.14 (s, 1H), 7.97 (d, *J* = 7.7 Hz, 1H), 7.46 (t, *J* = 7.6 Hz, 1H), 7.35 (d, *J* = 8.2 Hz, 1H), 7.22 (t, *J* = 7.3 Hz, 1H), 4.01 (s, 3H), 3.18–3.07 (m, 2H), 2.59 (s, 3H), 1.68–1.59 (m, 3H), 1.56–1.49 (m, 2H), 1.30 (d, *J* = 8.2 Hz, 2H) and 0.94 (d, *J* = 6.6 Hz, 6H) ppm.

^13^C[^1^H] NMR (100 MHz, CDCl_3_) *δ* 142.5, 139.0, 132.9, 126.0, 123.7, 122.1, 121.6, 119.8, 119.3, 116.8, 108.9, 38.8, 32.7, 32.0, 29.6, 28.1, 27.8, 22.8 and 19.8 ppm.

IR (ATR): 2964, 2846, 2365, 1456, 1413, 1271, 1144, 1016, 828, 773 and 736 cm^−1^.

HRMS (ESI) *m*/*z*: [M + H]^+^ calcd for C_21_H_27_NBr 372.1321; found 372.1326 (1.3 ppm).

TLC: *R*_f_ = 0.55 (hexane).

M.P.: 173–175 °C.

#### 3-Methoxy-2,9-dimethyl-1-(5-methylhexyl)-9*H*-carbazole (14a)

Following GP-V. A freshly prepared NaOMe solution (∼2.7 M in MeOH) was added to a DMF (1.6 mL) solution of 3-bromocarbazole 13a (38 mg, 0.1 mmol, 1.0 equiv.), followed by CuI (77 mg, 0.39 mmol, 4.0 equiv.), and the reaction mixture was stirred at 115 °C for 15 h. The reaction mixture was filtered, and the filtrate was sequentially washed with saturated NH_4_Cl solution (5 mL), water (10 mL) and brine (5 mL), dried concentrated *in vacuo*. Purification of the crude product *via* silica gel column chromatography (9 : 1 hexanes/EtOAc) provided the desired 3-methoxycarbazole 14a (29 mg, 0.09 mmol, 90%) as a colorless solid.

^1^H NMR (500 MHz, CDCl_3_) *δ* 7.99 (d, *J* = 7.7 Hz, 1H), 7.44–7.39 (m, 2H), 7.36 (d, *J* = 8.2 Hz, 1H), 7.17 (t, *J* = 7.3 Hz, 1H), 4.05 (s, 3H), 3.95 (s, 3H), 3.16–3.10 (m, 2H), 2.38 (s, 3H), 1.70–1.63 (m, 2H), 1.60 (dd, *J* = 13.3, 6.7 Hz, 1H), 1.52–1.31 (m, 2H), 1.29–1.26 (m, 2H) and 0.92 (d, *J* = 6.6 Hz, 6H) ppm.

^13^C[^1^H] NMR (125 MHz, CDCl_3_) *δ* 152.4, 142.5, 134.9, 125.8, 125.1, 124.8, 123.3, 121.9, 119.5, 118.5, 108.8, 99.3, 56.3, 39.0, 32.8, 31.9, 28.8, 28.2, 27.9, 22.8 and 12.1 ppm.

IR (ATR): 2945, 2856, 2392, 1471, 1414, 1288, 1213, 1154, 1126, 845 and 742 cm^−1^.

HRMS (ESI) *m*/*z*: [M + H]^+^ calcd for C_22_H_30_NO 324.2322; found 324.2342 (6.1 ppm).

TLC: *R*_f_ = 0.2 (hexane).

M.P.: 162–164 °C.

#### 2,9-Dimethyl-1-(5-methylhexyl)-9*H*-carbazol-3-ol (15a)

Following GP-VI. A 1 M solution of boron tribromide (BBr_3_, in CH_2_Cl_2_; 0.33 mL, 0.33 mmol, 4.0 equiv.) was added a stirring solution of 3-methoxycarbazole 14a (27 mg, 0.08 mmol, 1.0 equiv.) in anhydrous CH_2_Cl_2_ (4 mL) at 0 °C and further stirred for 11 h. The reaction was quenched with H_2_O (5 mL) and extracted with CH_2_Cl_2_ (3 × 5 mL). The combined organic layers were dried over (Na_2_SO_4_) and concentrated *in vacuo*. Purification of the crude product using silica gel column chromatography (4 : 1 hexanes/EtOAc) provided phenol 15a (21 mg, 0.07 mmol, 82% yield) as a pale-yellow liquid.

^1^H NMR (400 MHz, CDCl_3_) *δ* 7.91 (d, *J* = 7.7 Hz, 1H), 7.42 (t, *J* = 7.6 Hz, 1H), 7.34 (d, *J* = 7.8 Hz, 2H), 7.15 (t, *J* = 7.3 Hz, 1H), 4.03 (s, 3H), 3.16–3.08 (m, 2H), 2.40 (s, 3H), 1.70–1.58 (m, 3H), 1.53 (dd, *J* = 15.0, 7.2 Hz, 2H), 1.32–1.26 (m, 2H) and 0.92 (d, *J* = 6.6 Hz, 6H) ppm.

^13^C[^1^H] NMR (100 MHz, CDCl_3_) *δ* 147.7, 142.6, 135.1, 125.7, 125.4, 122.8, 122.4, 122.2, 119.7, 118.4, 108.7, 103.1, 38.9, 32.8, 32.0, 28.8, 28.2, 27.9, 22.8 and 12.1 ppm.

IR (ATR): 3325, 2926, 2854, 2363, 2351, 1485, 1451, 1264, 1232, 902, 782 and 731 cm^−1^.

HRMS (ESI) *m*/*z*: [M + H]^+^ calcd for C_21_H_28_NO 310.2165; found 310.2174 (3.0 ppm).

TLC: *R*_f_ = 0.25 (9 : 1 hexane/EtOAc).

#### 2,9-Dimethyl-1-(5-methylhexyl)-3*H*-carbazole-3,4(9*H*)-dione[*N*-methylcarbazoquinocin B] (3)

Following GP-VII. A solution of carbazol-3-ol 15a (20 mg, 0.06 mmol) and (PhSeO)_2_O (47.0. mg, 0.13 mmol, 2.0 equiv.) in THF (4 mL) was stirred at 50 °C for 30 min. The mixture was quenched with water and the residual compound from the aqueous layer was extracted with EtOAc (3 × 5 mL). The combined organic layers were dried (Na_2_SO_4_) and concentrated *in vacuo*. Purification of the crude product using silica gel column chromatography (3 : 1 hexanes/EtOAc) provided the desired *N*-methyl carbazoquinocin B 3 (20 mg, 0.06 mmol 95% yield) as a dark-brown glittering solid.

^1^H NMR (400 MHz, CDCl_3_) *δ* 8.04 (s, 1H), 7.18 (s, 3H), 3.80 (s, 3H), 2.68–2.55 (m, 2H), 1.82 (s, 3H), 1.49 (d, *J* = 5.4 Hz, 3H), 1.42 (d, *J* = 6.7 Hz, 2H), 1.18 (s, 2H) and 0.83 (d, *J* = 6.5 Hz, 6H) ppm.

^13^C[^1^H] NMR (100 MHz, CDCl_3_) *δ* 183.2, 173.6, 144.9, 142.6, 139.6, 134.5, 125.7, 124.9, 121.8, 113.7, 110.9, 38.7, 33.1, 29.9, 28.6, 28.1, 27.8, 22.7 and 11.9 ppm.

IR (ATR): 2961, 2942, 2863, 1665, 1652, 1632, 1499, 1481, 1459, 1369, 1234 and 781 cm^−1^.

HRMS (ESI) *m*/*z*: [M + H]^+^ calcd for C_21_H_26_NO_2_ 324.1958; found 324.1935 (7.0 ppm).

TLC: *R*_f_ = 0.34 (4 : 1 hexanes/EtOAc).

M.P.: 152–154 °C.

#### 2,9-Dimethyl-1-(5-methylhept-4-en-1-yl)-9*H*-carbazole (11b)

Following GP-II. *n*-BuLi (0.19 mL, 0.31 mmol, 3.0 equiv., 1.6 M in hexane) was added to a solution of *sec*-butyltriphenylphosphonium bromide 10b (144 mg, 0.42 mmol, 4.0 equiv.) in anhydrous THF (5 mL), followed by aldehyde 2 (28 mg, 0.10 mmol, 1.0 equiv.) and stirred for 12 h. The corresponding alkene product 11b was isolated as a colorless liquid (23.3 mg, 0.07 mmol, 76%) using hexanes/EtOAc (9 : 1) as the eluent.

^1^H NMR (400 MHz, CDCl_3_) major isomer: *δ* 7.93 (d, *J* = 7.6 Hz, 1H), 7.74 (d, *J* = 7.7 Hz, 1H), 7.34 (dd, *J*_1_ = 7.6 Hz and *J*_2_ = 8.0 Hz, 1H), 7.27 (d, *J* = 8.1 Hz, 1H), 7.11 (t, *J* = 7.3 Hz, 1H), 6.96 (d, *J* = 7.7 Hz, 1H), 5.22–5.10 (m, 1H), 3.97 (s, 3H), 3.09–2.98 (m, 2H), 2.42 (s, 3H), 2.14 (d, *J* = 5.3 Hz, 2H), 2.06–1.90 (m, 2H), 1.65 (s, 3H), 1.59 (s, 2H) and 1.00–0.88 (m, 3H) ppm.

^1^H NMR (400 MHz, CDCl_3_) minor isomer: *δ* 7.93 (d, *J* = 7.6 Hz, 1H), 7.74 (d, *J* = 7.7 Hz, 1H), 7.34 (dd, *J*_1_ = 7.6 Hz and *J*_2_ = 8.0 Hz, 1H), 7.27 (d, *J* = 8.1 Hz, 1H), 7.11 (t, *J* = 7.3 Hz, 1H), 6.96 (d, *J* = 7.7 Hz, 1H), 5.04–4.94 (m, 1H), 3.97 (s, 3H), 3.09–2.98 (m, 2H), 2.42 (s, 3H), 2.14 (d, *J* = 5.3 Hz, 2H), 2.06–1.90 (m, 2H), 1.65 (s, 3H), 1.59 (s, 2H) and 1.00–0.88 (m, 3H) ppm.

^13^C[^1^H] NMR (100 MHz, CDCl_3_) *δ* 142.2, 139.9, 138.2, 138.1, 134.6, 125.3, 124.1, 124.1, 123.6, 123.1, 122.6, 122.4, 119.6, 118.9, 117.6, 108.7, 32.6, 32.5, 31.9, 31.7, 28.2, 28.1, 28.0, 24.9, 23.0, 20.2, 16.2 and 13.0 ppm.

IR (ATR): 2966, 1445, 1438, 1390, 1268, 1146, 1121 and 748 cm^−1^.

HRMS (ESI) *m*/*z*: [M + NH_4_]^+^ calcd for C_22_H_31_N_2_ 323.2482; found 323.2496 (4.3 ppm).

TLC: *R*_f_ = 0.8 (9 : 1 hexane/EtOAc).

#### 2,9-Dimethyl-1-(5-methylheptyl)-9*H*-carbazole (12b)

Following GP-III. Pd/C (4 mg, 10 wt%) was added to a stirring solution of olefin 11b (37 mg, 0.12 mmol, 1.0 equiv.) in EtOAc (4 mL). The resulting reaction mixture was stirred under a hydrogen (1 atm) atmosphere for 5 h at room temperature. This reaction mixture was filtered and purified using silica gel column chromatography (19 : 1 hexanes/EtOAc) to provide the desired hydrogenation product 12b (34 mg, 0.11 mmol, 91%) as a colorless liquid.

^1^H NMR (400 MHz, CDCl_3_) *δ* 7.93 (d, *J* = 7.5 Hz, 1H), 7.75 (d, *J* = 7.7 Hz, 1H), 7.35 (t, *J* = 7.5 Hz, 1H), 7.27 (d, *J* = 8.1 Hz, 1H), 7.11 (t, *J* = 7.2 Hz, 1H), 6.97 (d, *J* = 7.7 Hz, 1H), 3.98 (s, 3H), 3.08–2.96 (m, 2H), 2.43 (s, 3H), 1.47 (m, 5H), 1.32 (m, 3H), 1.10–1.09 (m, 1H) and 0.81 (d, *J* = 5.8 Hz, 6H) ppm.

^13^C[^1^H] NMR (100 MHz, CDCl_3_) *δ* 142.2, 139.9, 134.5, 125.3, 124.3, 123.2, 122.7, 122.4, 119.6, 118.9, 117.6, 108.7, 36.6, 34.5, 32.6, 32.0, 29.7, 28.6, 27.7, 20.3, 19.4, 11.6 ppm.

IR (ATR): 3421, 2925, 2862, 1642, 1586, 1482, 1452, 1431, 1374, 1361, 1285, 1046, 923 and 722 cm^−1^.

HRMS (ESI) *m*/*z*: [M + H]^+^ calcd for C_22_H_30_N 308.2373; found 308.2382 (3.0 ppm).

TLC: *R*_f_ = 0.45 (hexane).

#### 3-Bromo-2,9-dimethyl-1-(5-methylheptyl)-9*H*-carbazole (13b)

Following GP-IV. *N*-Bromo-succinimide (18 mg, 0.09 mmol, 1.0 equiv.) was added to a solution of carbazole 12b (30 mg, 0.09 mmol, 1.0 equiv.) in chloroform (CHCl_3_) (6 mL) under a nitrogen atmosphere. The reaction mixture was stirred for 6 min. After completion, water (10 mL) was added and extracted with CH_2_Cl_2_ (3 × 5 mL). The combined organic layers were dried and concentrated *in vacuo*. Purification of the crude product *via* silica gel column chromatography (19 : 1 hexanes/EA) provided the desired product 13b (34 mg, 0.08 mmol, 92%) as a gummy compound.

^1^H NMR (400 MHz, CDCl_3_) *δ* 8.08 (s, 1H), 7.90 (d, *J* = 7.7 Hz, 1H), 7.40 (t, *J* = 7.6 Hz, 1H), 7.29 (d, *J* = 8.2 Hz, 1H), 7.15 (t, *J* = 7.1 Hz, 1H), 3.97 (s, 3H), 3.14–3.02 (m, 2H), 2.52 (s, 3H), 1.60–1.45 (m, 5H), 1.32–1.31 (m, 3H), 1.20–1.15 (m, 1H) and 0.84 (d, *J* = 5.7 Hz, 6H) ppm.

^13^C[^1^H] NMR (100 MHz, CDCl_3_) *δ* 142.5, 139.1, 132.9, 126.0, 123.7, 122.1, 121.6, 119.8, 119.3, 116.8, 108.9, 36.5, 34.5, 32.8, 32.0, 29.6, 27.5, 19.8, 19.3, 11.6 ppm.

IR (ATR): 2934, 2868, 2376, 1468, 1402, 1282, 1149, 1016, 812, 762 and 742 cm^−1^.

HRMS (ESI) *m*/*z*: [M + H]^+^ calcd for C_22_H_29_NBr 386.1478; found 386.1460 (5 ppm).

TLC: *R*_f_ = 0.5 (hexane).

#### 3-Methoxy-2,9-dimethyl-1-(5-methylheptyl)-9*H*-carbazole (14b)

Following GP-V. A freshly prepared NaOMe solution (∼2.7 M in MeOH) was added to a DMF (1.6 mL) solution of 3-bromocarbazole 13b (29 mg, 0.07 mmol, 1.0 equiv.), followed by CuI (59 mg, 0.23 mmol, 4.0 equiv.), and the reaction mixture stirred at 115 °C for 15 h. The reaction mixture was filtered, and the filtrate was sequentially washed with saturated NH_4_Cl solution (5 mL), water (10 mL) and brine (5 mL), dried and concentrated *in vacuo*. Purification of the crude product *via* silica gel column chromatography (9 : 1 hexanes/EtOAc) provided the desired 3-methoxycarbazole 14b (23 mg, 0.06 mmol, 90%) as a colourless liquid.

^1^H NMR (500 MHz, CDCl_3_) *δ* 7.91 (d, *J* = 7.7 Hz, 1H), 7.36–7.30 (m, 2H), 7.27 (d, *J* = 8.2 Hz, 1H), 7.08 (t, *J* = 7.3 Hz, 1H), 3.96 (s, 3H), 3.86 (s, 3H), 3.07–3.01 (m, 2H), 2.29 (s, 3H), 1.62–1.42 (m, 5H), 1.36–1.25 (m, 3H), 1.11–0.85 (m, 1H) and 0.84–0.78 (m, 6H) ppm.

^13^C[^1^H] NMR (125 MHz, CDCl_3_) *δ* 152.3, 142.5, 134.8, 125.8, 125.1, 124.8, 123.3, 121.8, 119.5, 118.5, 108.8, 99.2, 56.3, 36.6, 34.6, 32.8, 32.0, 29.7, 28.8, 27.6, 19.4, 12.1 and 11.6 ppm.

IR (ATR): 2929, 2857, 2389, 1471, 1416, 1273, 1220, 1152, 1104, 838 and 741 cm^−1^.

HRMS (ESI) *m*/*z*: [M + H]^+^ calcd for C_23_H_32_NO 338.2478; found 338.2469 (3.0 ppm).

TLC: *R*_f_ = 0.18 (hexane).

#### 2,9-Dimethyl-1-(5-methylheptyl)-9*H*-carbazol-3-ol (15b)

Following GP-VI. To a stirring solution of 3-methoxycarbazole 14b (21 mg, 0.06 mmol, 1.0 equiv.) in anhydrous CH_2_Cl_2_ (4 mL) at 0 °C, a 1 M solution of boron tribromide (BBr_3_) in CH_2_Cl_2_ (0.25 mL, 0.25 mmol, 4.0 equiv.) was added dropwise and further stirred for 8 h. The reaction was quenched with H_2_O (5 mL). The organic layer was extracted with CH_2_Cl_2_ (3 × 5 mL). The combined organic layers were dried over (Na_2_SO_4_) and concentrated *in vacuo*. Purification of the crude product *via* silica gel column chromatography (4 : 1 hexanes/EtOAc) provided the desired phenol 15b (18.2 mg, 0.05 mmol, 91% yield) as a gummy compound.

^1^H NMR (400 MHz, CDCl_3_) *δ* 7.84 (d, *J* = 7.6 Hz, 1H), 7.33 (d, *J* = 7.4 Hz, 1H), 7.26 (s, 2H), 7.07 (t, *J* = 7.2 Hz, 1H), 3.95 (s, 3H), 3.08–2.96 (m, 2H), 2.31 (s, 3H), 1.63–1.40 (m, 5H), 1.28 (s, 2H) and 1.16–1.08 (m, 2H), 0.81–0.80 (m, 6H) ppm.

^13^C[^1^H] NMR (100 MHz, CDCl_3_) *δ* 148.1, 141.3, 135.4, 127.9, 126.0, 124.6, 123.5, 122.3, 121.2, 111.2, 110.3, 103.1, 39.2, 32.9, 31.7, 30.3, 28.7, 28.1, 27.4, 22.8, 12.0 and 11.2 ppm.

IR (ATR): 3326, 2922, 2862, 2396, 1469, 1296, 1245, 1077 and 743 cm^−1^.

HRMS (ESI) *m*/*z*: [M + NH_4_]^+^ calcd for C_22_H_33_N_2_O 341.2587; found 341.2633 (13.4 ppm).

TLC: *R*_f_ = 0.4 (9 : 1, hexanes/EtOAc).

#### 2,9-Dimethyl-1-(5-methylheptyl)-3*H*-carbazole-3,4(9*H*)-dione-[*N*-methylcarbazoquinocin D] 4

Following GP-VII. A solution of carbazol-3-ol 15b (15 mg, 0.05 mmol) and (PhSeO)_2_O (34.0. mg, 0.09 mmol, 2.0 equiv.) in THF (4 mL) was stirred at 50 °C for 30 min, the mixture was quenched with water, and the residual compound from the aqueous layer was extracted with EtOAc (3 × 4 mL). The combined organic layers were dried (Na_2_SO_4_) and concentrated *in vacuo*. Purification of crude product *via* silica gel column chromatography (3 : 1 hexanes/EtOAc) provided the desired *N*-methyl carbazoquinocin D 4 (14.2 mg, 0.04 mmol, 89% yield) as a dark-brown glittering solid.

^1^H NMR (500 MHz, CDCl_3_) *δ* 8.12–7.99 (m, 1H), 7.21–7.14 (m, 3H), 3.82 (s, 2H), 2.63 (t, *J* = 8.1 Hz, 2H), 1.84 (s, 3H), 1.57–1.41 (m, 4H), 1.33–1.24 (m, 3H), 1.15–1.05 (m, 2H) and 0.88–0.74 (m, 6H) ppm.

^13^C[^1^H] NMR (125 MHz, CDCl_3_) *δ* 183.3, 173.7, 144.9, 142.6, 139.6, 134.5, 125.8, 124.9, 121.9, 113.8, 110.8, 36.4, 34.5, 33.1, 29.9, 29.6, 28.7, 27.5, 19.3, 11.9 and 11.5 ppm.

IR (ATR): 2971, 2934, 2893, 1688, 1652, 1632, 1494, 1441, 1414, 1398, 1242 and 765 cm^−1^.

HRMS (ESI) *m*/*z*: [M + Na]^+^ calcd for C_22_H_28_NO_2_ 338.2115; found 338.2116 (0.3 ppm).

TLC: *R*_f_ = 0.3 (8 : 1 hexanes/EtOAc).

M.P.: 148–150 °C.

#### (*Z*)-2,9-Dimethyl-1-(6-methylhept-4-en-1-yl)-9*H*-carbazole (11c)

Following GP-II. ^*t*^BuOK (41 mg, 0.33 mmol, 1.8 equiv.) was added to a solution of isobutyl (triphenyl phosphonium) bromide 10c (128 mg, 0.38 mmol, 2.0 equiv.) in anhydrous THF (5 mL), followed by aldehyde 2 (50 mg, 0.18 mmol, 1.0 equiv.) and stirred for 12 h. The corresponding alkene product 11c was isolated as a colorless gummy compound (46 mg, 0.15 mmol, 81%) using hexanes/EtOAc (9 : 1) as the eluent.

^1^H NMR (500 MHz, CDCl_3_) *δ* 7.91 (d, *J* = 7.5 Hz, 1H), 7.73 (d, *J* = 8.0 Hz, 1H), 7.32 (dd, *J*_1_ = 7.0 Hz and *J*_2_ = 8.0 Hz, 1H), 7.24 (d, *J* = 8.0 Hz, 1H), 7.08 (dd, *J*_1_ = 7.5 Hz and *J*_2_ = 1.0 Hz, 1H), 6.94 (d, *J* = 8.0 Hz, 1H), 5.27–5.18 (m, 2H), 3.94 (s, 3H), 3.03–3.00 (m, 2H), 2.60–2.58 (m, 1H), 2.41 (s, 3H), 2.17 (q, *J* = 7.5 Hz, 2H), 1.65–1.62 (m, 2H) and 0.89 (q, *J* = 2.5 Hz, 6H) ppm.

^13^C[^1^H] NMR (125 MHz, CDCl_3_) *δ* 142.3, 140.0, 138.7, 134.6, 126.5, 125.3, 124.0, 123.2, 122.8, 122.5, 119.7, 119.0, 117.7, 108.7, 32.7, 31.7, 28.1, 27.7, 26.7, 23.3 and 20.2 ppm.

IR (ATR): 2917, 1486, 1485, 1325, 1235 and 756 cm^−1^.

HRMS (ESI) *m*/*z*: [M + H]^+^ calcd for C_22_H_28_N 306.2216; found 306.2212 (1.3 ppm).

TLC: *R*_f_ = 0.4 (19 : 1 hexane/EtOAc).

#### 2,9-Dimethyl-1-(6-methylheptyl)-9*H*-carbazole (12c)

Following GP-III. Pd/C (4 mg, 10 wt%) was added to a stirred solution of olefin 11c (40 mg, 0.13 mmol, 1.0 equiv.) in EtOAc (4 mL). The resulting reaction mixture was stirred under a hydrogen (1 atm) atmosphere for 4 h at room temperature. The reaction mixture was filtered and purification of the crude product *via* silica gel column chromatography (19 : 1 hexanes/EtOAc) provided the desired hydrogenation product 12c (37 mg, 0.12 mmol, 93%) as a colorless liquid.

^1^H NMR (500 MHz, CDCl_3_) *δ* 8.04 (d, *J* = 7.5 Hz, 1H), 7.85 (dt, *J*_1_ = 7.5 Hz, *J*_2_ = 2.0 Hz, 1H), 7.45 (t, 1H), 7.37 (d, *J* = 8.0 Hz, 1H), 7.23–7.20 (m, 1H), 7.07 (d, *J* = 7.5 Hz, 1H), 4.08 (s, 3H), 3.14–3.13 (m, 2H), 2.53 (s, 3H), 1.72–1.69 (m, 2H), 1.56–1.53 (m, 1H), 1.52–1.51 (m, 2H), 1.43–1.41 (m, 2H) and 1.27–1.24 (m, 2H), 0.92–0.91 (m, 6H) ppm.

^13^C[^1^H] NMR (125 MHz, CDCl_3_) *δ* 142.3, 140.0, 134.5, 125.3, 124.3, 123.3, 122.8, 122.5, 119.6, 118.9, 117.6, 108.7, 39.2, 32.6, 31.7, 30.4, 28.5, 28.1, 27.4, 22.8 and 20.2 ppm.

IR (ATR): 3426, 2986, 2843, 1642, 1531, 1479, 1413, 1372, 1324, 1253, 1026, 866 and 732 cm^−1^.

HRMS (ESI) *m*/*z*: [M + H]^+^ calcd for C_22_H_30_N 308.2373; found 308.2324 (16.0 ppm).

TLC: *R*_f_ = 0.4 (hexane).

#### 3-Bromo-2,9-dimethyl-1-(6-methylheptyl)-9*H*-carbazole (13c)

Following GP-IV. *N*-Bromo-succinimide (20 mg, 0.11 mmol, 1.0 equiv.) was added to a solution of carbazole 12c (35 mg, 0.11 mmol, 1.0 equiv.) in chloroform (CHCl_3_) (6 mL) under a nitrogen atmosphere. The reaction mixture was stirred for 6 min. After completion, water (10 mL) was added and extracted with CH_2_Cl_2_ (3 × 5 mL). The combined organic layers were dried and concentrated *in vacuo*. Purification of the crude product *via* silica gel column chromatography (19 : 1 hexanes/EA) provided the desired product 13c (38 mg, 0.10 mmol, 91%) as a gummy compound.

^1^H NMR (500 MHz, CDCl_3_) *δ* 8.14 (s, 1H), 7.96 (d, *J* = 7.5 Hz, 1H), 7.47 (dd, *J*_1_ = 7.5 Hz and *J*_2_ = 1.0 Hz, 1H), 7.34 (d, *J* = 8.0 Hz, 1H), 7.21 (dd, *J*_1_ = 8.0 Hz and *J*_2_ = 7.0 Hz, 1H), 4.00 (s, 3H), 3.14–3.11 (m, 2H), 2.58 (s, 3H), 1.67–1.65 (m, 2H), 1.58–1.52 (m, 1H), 1.51–1.49 (m, 2H), 1.42–1.38 (m, 2H) and 1.26–1.23 (m, 2H), 0.91 (d, *J* = 6.5 Hz, 6H) ppm.

^13^C[^1^H] NMR (125 MHz, CDCl_3_) *δ* 142.6, 139.1, 133.0, 126.0, 126.1, 123.8, 122.2, 121.6, 119.8, 119.3, 116.9, 108.9, 39.1, 32.8, 31.7, 30.3, 29.6, 28.1, 27.4, 22.8 and 19.8 ppm.

IR (ATR): 2923, 2857, 2396, 1462, 1406, 1274, 1134, 1056, 829, 762 and 746 cm^−1^.

HRMS (ESI) *m*/*z*: [M + H]^+^ calcd for C_22_H_29_NBr 386.1478; found 386.1432 (12 ppm).

TLC: *R*_f_ = 0.5 (hexane).

#### 3-Methoxy-2,9-dimethyl-1-(6-methylheptyl)-9*H*-carbazole (14c)

Following GP-V. A freshly prepared NaOMe solution (∼2.7 M in MeOH) was added to a DMF (1.6 mL) solution of 3-bromocarbazole 13c (35 mg, 0.09 mmol, 1.0 equiv.), followed by CuI (72 mg, 0.36 mmol, 4.0 equiv.), and the reaction mixture stirred at 115 °C for 15 h. The reaction mixture was filtered, and the filtrate was sequentially washed with saturated NH_4_Cl solution (5 mL), water (10 mL) and brine (5 mL), dried and concentrated *in vacuo*. Purification of the crude product *via* silica gel column chromatography (9 : 1 hexanes/EtOAc) provided the desired 3-methoxycarbazole 14c (31 mg, 0.09 mmol, 93%) as a yellowish liquid.

^1^H NMR (500 MHz, CDCl_3_) *δ* 7.97 (d, *J* = 7.5 Hz, 1H), 7.4–7.40 (m, 2H), 7.35 (d, *J* = 8 Hz, 1H), 7.16 (dd, *J*_1_ = 7 Hz and *J*_2_ = 7.5 Hz, 1H), 4.04 (s, 3H), 3.94 (s, 3H), 3.12 (t, 2H), 2.37 (s, 3H), 1.55–1.51 (m, 2H), 1.52–1.50 (m, 3H), 1.39–1.31 (m, 2H), 1.26–1.21 (m, 2H) and 0.88 (d, *J* = 6.5 Hz, 6H) ppm.

^13^C[^1^H] NMR (125 MHz, CDCl_3_) *δ* 152.4, 142.5, 134.9, 125.8, 125.1, 124.8, 123.3, 121.8, 119.5, 118.5, 108.8, 99.3, 56.3, 39.2, 32.8, 31.7, 30.4, 28.8, 28.1, 27.4, 22.8 and 12.1 ppm.

IR (ATR): 2922, 2879, 2362, 1471, 1424, 1288, 1212, 1144, 1109, 834 and 732 cm^−1^.

HRMS (ESI) *m*/*z*: [M + H]^+^ calcd for C_23_H_32_NO 338.2478; found 338.2443 (10.3 ppm).

TLC: *R*_f_ = 0.2 (hexane).

#### 2,9-Dimethyl-1-(6-methylheptyl)-9*H*-carbazol-3-ol (15c)

Following GP-VI. To a stirring solution of 3-methoxycarbazole 14c (28 mg, 0.08 mmol, 1.0 equiv.) in anhydrous CH_2_Cl_2_ (4 mL) at 0 °C, a 1 M solution of boron tribromide (BBr_3_) in CH_2_Cl_2_ (0.33 mL, 0.33 mmol, 4.0 equiv.) was added dropwise, and further stirred for 12 h. The reaction was quenched with H_2_O (5 mL). The organic layer was extracted with CH_2_Cl_2_ (3 × 5 mL). The combined organic layers were dried (Na_2_SO_4_) and concentrated *in vacuo*. Purification of the crude product *via* silica gel column chromatography (4 : 1 hexanes/EtOAc) provided the desired phenol 15c (25.5 mg, 0.08 mmol, 95% yield) as a white gummy compound.

^1^H NMR (500 MHz, CDCl_3_) *δ* 7.91 (d, *J* = 8.0 Hz, 1H), 7.41 (dd, *J*_1_ = 8.0 Hz and *J*_2_ = 7.0 Hz, 1H), 7.34–7.33 (m, 2H), 7.14 (dd, *J*_1_ = 7.5 Hz and *J*_2_ = 7.0 Hz, 1H), 4.03 (s, 3H). 3.12 (t, 2H), 2.39 (s, 3H), 1.67–1.64 (m, 2H), 1.58–157 (m, 1H), 1.54–1.50 (m, 2H), 1.39–1.37 (m, 2H), 1.26–1.21 (m, 2H) and 0.88 (d, *J* = 6.5 Hz, 6H) ppm.

^13^C[^1^H] NMR (125 MHz, CDCl_3_) *δ* 148.1, 141.3, 135.4, 127.9, 126.0, 124.6, 123.5, 122.3, 121.2, 111.2, 110.3, 103.1, 39.2, 32.9, 31.7, 30.3, 28.7, 28.1, 27.4, 22.8 and 12.2 ppm.

IR (ATR): 3345, 2934, 2859, 2359, 2346, 1485, 1449, 1277, 1236, 929, 782 and 734 cm^−1^.

HRMS (ESI) *m*/*z*: [M + H]^+^ calcd for C_22_H_30_NO 324.2322; found 324.2278 (13.5 ppm).

TLC: *R*_f_ = 0.2 (9 : 1 hexane/EtOAc).

#### 2,9-Dimethyl-1-(6-methylheptyl)-3*H*-carbazole-3,4(9*H*)dione-[*N*-methyl carbazoquinocin E] 5

Following GP-VII. A solution of carbazol-3-ol 15c (7 mg, 0.02 mmol) and (PhSeO)_2_O (16.0. mg, 0.04 mmol, 2.0 equiv.) in THF (4 mL) was stirred at 50 °C for 30 min. The mixture was quenched with water, and the residual compound from the aqueous layer was extracted with EtOAc (3 × 4 mL). The combined organic layers were dried (Na_2_SO_4_) and concentrated *in vacuo*. Purification of the crude product *via* silica gel column chromatography (3 : 1 hexanes/EtOAc) provided the desired *N*-methyl carbazoquinocin E 5 (6.6 mg, 0.02 mmol, 91% yield) as a dark-brown glittering solid.

^1^H NMR (500 MHz, CDCl_3_) *δ* 8.09–8.08 (m, 1H), 7.20–7.20 (m, 3H), 3.84 (s, 3H), 2.66–2.64 (m, 2H), 1.88 (s, 3H), 1.58–1.52 (m, 3H), 1.47 (d, *J* = 6.6 Hz, 1H), 1.43–1.37 (m, 2H), 1.33–1.28 (m, 2H), 1.17–1.12 (m, 2H) and 0.81 (d, *J* = 6.5 Hz, 6H) ppm.

^13^C[^1^H] NMR (125 MHz, CDCl_3_) *δ* 183.4, 173.8, 145.0, 142.6, 139.7, 134.6, 125.9, 124.9, 122.0, 113.9, 110.7, 39.0, 33.1, 30.2, 30.0, 28.5, 28.1, 27.2, 22.7 and 12.0 ppm.

IR (ATR): 2952, 2934, 2863, 1658, 1647, 1635, 1482, 1463, 1434, 1372, 1234 and 762 cm^−1^.

HRMS (ESI) *m*/*z*: [M + Na]^+^ calcd for C_22_H_27_NNaO_2_ 360.1934; found 360.1964 (8.3 ppm).

TLC: *R*_f_ = 0.4 (4 : 1 hexane/EtOAc).

M.P.: 156–158 °C.

#### 2,9-Dimethyl-1-(7-methyloct-4-en-1-yl)-9*H*-carbazole (11d)

Following GP-II. ^*t*^BuOK (18 mg, 0.14 mmol, 1.8 equiv.) was added to a solution of (4-methylpentyl) triphenyl phosphonium bromide 10d (80 mg, 0.18 mmol, 2.0 equiv.) in anhydrous THF (5 mL), followed by aldehyde 2 (25 mg, 0.09 mmol, 1.0 equiv.) and stirred for 12 h. The corresponding alkene product 11d was isolated as a pale-yellow gummy compound (23 mg, 0.07 mmol, 80%) using hexane/EtOAc (9 : 1) as the eluent.

^1^H NMR (500 MHz, CDCl_3_) *δ* 7.91 (d, *J* = 7.5 Hz, 1H), 7.73 (d, *J* = 8.0 Hz, 1H), 7.33 (dd, *J*_1_ = 8.5 Hz and *J*_2_ = 7.0 Hz, 1H), 7.24–7.23 (m, 1H), 7.09 (dd, *J*_1_ = 7.5 Hz and *J*_2_ = 7.0 Hz, 1H), 6.95 (d, *J* = 8.0 Hz, 1H) 5.45–5.38 (m, 2H), 3.96 (s, 3H), 3.02 (dd, *J*_1_ = 8.5 Hz and *J*_2_ = 9.0 Hz, 2H) 2.41 (s, 3H), 2.16 (dd, *J*_1_ = 7.0 Hz and *J*_2_ = 7.0 Hz, 2H), 1.89 (t, *J* = 6.5 Hz, 2H), 1.70–1.61 (m, 2H), 1.60–1.51 (m, 1H) and 0.83 (d, *J* = 6.5 Hz, 6H) ppm.

^13^C[^1^H] NMR (125 MHz, CDCl_3_) *δ* 142.2, 139.9, 134.6, 129.8, 129.6, 125.3, 124.0, 123.2, 122.7, 122.4, 119.6, 118.9, 117.7, 108.7, 36.6, 32.7, 31.5, 28.8, 28.1, 27.7, 22.6 and 20.2 ppm.

IR (ATR): 2917, 1465, 12463, 1330, 1245, 1096, 826 and 736 cm^−1^.

HRMS (ESI) *m*/*z*: [M + H]^+^ calcd for C_23_H_30_N 320.2373; found 320.2341 (10.0 ppm).

TLC: *R*_f_ = 0.4 (19 : 1 hexane/EtOAc).

#### 2,9-Dimethyl-1-(7-methyloctyl)-9*H*-carbazole (12d)

Following GP-III. To a stirring solution of olefin 11d (48 mg, 0.15 mmol, 1.0 equiv.) in EtOAc (4 mL), Pd/C (5 mg, 10 wt%) was added. The resulting reaction mixture was stirred under a hydrogen (1 atm) atmosphere for 6 h at room temperature. The reaction mixture was filtered and purification of the crude product *via* silica gel column chromatography (19 : 1 hexane/EtOAc) gave the desired hydrogenation product 12d (46 mg, 0.14 mmol, 95.4%) as a colorless gummy compound.

^1^H NMR (500 MHz, CDCl_3_) *δ* 8.01 (d, *J* = 7.5 Hz, 1H), 7.82 (d, *J* = 8.0 Hz, 1H), 7.43 (dd, *J*_1_ = 8.0 Hz and *J*_2_ = 7.5 Hz, 1H), 7.36 (d, *J* = 8.5, 1H), 7.19 (dd, *J*_1_ = 7.0 Hz and *J*_2_ = 7.0 Hz, 1H), 7.05 (d, *J* = 8.0 Hz, 1H), 4.07 (s, 3H), 3.11 (dd, *J*_1_ = 8.0 Hz and *J*_2_ = 8.5 Hz, 2H), 2.5 (s, 3H), 1.72–1.65 (m, 2H), 1.51 (t, *J* = 6.7 Hz, 2H), 1.41–1.36 (m, 2H), 1.36–1.30 (m, 3H), 1.22–1.16 (m, 2H) and 0.86 (d, *J* = 6.5, 6H) ppm.

^13^C[^1^H] NMR (125 MHz, CDCl_3_) *δ* 142.3, 140.0, 134.5, 125.3, 124.3, 123.3, 122.8, 122.5, 119.6, 119.0, 117.6, 108.7, 39.2, 32.7, 31.7, 30.2, 29.9, 28.5, 28.1, 27.5, 22.8 and 20.2 ppm.

IR (ATR): 3413, 2928, 2855, 1648, 1595, 1429, 1413, 1372, 1324, 1256, 1049, 862 and 734 cm^−1^.

HRMS (ESI) *m*/*z*: [M + H]^+^ calcd for C_23_H_32_N 322.2529; found 322.2499 (9.3 ppm).

TLC: *R*_f_ = 0.45 (hexane).

#### 3-Bromo-2,9-dimethyl-1-(7-methyloctyl)-9*H*-carbazole (13d)

Following GP-IV. *N*-Bromo-succinimide (25.5 mg, 0.14 mmol, 1.0 equiv.) was added to a solution of carbazole 12d (44 mg, 0.14 mmol, 1.0 equiv.) in chloroform (CHCl_3_) (6 mL) under a nitrogen atmosphere. The reaction mixture was stirred for 6 min. After completion, water (10 mL) was added and extracted with CH_2_Cl_2_ (3 × 5 mL). The combined organic layers were dried and concentrated *in vacuo*. Purification of the crude product *via* silica gel column chromatography (19 : 1 hexanes/EA) provided the desired product 13d (53 mg, 0.13 mmol, 93%) as a gummy compound.

^1^H NMR (500 MHz, CDCl_3_) *δ* 8.14 (s, 1H), 7.96 (d, *J* = 8.0 Hz, 1H), 7.46 (dd, *J*_1_ = 8.0 Hz and *J*_2_ = 7.0 Hz, 1H), 7.34 (d, *J* = 8.0 Hz, 1H), 7.22 (t, *J* = 7.5 Hz, 1H), 3.9 (s, 3H), 3.11 (dd, *J*_1_ = 8.0 Hz and *J*_2_ = 8.5 Hz, 2H), 2.58 (s, 3H), 1.66 (dd, *J*_1_ = 10.5 Hz and *J*_2_ = 6.0 Hz, 2H), 1.55 (dt, *J*_1_ = 14.9 Hz and *J*_2_ = 7.0 Hz, 3H), 1.40 (dd, *J*_1_ = 9.9 Hz and *J*_2_ = 4.5 Hz, 2H), 1.37–1.33 (m, 2H), 1.26–1.19 (m, 2H) and 0.91 (d, *J* = 6.5 Hz, 6H) ppm.

^13^C[^1^H] NMR (125 MHz, CDCl_3_) *δ* 142.5, 139.1, 132.9, 126.0,126.0, 123.7, 122.1, 121.6, 119.8, 119.3, 116.2, 108.9, 39.2, 32.7, 31.7, 30.0, 29.9, 29.6, 28.1, 27.5, 22.8 and 19.8 ppm.

IR (ATR): 2961, 2856, 2377, 1462, 1402, 1276, 1132, 1012, 819, 774 and 735 cm^−1^.

HRMS (ESI) *m*/*z*: [M + H]^+^ calcd for C_23_H_31_BrN 400.1634; found 400.1636 (0.5 ppm).

TLC: *R*_f_ = 0.5 (hexane).

#### 3-Methoxy-2,9-dimethyl-1-(6-methylheptyl)-9*H*-carbazole (14d)

Following GP-V. A freshly prepared NaOMe solution (∼2.7 M in MeOH) was added to a DMF (1.6 mL) solution of 3-bromocarbazole 13d (40 mg, 0.10 mmol, 1.0 equiv.), followed by CuI (79 mg, 0.40 mmol, 4.0 equiv.), and the reaction mixture stirred at 115 °C for 15 h. The reaction mixture was filtered, and the filtrate was sequentially washed with saturated NH_4_Cl solution (5 mL), water (10 mL) and brine (5 mL), dried and concentrated *in vacuo*. Purification of the crude product *via* silica gel column chromatography (9 : 1 hexanes/EtOAc) provided the desired 3-methoxycarbazole 14d (32 mg, 0.09 mmol, 91%) as a colorless liquid.

^1^H NMR (400 MHz, CDCl_3_) *δ* 8.0 (d, *J* = 7.6 Hz, 1H), 7.44–7.41 (m, 2H), 7.37–7.36 (m, 1H), 7.17 (dd, *J*_1_ = 7.2 Hz and *J*_2_ = 7.6 Hz, 1H), 4.0 (s, 3H), 3.95 (s, 3H), 3.13 (dd, *J*_1_ = 8.0 Hz and *J*_2_ = 8.0 Hz, 2H), 2.83 (s, 3H), 1.68 (q, *J* = 8.2 Hz, 2H), 1.57–1.49 (m, 3H), 1.36 (q, *J* = 8.3 Hz, 4H), 1.21 (t, *J* = 7.1 Hz, 2H) and 0.89 (d, *J* = 6.4, 6H) ppm.

^13^C[^1^H] NMR (100 MHz, CDCl_3_) *δ* 152.2, 142.4, 134.7, 125.8, 125.1, 124.7, 123.2, 121.8, 119.5, 118.4, 108.8, 99.1, 56.2, 39.2, 32.8, 31.7, 30.1, 29.9, 28.8, 28.1, 27.5, 22.8 and 12.1 ppm.

IR (ATR): 2935, 2853, 2384, 1466, 1419, 1293, 1220, 1149, 1129, 844 and 765 cm^−1^.

HRMS (ESI) *m*/*z*: [M + Na]^+^ calcd for C_24_H_33_NNaO 374.2454; found 374.2486 (8.5 ppm).

TLC: *R*_f_ = 0.4 (19 : 1 hexane/EtOAc).

#### 2,9-Dimethyl-1-(7-methyloctyl)-9*H*-carbazol-3-ol (15d)

Following GP-VI. To a stirring solution of 3-methoxycarbazole 14d (28 mg, 0.08 mmol, 1.0 equiv.) in anhydrous CH_2_Cl_2_ (4 mL) at 0 °C, a 1 M solution of boron tribromide (BBr_3_) in CH_2_Cl_2_ (0.33 mL, 0.33 mmol, 4.0 equiv.) was added dropwise, and further stirred for 11 h. The reaction was quenched with H_2_O (5 mL). The organic layer was extracted with CH_2_Cl_2_ (3 × 5 mL). The combined organic layers were dried (Na_2_SO_4_) and concentrated *in vacuo*. Purification of the crude product *via* silica gel column chromatography (4 : 1 hexanes/EtOAc) provided the desired phenol 15d (26.2 mg, 0.07 mmol, 93% yield) as a gummy compound.

^1^H NMR (400 MHz, CDCl_3_) *δ* 7.91 (d, *J* = 7.6 Hz, 1H), 7.42 (t, *J* = 7.6 Hz, 1H), 7.34 (s, 2H), 7.14 (dd, *J*_1_ = 7.2 Hz and *J*_2_ = 7.6 Hz, 1H), 4.03 (s, 3H), 3.11 (dd, *J*_1_ = 8.0 Hz and *J*_2_ = 8.4 Hz, 2H), 2.39 (s, 3H), 1.67 (t, *J* = 7.8 Hz, 2H), 1.56–1.49 (m, 3H), 1.35 (dt, *J*_1_ = 16.1 Hz and *J*_2_ = 7.4 Hz, 4H), 1.22–1.17 (m, 2H) and 0.88 (d, *J* = 6.4, 6H) ppm.

^13^C[^1^H] NMR (100 MHz, CDCl_3_) *δ* 146.6, 141.5, 133.9, 124.6, 124.2, 121.6, 121.3, 121.0, 118.5, 117.3, 107.6, 101.9, 38.0, 31.6, 30.6, 29.0, 28.7, 27.6, 26.9, 26.4, 21.6 and 11.0 ppm.

IR (ATR): 3342, 2934, 2863, 2372, 2351, 1492, 1469, 1297, 1270, 902, 776 and 734 cm^−1^.

HRMS (ESI) *m*/*z*: [M + H]^+^ calcd for C_23_H_32_NO 338.2478; found 338.2485 (2.0 ppm).

TLC: *R*_f_ = 0.3 (9 : 1 hexane/EtOAc).

#### 2,9-Dimethyl-1-(6-methylheptyl)-3*H*-carbazole-3,4(9*H*)-dione-[*N*-methyl carbazoquinocin-F] (6)

Following GP-VII. A solution of carbazol-3-ol 15d (21 mg, 0.06 mmol) and (PhSeO)_2_O (45.0. mg, 0.12 mmol, 2.0 equiv.) in THF (4 mL) was stirred at 50 °C for 30 min. The reaction mixture was quenched with water, and the residual compound from the aqueous layer was extracted with EtOAc (3 × 4 mL). The combined organic layers were dried (Na_2_SO_4_) and concentrated *in vacuo*. Purification of the crude product *via* silica gel column chromatography (3 : 1 hexanes/EtOAc) provided the desired *N*-methyl carbazoquinocin F 6 (20.6 mg, 0.06 mmol, 94% yield) as a dark-brown glittering solid.

^1^H NMR (400 MHz, CDCl_3_) *δ* 8.06–8.05 (m, 1H), 7.18 (s, 3H), 3.82 (s, 3H), 2.62 (dd, *J*_1_ = 6.8 Hz and *J*_2_ = 8.0 Hz, 2H), 1.84 (s, 3H), 1.54–1.48 (m, 2H), 1.47–1.38 (m, 3H), 1.27 (s, 4H), 1.14–1.07 (m, 2H) and 0.80 (d, *J* = 6.4 Hz, 6H) ppm.

^13^C[^1^H] NMR (100 MHz, CDCl_3_) *δ* 183.3, 173.6, 144.9, 142.7, 139.6, 134.5, 125.8, 124.9, 121.9, 113.8, 110.8, 77.5, 77.2, 76.8, 39.0, 33.1, 30.0, 29.9, 29.7, 28.4, 28.3, 28.1, 27.4, 22.8 and 11.9 ppm.

IR (ATR): 2961, 2934, 2846, 1661, 1655, 1642, 1489, 1472, 1424, 1368, 1262 and 742 cm^−1^.

HRMS (ESI) *m*/*z*: [M + H]^+^ calcd for C_23_H_30_NO_2_ 352.2271; found 352.2248 (6.5 ppm).

TLC: *R*_f_ = 0.4 (4 : 1 hexane/EtOAc).

M.P.: 169–171 °C.

#### Ethyl 4-(2,9-dimethyl-9*H*-carbazol-1-yl)-2-methylbutanoate (17)

Following the reported procedure,^10^ under an N_2_ atmosphere, a flame-dried round-bottom flask was charged with a solution of ethyl 4-(2,9-dimethyl-9*H*-carbazol-1-yl)butanoate 16 (50 mg, 0.16 mmol, 1.0 equiv.) in anhydrous THF (5 mL) and LDA (37 mg, 0.32 mmol, 2.0 equiv.) was added at 0 °C portion-wise and stirred for 30 min. This was followed by the dropwise addition of methyl iodide (46 mg, 0.32 mmol, 2.0 equiv.) at 0 °C, and then the reaction was stirred for 6 h at room temperature until TLC showed the complete consumption of the ester. After completion, a saturated NH_4_Cl solution (6 mL) was added to quench the reaction at 0 °C. Then ethyl acetate (5 mL) and water (5 mL) were added. After separation of the layers, the residual compound from the aqueous layer was extracted with EtOAc (3 × 5 mL). The combined organic layers were dried over anhydrous Na_2_SO_4_, filtered, and concentrated *in vacuo*. Purification of the crude product *via* silica gel column chromatography (hexane/EA) provided the corresponding ethyl 4-(2,9-dimethyl-9*H*-carbazol-1-yl)-2-methylbutanoate 17 (31 mg, 0.09 mmol, 59% yield) as a pale-yellow color liquid.

^1^H NMR (400 MHz, CDCl_3_) *δ* 7.94 (d, *J* = 7.7 Hz, 1H), 7.77 (d, *J* = 7.8 Hz, 1H), 7.36 (t, *J* = 7.6 Hz, 1H), 7.29 (d, *J* = 8.2 Hz, 1H), 7.12 (t, *J* = 7.4 Hz, 1H), 6.98 (d, *J* = 7.8 Hz, 1H), 4.13 (q, *J* = 7.1 Hz, 2H), 4.00 (s, 3H), 3.16–2.97 (m, 2H), 2.59 (dd, *J*_1_ = 13.4 Hz and *J*_2_ = 7.1 Hz, 1H), 2.43 (s, 3H), 1.96–1.94 (m, 1H), 1.76–1.66 (m, 1H) and 1.28–1.18 (m, 6H) ppm.

^13^C[^1^H] NMR (100 MHz, CDCl_3_) *δ* 176.5, 142.2, 139.9, 134.7, 125.4, 123.1, 123.1, 122.8, 122.5, 119.7, 119.0, 117.9, 108.7, 60.6, 40.2, 35.3, 32.7, 26.2, 20.1, 17.5 and 14.5 ppm.

IR (ATR): 2944, 1738, 1580, 1453, 1218, 1199, 1153, 1127, 1026, 925, 827 and 749 cm^−1^.

HRMS (ESI) *m*/*z*: [M + H]^+^ calcd for C_23_H_32_N 324.1958; found 324.1988 (9.2 ppm).

TLC: *R*_f_ = 0.6 (9 : 1 hexane/EtOAc).

#### 4-(2,9-Dimethyl-9*H*-carbazol-1-yl)-2-methylbutan-1-ol (18)

Under an N_2_ atmosphere, a flame-dried round-bottom flask was charged with a solution of 4-(2,9-dimethyl-9*H*-carbazol-1-yl)-2 methylbutanoate 17 (62 mg, 0.19 mmol, 1.0 equiv.) in anhydrous THF (5 mL) and LAH (15 mg, 0.38 mmol, 2.0 equiv.) was added at 0 °C portion-wise and stirred for 4 h at room temperature until TLC showed the complete consumption of the ester. After completion, a saturated NH_4_Cl solution (6 mL) was added to quench the reaction at 0 °C. EtOAc (5 mL) and water (5 mL) were added. After separation of the layers, the residual compound from the aqueous layer was extracted with EtOAc (3 × 5 mL). The combined organic layers were dried over anhydrous Na_2_SO_4_, filtered, and concentrated *in vacuo*. Purification of the crude product *via* silica gel column chromatography (hexanes/EA) provided the corresponding ethyl 4-(2,9-dimethyl-9*H*-carbazol-1-yl)-2-methylbutan-1-ol 18 (50 mg, 0.18 mmol, 93% yield) as a colourless gummy compound.

^1^H NMR (500 MHz, CDCl_3_) *δ* 7.92 (d, *J* = 7.7 Hz, 1H), 7.74 (d, *J* = 7.9 Hz, 1H), 7.34 (t, *J* = 7.8 Hz, 1H), 7.25 (d, *J* = 8.3 Hz, 1H), 7.11 (d, *J* = 7.5 Hz, 1H), 6.96 (d, *J* = 7.9 Hz, 1H), 3.95 (s, 3H), 3.53–3.41 (m, 2H), 3.12–2.94 (m, 2H), 2.41 (s, 3H), 1.76–1.56 (m, 2H), 1.52 (s, 1H), 1.42 (dt, *J*_1_ = 13.3 Hz and *J*_2_ = 5.8 Hz, 1H) and 1.00 (d, *J* = 6.6 Hz, 3H) ppm.

^13^C[^1^H] NMR (125 MHz, CDCl_3_) *δ* 142.2, 139.9, 134.4, 125.3, 123.9, 123.1, 122.8, 122.5, 119.6, 118.9, 117.7, 108.7, 68.0, 36.5, 34.9, 32.6, 26.0 and 20.1, 16.7 ppm.

IR (ATR): 3360, 2944, 1738, 1580, 1453, 1218, 1199, 1153, 1127, 1026, 925, 827 and 749 cm^−1^.

HRMS (ESI) *m*/*z*: [M + H]^+^ calcd for C_19_H_23_NO 281.1780; found 281.1792 (4.2 ppm).

TLC: *R*_f_ = 0.2 (9 : 1 hexane/EtOAc).

#### 4-(2,9-Dimethyl-9*H*-carbazol-1-yl)-2-methylbutanal (19)

Under an N_2_ atmosphere, a flame-dried round bottom-flask was charged with a solution of ethyl 4-(2,9-dimethyl-9*H*-carbazol-1-yl)-2-methylbutan-1-ol 18 (42 mg, 0.14 mmol, 1.0 equiv.) in anhydrous dichloromethane (5 mL) and Dess–Martin periodate (82 mg, 0.19 mmol, 1.3 equiv.) was added at 0 °C portion-wise and stirred for 6 h at room temperature until TLC showed the complete consumption of the alcohol. After completion, the reaction was filtered and washed with dichloromethane and dried over anhydrous Na_2_SO_4_ and concentrated *in vacuo*. Purification of the crude product *via* silica gel column chromatography (hexanes/EtOAc) provided the corresponding ethyl 4-(2,9-dimethyl-9*H*-carbazol-1-yl)-2-methylbutanal 19 (38 mg, 0.13 mmol, 91% yield) as a colorless liquid.

^1^H NMR (400 MHz, CDCl_3_) *δ* 9.57 (s, 1H), 7.89 (d, *J* = 7.6 Hz, 1H), 7.72 (d, *J* = 7.8 Hz, 1H), 7.32 (t, *J* = 7.5 Hz, 1H), 7.21 (d, *J* = 8.2 Hz, 1H), 7.09 (t, *J* = 7.2 Hz, 1H), 6.93 (d, *J* = 7.8 Hz, 1H), 3.87 (s, 3H), 3.07–2.90 (m, 2H), 2.37 (s, 4H), 1.88 (dd, *J*_1_ = 13.1 Hz and *J*_2_ = 5.6 Hz, 1H), 1.64–1.50 (m, 1H) and 1.12 (d, *J* = 7.1 Hz, 3H) ppm.

^13^C[^1^H] NMR (100 MHz, CDCl_3_) *δ* 204.4, 142.2, 139.8, 134.5, 125.4, 123.0, 122.8, 122.5, 119.6, 119.0, 117.9, 108.7, 46.7, 32.6, 31.9, 25.7, 20.1 and 13.6 ppm.

IR (ATR): 2976, 2941, 1725, 1673, 1622, 1468, 1444, 1433, 1266, 842, 823 and 746 cm^−1^.

HRMS (ESI) *m*/*z*: [M + H]^+^ calcd for C_19_H_22_NO 380.1696; found 380.1725 (8.0 ppm).

TLC: *R*_f_ = 0.4 (19 : 1 hexane/EtOAc).

#### 2,9-Dimethyl-1-(3-methylpent-4-en-1-yl)-9*H*-carbazole (20)

Following GP-II. ^*t*^BuOK (37 mg, 0.30 mmol, 1.8 equiv.) was added to a solution of methyl(triphenyl phosphonium) bromide (120 mg, 0.33 mmol, 2.0 equiv.) in anhydrous THF (5 mL), followed by aldehyde 19 (50 mg, 0.16 mmol, 1.0 equiv.) and stirred for 12 h. The corresponding desired alkene product 20 was isolated as a colorless gummy compound (39 mg, 0.14 mmol, 84%) using hexane/EtOAc (9 : 1) as the eluent.

^1^H NMR (400 MHz, CDCl_3_) *δ* 7.88 (d, *J* = 7.6 Hz, 1H), 7.70 (d, *J* = 7.8 Hz, 1H), 7.30 (t, *J* = 7.1 Hz, 1H), 7.19 (d, *J* = 8.2 Hz, 1H), 7.11–7.03 (m, 1H), 6.92 (d, *J* = 7.6 Hz, 1H), 5.71 (dt, *J*_1_ = 17.0 Hz and *J*_2_ = 8.5 Hz, 1H), 4.96–4.62 (m, 2H), 3.86 (s, 3H), 3.04–2.82 (m, 2H), 2.36 (s, 3H), 2.29–2.16 (m, 1H), 1.53 (d, *J* = 6.6 Hz, 2H) and 1.01 (dd, *J*_1_ = 3.7 Hz and *J*_2_ = 2.9 Hz, 3H) ppm.

^13^C[^1^H] NMR (100 MHz, CDCl_3_) *δ* 144.0, 142.2, 139.9, 134.5, 125.3, 123.9, 123.1, 122.7, 122.5, 119.6, 118.9, 117.6, 113.6, 108.6, 38.7, 38.2, 32.7, 26.2, 20.3 and 20.2 ppm.

IR (ATR): 2917, 1496, 1475, 1325, 1308, 1235, 842 and 738 cm^−1^.

HRMS (ESI) *m*/*z*: [M + H]^+^ calcd for C_20_H_26_N 278.1903; found 278.1894 (3.2 ppm).

TLC: *R*_f_ = 0.4 (19 : 1 hexane/EtOAc).

#### 2,9-Dimethyl-1-(3-methylpentyl)-9*H*-carbazole (21)

Following GP-III. Pd/C (4.4 mg, 10 wt%) was added to a stirred solution of olefin 20 (44 mg, 0.16 mmol, 1.0 equiv.) in EtOAc (4 mL). The resulting reaction mixture was stirred under a hydrogen (1 atm) atmosphere for 3 h at room temperature. The reaction mixture was filtered and purification of the crude product *via* silica gel column chromatography (19 : 1 hexanes/EtOAc) provided the desired hydrogenation product 21 (44.5 mg, 0.16 mmol, 96%) as a colorless liquid.

^1^H NMR (400 MHz, CDCl_3_) *δ* 7.90 (d, *J* = 7.4 Hz, 1H), 7.72 (dd, *J*_1_ = 7.8 Hz and *J*_2_ = 1.6 Hz, 1H), 7.31 (t, *J* = 7.6 Hz, 1H), 7.22 (d, *J* = 8.2 Hz, 1H), 7.08 (t, *J* = 6.4 Hz, 1H), 6.94 (d, *J* = 7.7 Hz, 1H), 3.91 (s, 3H), 3.08–2.87 (m, 2H), 2.39 (s, 3H), 1.64–1.53 (m, 1H), 1.51–1.31 (m, 3H), 1.23–1.11 (m, 1H), 0.94 (dd, *J*_1_ = 6.3 Hz and *J*_2_ = 1.6 Hz, 3H) and 0.84–0.81 (m, 3H) ppm.

^13^C[^1^H] NMR (100 MHz, CDCl_3_) *δ* 142.2, 139.9, 134.4, 125.2, 124.4, 123.2, 122.7, 122.5, 119.6, 118.9, 117.5, 108.6, 38.5, 35.5, 32.6, 29.6, 26.3, 20.1, 19.3 and 11.7 ppm.

IR (ATR): 3423, 2932, 2889, 1651, 1532, 1449, 1431, 1372, 1334, 1243, 1089, 834 and 748 cm^−1^.

HRMS (ESI) *m*/*z*: [M + H]^+^ calcd for C_20_H_26_N 280.2060; found 280.2063 (1.0 ppm).

TLC: *R*_f_ = 0.4 (hexane).

#### 3-Bromo-2,9-dimethyl-1-(3-methylpentyl)-9*H*-carbazole (22)

Following GP-IV. *N*-Bromo-succinimide (25.4 mg, 0.14 mmol, 1.0 equiv.) was added a solution of carbazole 21 (40 mg, 0.14 mmol, 1.0 equiv.) in chloroform (CHCl_3_) (6 mL) under a nitrogen atmosphere. The reaction mixture was stirred for 6 min. After completion, water (10 mL) was added and extracted with CH_2_Cl_2_ (3 × 5 mL). The combined organic layers were dried and concentrated *in vacuo*. Purification of the crude product *via* silica gel column chromatography (19 : 1 hexanes/EtOAc) provided the corresponding 3-bromocarbazole 22 (45 mg, 0.12 mmol, 88%) as a colorless liquid.

^1^H NMR (400 MHz, CDCl_3_) *δ* 7.99 (s, 1H), 7.82 (s, 1H), 7.31 (t, *J* = 7.6 Hz, 1H), 7.17 (d, *J* = 8.2 Hz, 1H), 7.07 (t, *J* = 7.4 Hz, 1H), 3.80 (s, 3H), 3.03–2.83 (m, 2H), 2.42 (s, 3H), 1.58–1.47 (m, 1H), 1.43 (dd, *J*_1_ = 12.2 Hz and *J*_2_ = 6.0 Hz, 1H), 1.37–1.27 (m, 2H), 1.15 (dt, *J*_1_ = 14.2 Hz and *J*_2_ = 7.3 Hz, 1H), 0.92 (d, *J* = 6.4 Hz, 3H) and 0.83 (t, *J* = 7.3 Hz, 3H) ppm.

^13^C[^1^H] NMR (100 MHz, CDCl_3_) *δ* 142.4, 139.0, 132.8, 126.0, 126.0, 123.6, 122.1, 121.5, 119.8, 119.2, 116.8, 108.8, 38.4, 35.5, 32.7, 29.6, 27.3, 19.7, 19.2 and 11.7 ppm.

IR (ATR): 2969, 2869, 2374, 1463, 1416, 1291, 1186, 1055, 832, 768 and 749 cm^−1^.

HRMS (ESI) *m*/*z*: [M + H]^+^ calcd for C_20_H_25_NBr 358.1165; found 358.1159 (2.0 ppm).

TLC: *R*_f_ = 0.5 (hexane).

#### 3-Methoxy-2,9-dimethyl-1-(3-methylpentyl)-9*H*-carbazole (23)

Following GP-V. A freshly prepared NaOMe solution (∼2.7 M in MeOH) was added to a DMF (1.6 mL) solution of 3-bromocarbazole 22 (40 mg, 0.11 mmol, 1.0 equiv.), followed by CuI (93 mg, 0.46 mmol, 4.0 equiv.), and the reaction mixture stirred at 115 °C for 15 h. The reaction mixture was filtered, and the filtrate was sequentially washed with saturated NH_4_Cl solution (5 mL), water (10 mL) and brine (5 mL), dried and concentrated *in vacuo*. Purification of the crude product *via* silica gel column chromatography (9 : 1 hexanes/EtOAc) provided the desired 3-methoxycarbazole 23 (29.6 mg, 0.09 mmol, 86%) as a pale-yellow liquid.

^1^H NMR (400 MHz, CDCl_3_) *δ* 7.90 (d, *J* = 7.7 Hz, 1H), 7.32 (t, *J* = 7.4 Hz, 2H), 7.25 (d, *J* = 8.2 Hz, 1H), 7.09 (d, *J* = 7.3 Hz, 1H), 3.95 (s, 3H), 3.85 (s, 3H), 3.14–2.93 (m, 2H), 2.28 (s, 3H), 1.66–1.56 (m, 1H), 1.53–1.44 (m, 2H), 1.42–1.37 (m, 1H), 1.20 (dd, *J*_1_ = 13.7 Hz and *J*_2_ = 6.7 Hz, 1H), 0.97 (d, *J* = 6.4 Hz, 3H) and 0.86 (t, *J* = 7.3 Hz, 3H) ppm.

^13^C[^1^H] NMR (100 MHz, CDCl_3_) *δ* 152.3, 142.4, 134.8, 125.9, 125.1, 124.6, 123.2, 121.8, 119.5, 118.4, 108.8, 99.1, 56.2, 38.4, 35.6, 32.8, 29.7, 26.6, 19.3, 11.9, and 11.7 ppm.

IR (ATR): 2935, 2862, 2388, 1476, 1413, 1278, 1229, 1164, 1122, 861 and 736 cm^−1^.

HRMS (ESI) *m*/*z*: [M + H]^+^ calcd for C_21_H_28_NO 310.2165; found 310.2143 (7.0 ppm).

TLC: *R*_f_ = 0.2 (hexane).

#### 2,9-Dimethyl-1-(3-methylpentyl)-9*H*-carbazol-3-ol (24)

Following GP-VI. To a stirring solution of 3-methoxycarbazole 23 (27 mg, 0.09 mmol, 1.0 equiv.) in anhydrous CH_2_Cl_2_ (4 mL) at 0 °C, a 1 M solution of boron tribromide (BBr_3_, in CH_2_Cl_2_; 0.35 mL, 0.34 mmol, 4.0 equiv.) was added and further stirred for 11 h. The reaction was quenched with H_2_O (5 mL) and extracted with CH_2_Cl_2_ (3 × 5 mL). The combined organic layers were dried (Na_2_SO_4_) and concentrated *in vacuo*. Purification of the crude product using silica gel column chromatography (4 : 1 hexanes/EtOAc) provided phenol 24 (18 mg, 0.06 mmol, 70% yield) as a light-yellow gummy compound.

^1^H NMR (500 MHz, CDCl_3_) *δ* 7.92 (d, *J* = 7.6 Hz, 1H), 7.42 (t, *J* = 7.9 Hz, 1H), 7.39–7.31 (m, 2H), 7.16 (d, *J* = 6.5 Hz, 1H), 4.69 (s, 1H), 4.04 (s, 3H), 3.13–3.01 (m, 2H), 2.40 (s, 3H), 1.69 (dd, *J*_1_ = 10.7 Hz and *J*_2_ = 5.6 Hz, 1H), 1.63–1.56 (m, 1H), 1.55–1.43 (m, 2H), 1.33–1.25 (m, 1H), 1.07 (dd, *J*_1_ = 6.7 Hz and *J*_2_ = 2.7 Hz, 3H) and 0.96 (t, *J* = 7.4 Hz, 3H) ppm.

^13^C[^1^H] NMR (125 MHz, CDCl_3_) *δ* 147.8, 142.7, 135.1, 125.8, 125.4, 122.8, 122.4, 122.2, 119.7, 118.4, 108.7, 103.1, 38.5, 35.5, 32.8, 29.7, 26.6, 19.3, 12.0 and 11.7 ppm.

IR (ATR): 3325, 2933, 2854, 2362, 2346, 1485, 1448, 1272, 1236, 921, 776 and 731 cm^−1^.

HRMS (ESI) *m*/*z*: [M + NH_4_]^+^ calcd for C_20_H_29_N2O 313.2274; found 313.2246 (9.0 ppm).

TLC: *R*_f_ = 0.3 (9 : 1 hexane/EtOAc).

#### 2,9-Dimethyl-1-(3-methylpentyl)-3*H*-carbazole-3,4(9*H*)-dione[*N*-methylcarbazoquinocin A] (7)

Following GP-VII. A solution of carbazol-3-ol 24 (15 mg, 0.05 mmol) and (PhSeO)_2_O (36.5. mg, 0.10 mmol, 2.0 equiv.) in THF (4 mL) was stirred at 50 °C for 30 min. The mixture was quenched with water, and the residual compound from the aqueous layer was extracted with EtOAc (3 × 5 mL). The combined organic layers were dried (Na_2_SO_4_) and concentrated *in vacuo*. Purification of the crude product using silica gel column chromatography (3 : 1 hexanes/EtOAc) provided the desired *N*-methyl carbazoquinocin A 7 (14 mg, 0.04 mmol 90% yield) as a dark-brown glittering solid.

^1^H NMR (400 MHz, CDCl_3_) *δ* 8.13–8.11 (m, 1H), 7.25 (s, 3H), 3.88 (s, 3H), 2.81–2.57 (m, 2H), 1.90 (s, 3H), 1.50 (td, *J*_1_ = 13.2 Hz and *J*_2_ = 6.5 Hz, 2H), 1.43 (d, *J* = 5.2 Hz, 1H), 1.29–1.23 (m, 2H), 1.02 (d, *J* = 6.2 Hz, 3H) and 0.94 (t, *J* = 7.3 Hz, 3H) ppm.

^13^C[^1^H] NMR (100 MHz, CDCl_3_) *δ* 183.2, 173.7, 144.9, 143.0, 139.6, 134.5, 125.8, 124.9, 121.9, 113.8, 110.8, 35.4, 35.0, 33.1, 29.5, 27.9, 19.0, 11.8 and 11.6 ppm.

IR (ATR): 2966, 2957, 2853, 1662, 1658, 1636, 1492, 1471, 1454, 1379, 1235 and 776 cm^−1^.

HRMS (ESI) *m*/*z*: [M + H]^+^ calcd for C_20_H_24_NO_2_ 310.1802; found 310.1807 (2.0 ppm).

TLC: *R*_f_ = 0.35 (4 : 1 hexane/EtOAc).

M.P.: 149–151 °C.

#### Synthesis of isopropyltriphenylphosphonium bromide (10a)

Following GP-VIII. A solution of triphenylphosphine (1.0 g, 3.7 mmol) and isopropyl bromide (0.5 g, 4.12 mmol) was stirred at 110 °C for 48 h. The reaction was filtered and washed with ether to afford 1.1 g (76%) of product 10a as a white solid.

#### Synthesis of *sec*-butyltriphenylphosphonium bromide (10b)

Following GP-VIII. A solution of triphenylphosphine (1.1 g, 4.19 mmol) and *sec*-butyl bromide (0.63 g, 4.61 mmol) was stirred at 110 °C for 48 h. The reaction was filtered and washed with ether to afford 540 mg (33%) of product 10b as an off-white solid.

#### Synthesis of isobutyltriphenylphosphonium bromide (10c)

Following GP-VIII. A solution of triphenylphosphine (1.1 g, 4.19 mmol) and 1-bromo-2-methylpropane (0.63 g, 4.61 mmol) was stirred at 110 °C for 48 h. The reaction was filtered and washed with ether to afford 1.2 g (72%) of product 10c as a white solid.

#### Synthesis of isopentyltriphenylphosphonium bromide (10d)

Following GP-VIII. A solution of triphenylphosphine (1.1 g, 4.19 mmol) and 1-bromo-3-methylbutane (0.69 g, 4.61 mmol) was stirred at 110 °C for 48 h. The reaction was filtered and washed with ether to afford 1.5 g (86%) of product 10d as a white solid.

#### Synthesis of methyltriphenylphosphonium bromide

Following GP-VIII. A solution of triphenylphosphine (1.1 g, 4.19 mmol) and methyl bromide (0.65 g, 4.61 mmol) was stirred at 110 °C for 48 h. The reaction was filtered and washed with ether to afford 1.4 g (93%) of the product as a white solid.

## Abbreviations

Pd/C: Palladium on carbon
wt%: Weight%
atm: Atmosphere
NBS: *N*-Bromosuccinimide
DIBAL-H: Diisobutylaluminium hydride
*n*-BuLi: *n*-Butyllithium
equiv.: Equivalents
Cat.: Catalytic amount
M: Molar
anhyd.: Anhydrous
mL: Microliter
rt: Room temperature
TLC: Thin layer chromatography
*R*_f_
: Retardation or retention factor
NaHCO_3_: Sodium bicarbonate
Na_2_SO_4_: Sodium sulfate
dr: Diastereomeric ratio
NMR: Nuclear magnetic resonance spectroscopy
IR: Infrared spectroscopy
ATR: Attenuated total reflection
HRMS (ESI): High-resolution electrospray ionization mass spectrometry
Calcd: Calculated
M.P.: Melting point
ppm: Parts per million
^*n*^Bu: *n*-Butyl group
atm: Atmosphere

## Data availability

The data underlying this study are available in the published article and its ESI.†

## Conflicts of interest

The authors declare no competing financial interest.

## Supplementary Material

RA-014-D4RA05347H-s001
