# Supplementary material for: Integrated synthesis of 3,4-carbazoquinone alkaloids N-Me-carbazoquinocin A, B and D–F
Source: RSC Adv. 2024 Aug 28;14(37):27372–84. doi: 10.1039/d4ra05347h (PMC11350554; doi:10.1039/d4ra05347h)

Supplementary Information (SI) for RSC Advances.
This journal is © The Royal Society of Chemistry 2024

Supporting Information for

Integrated Syntheses of 3,4-Carbazoquinone Alkaloids *N*-Me-Carbazoquinocin A-B & D-F

Basavaiah Bommanaboina, and Debayan Roy, Beeraiah Baire*

Indian Institute of Technology Madras, Chennai-600036, Tamil Nadu, India.

*Correspondence to: beeru@iitm.ac.in

Compound S1 S3

Copy of ^{1}H and ^{13}C NMR spectra of all new compounds. S4-38

4-(2,9-Dimethyl-9H-carbazol-1-yl)butan-1-ol (S_1)

Under N_2 atmosphere, a flame dried round bottom flask was charged with the solution of ethyl 4-(2,9-dimethyl-9H-carbazol-1-yl) butanoate (62 mg, 0.1919 mmol, 1.0 equiv.) in anhydrous THF (5 ml) was added LAH (15 mg, 0.3839 mmol, 2.0 equiv.), at 0 °C portion wise and stirred for 3 h at room temperature until the TLC showed complete consumption of the ester. After completion, a saturated NH_4Cl solution (6 mL) was added to quench the reaction at 0 °C. ethyl acetate (5 mL) and water (5 mL) were added. After separation of the layers the residual compound from aqueous layer was extracted with EtOAc (3 x 5 mL). The combined organic layers were dried over anhydrous Na_2SO_4, filtered, and concentrated *in-vacuo*. Purification of crude product via a silica gel column chromatography (Hexanes/EA) provided the corresponding ethyl 4-(2,9-dimethyl-9H-carbazol-1-yl) butan-1-ol **(S_1) (**50 mg, 0.18 mmol, 93% yield) as a white colour oil.

^{1}H NMR (400 MHz, $CDCl_3$): δ 7.96 (d, *J* = 7.7 Hz, 1H), 7.77 (d, *J* = 7.8 Hz, 1H), 7.41–7.33 (m, 1H), 7.26 (d, *J* = 8.3 Hz, 1H), 7.15 (t, *J* = 7.4 Hz, 1H), 6.99 (d, *J* = 7.8 Hz, 1H), 3.90 (s, 3H), 3.58 (t, *J* = 5.9 Hz, 2H), 3.01 (t, *J* = 7.6 Hz, 2H), 2.44 (s, 3H), 1.83 (s, 1H) and 1.71–1.55 (m, 4H) ppm.

^{13}C[^{1}H] NMR (100 MHz, $CDCl_3$): δ 142.1, 139.8, 134.4, 125.2, 123.7, 123.0, 122.7, 122.4, 119.5, 118.9, 117.6, 108.6, 62.5, 32.7, 32.4, 27.9, 27.7, 20.2 ppm.

IR (ATR): 3560, 3341, 2944, 1738, 1580, 1453, 1218, 1199, 1153, 1127, 1026, 925, 827 and 749 cm^{-1}.

HRMS (ESI) m/z: $[M+H]^+$ Calcd. for $C_{18}H_{22}NO$ 268.1696; found 268.1720.

TLC: R_f = R_f = 0.2 (9:1, Hex/EtOAc).

Copy of ^{1}H and ^{13}C NMR spectra of all new compounds

^{1}H NMR (400 MHz, $CDCl_3$) and **^{13}C[^{1}H] NMR** (100 MHz, $CDCl_3$) of **(11a)**

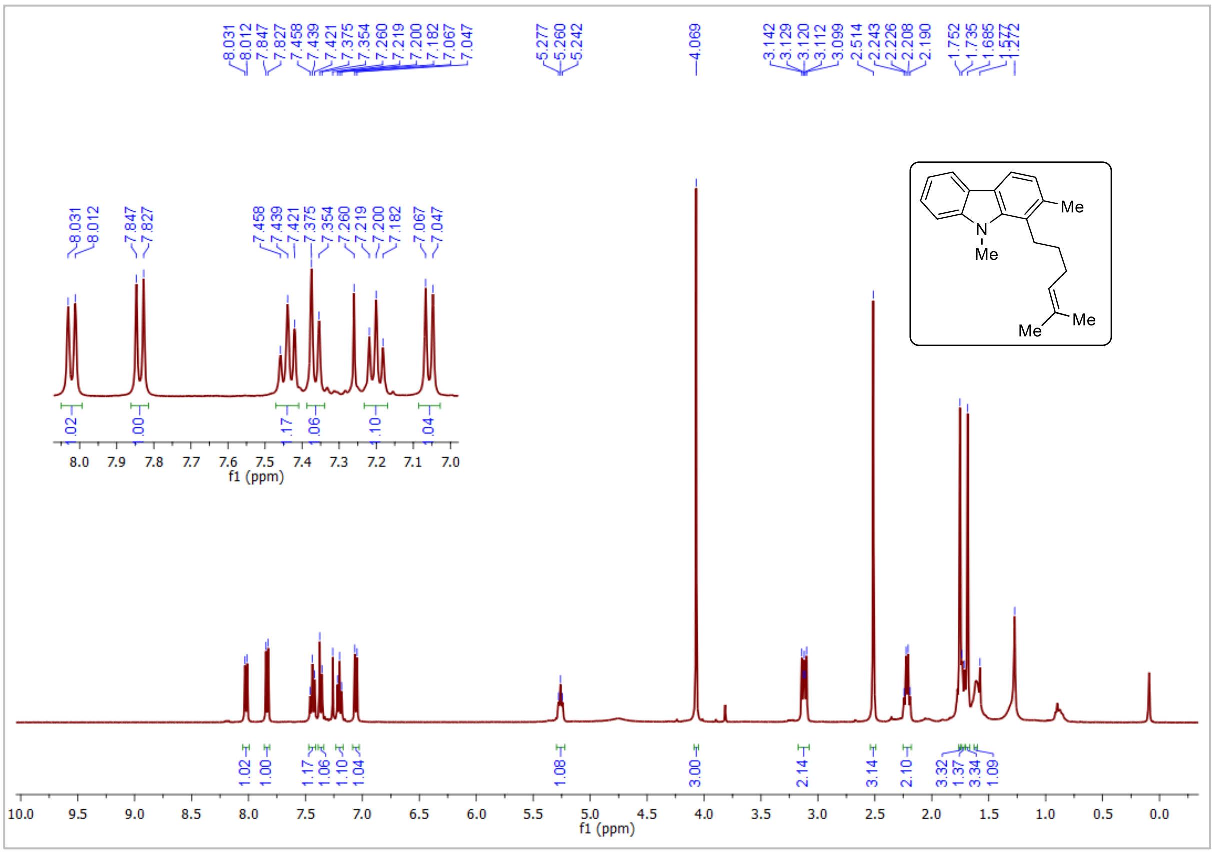

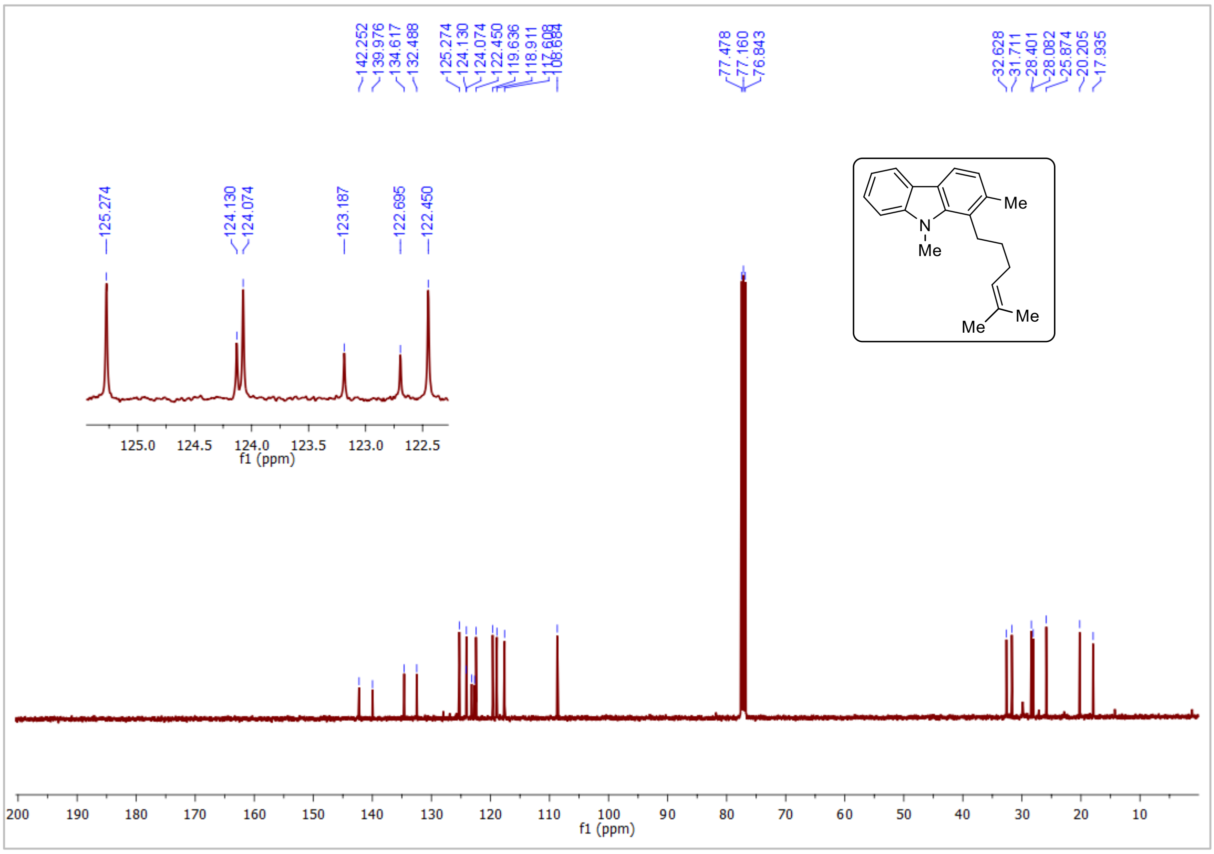

^{1}H NMR (500 MHz, $CDCl_3$) and ^{13}C[^{1}H] NMR (125 MHz, $CDCl_3$) of **(12a)**

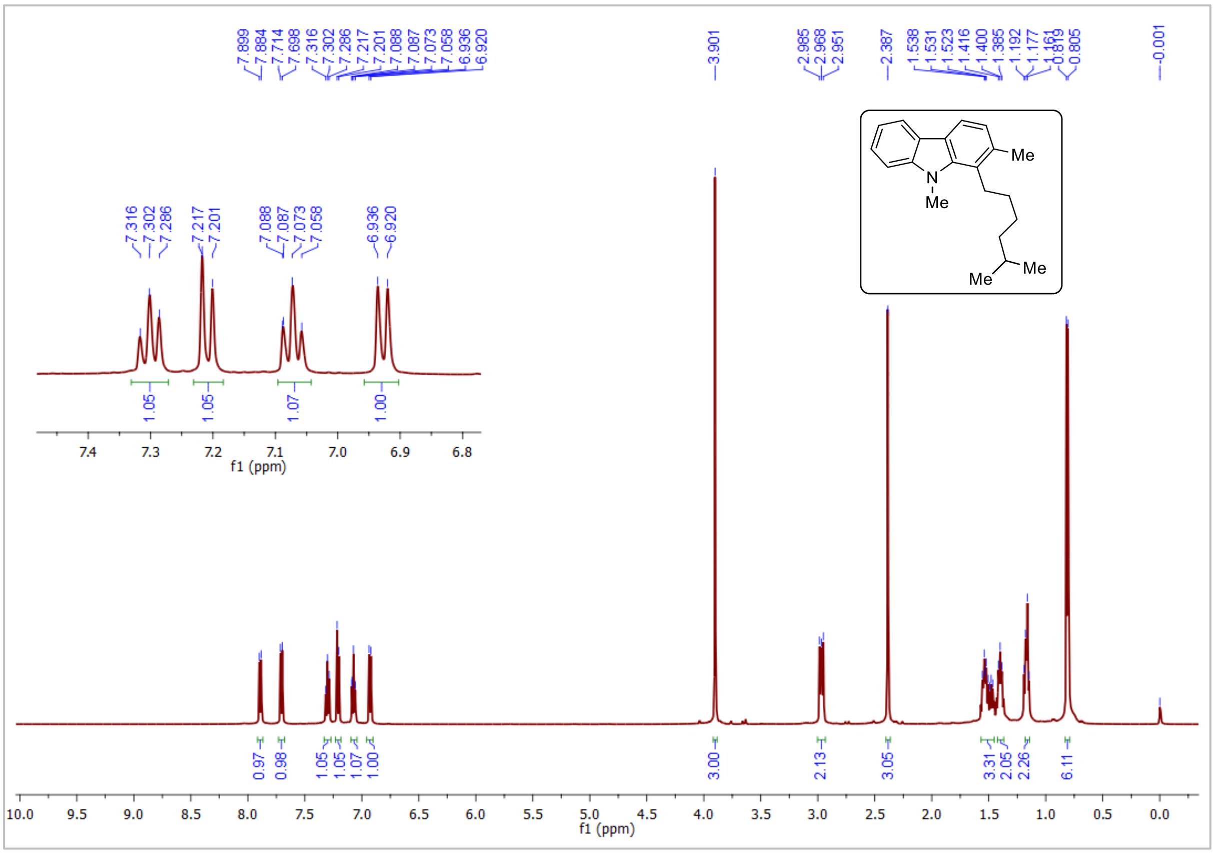

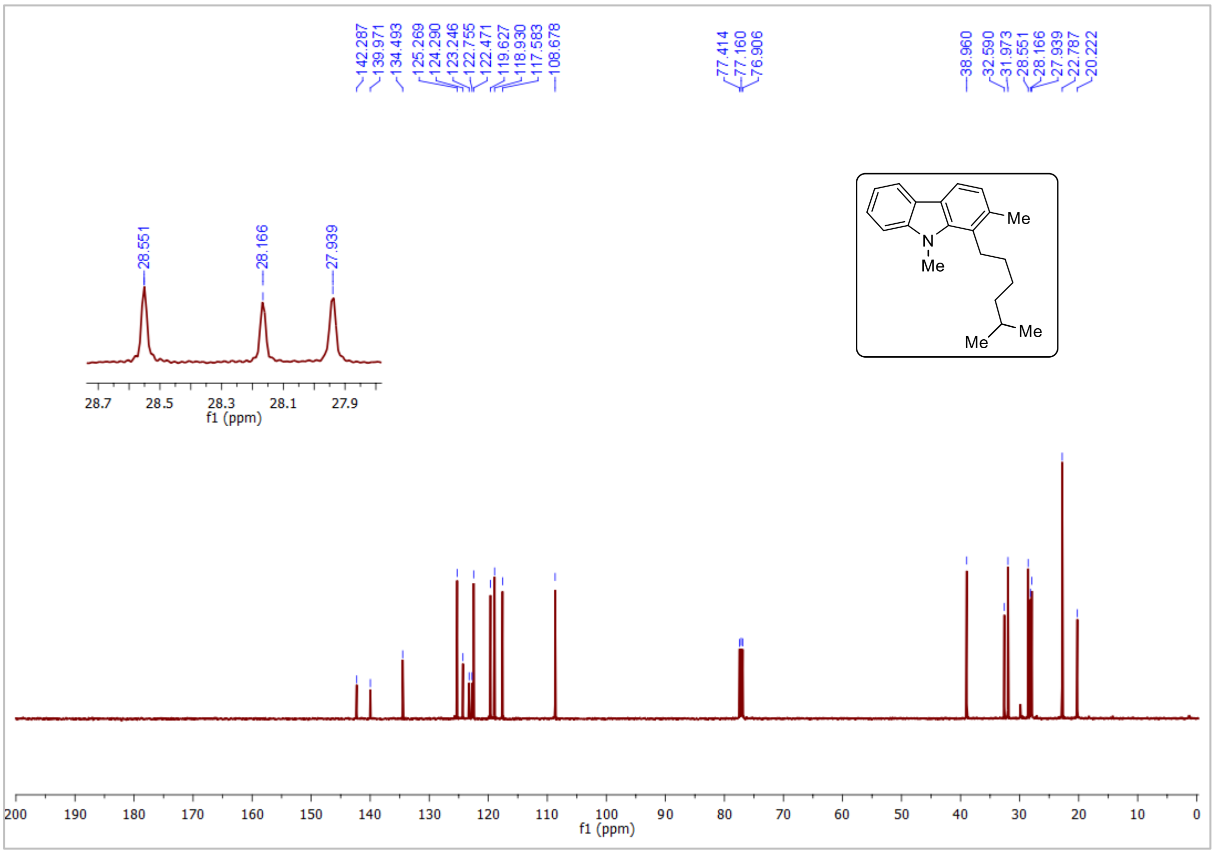

^{1}H NMR (400 MHz, $CDCl_3$) and **^{13}C[^{1}H] NMR** (100 MHz, $CDCl_3$) of **(13a)**

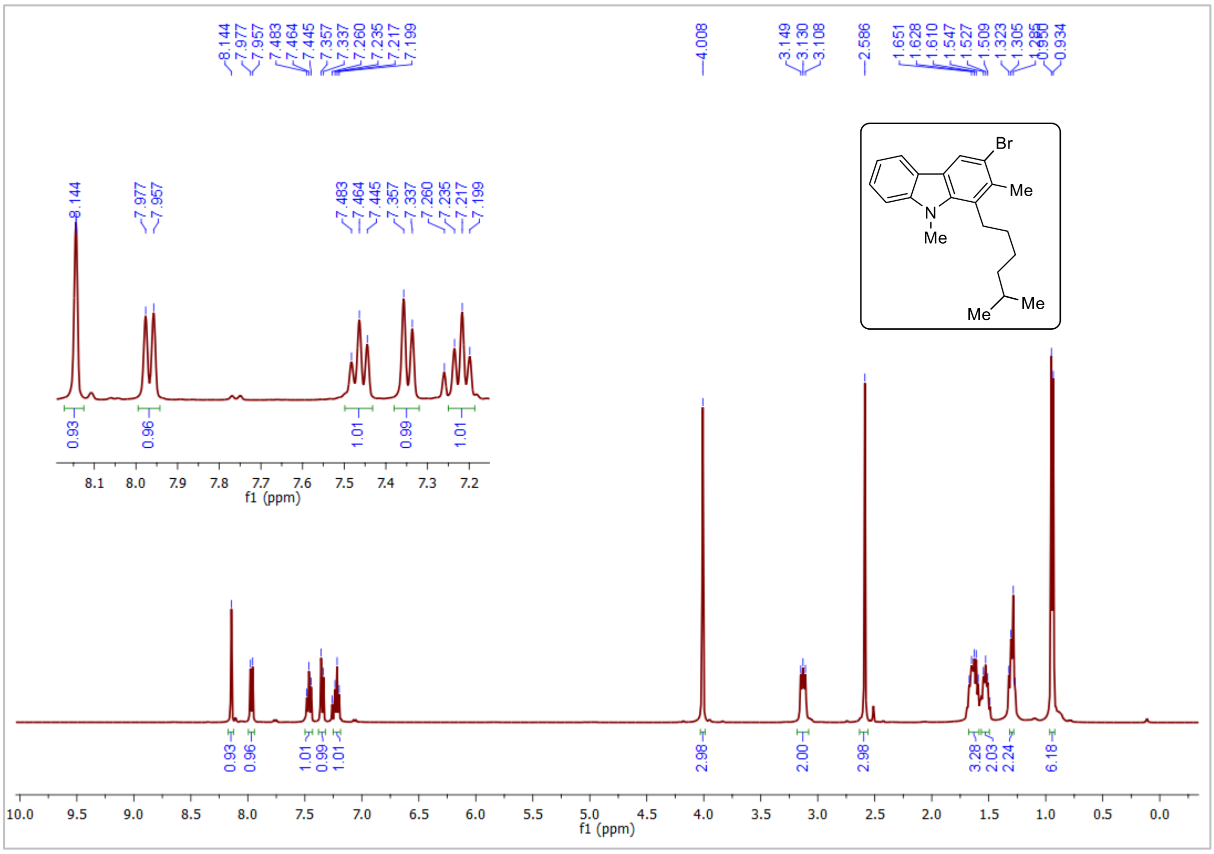

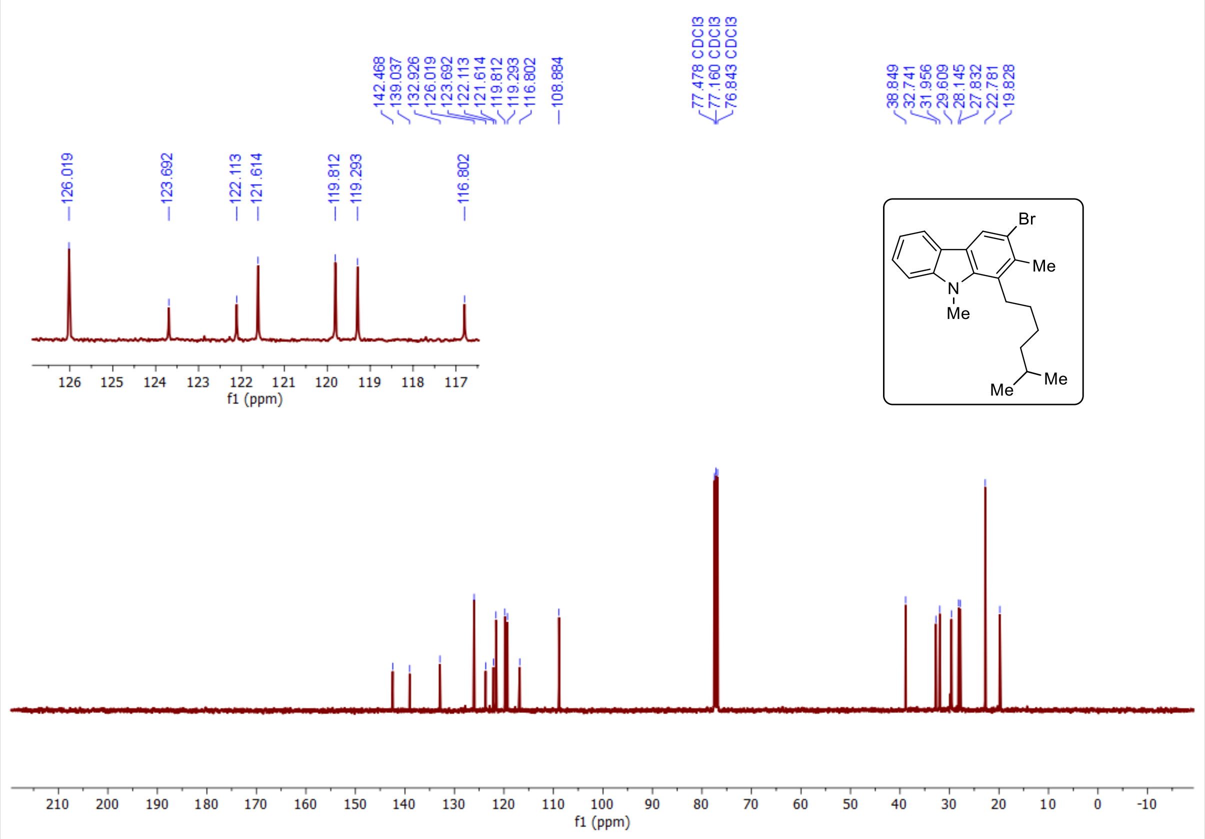

^{1}H NMR (500 MHz, $CDCl_3$) and **^{13}C[^{1}H] NMR** (125 MHz, $CDCl_3$) of **(14a)**

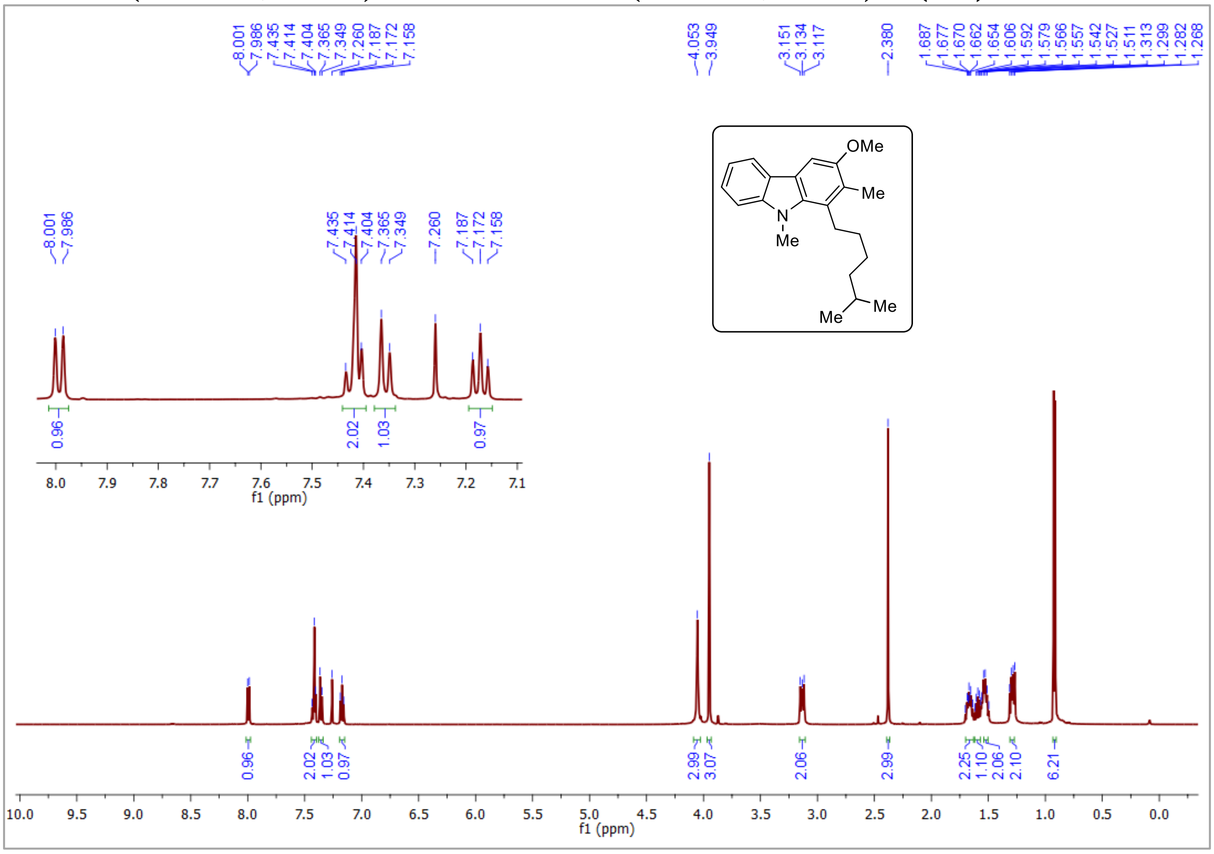

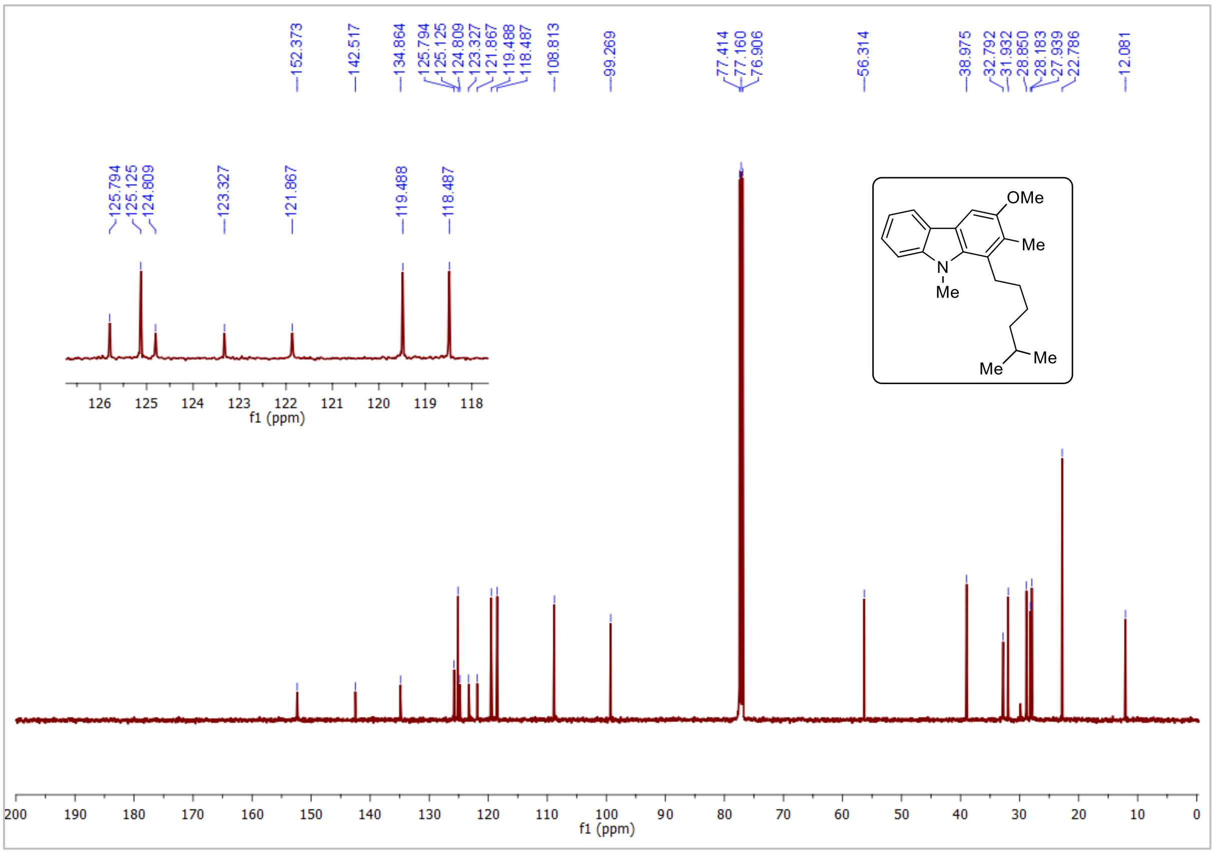

^{1}H NMR (400 MHz, $CDCl_3$) and **^{13}C[^{1}H] NMR** (100 MHz, $CDCl_3$) of **(15a)**

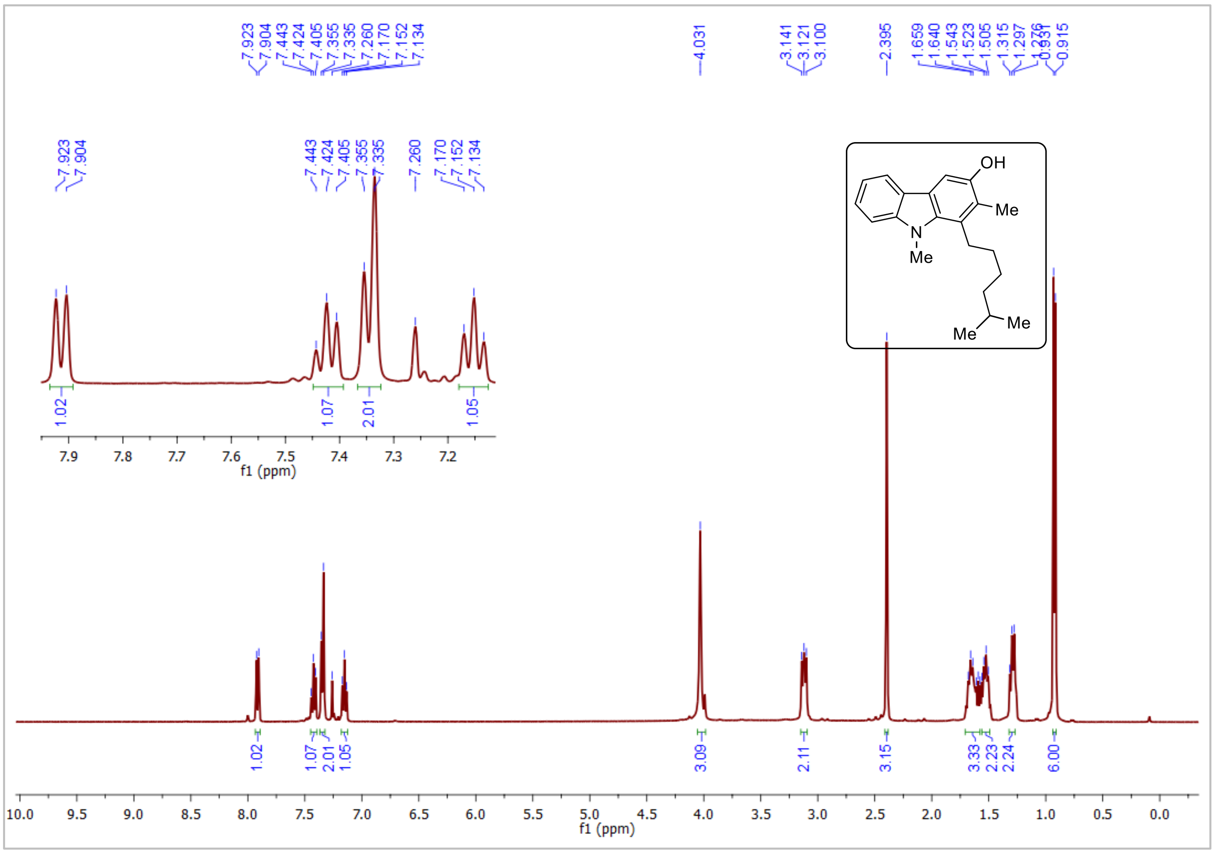

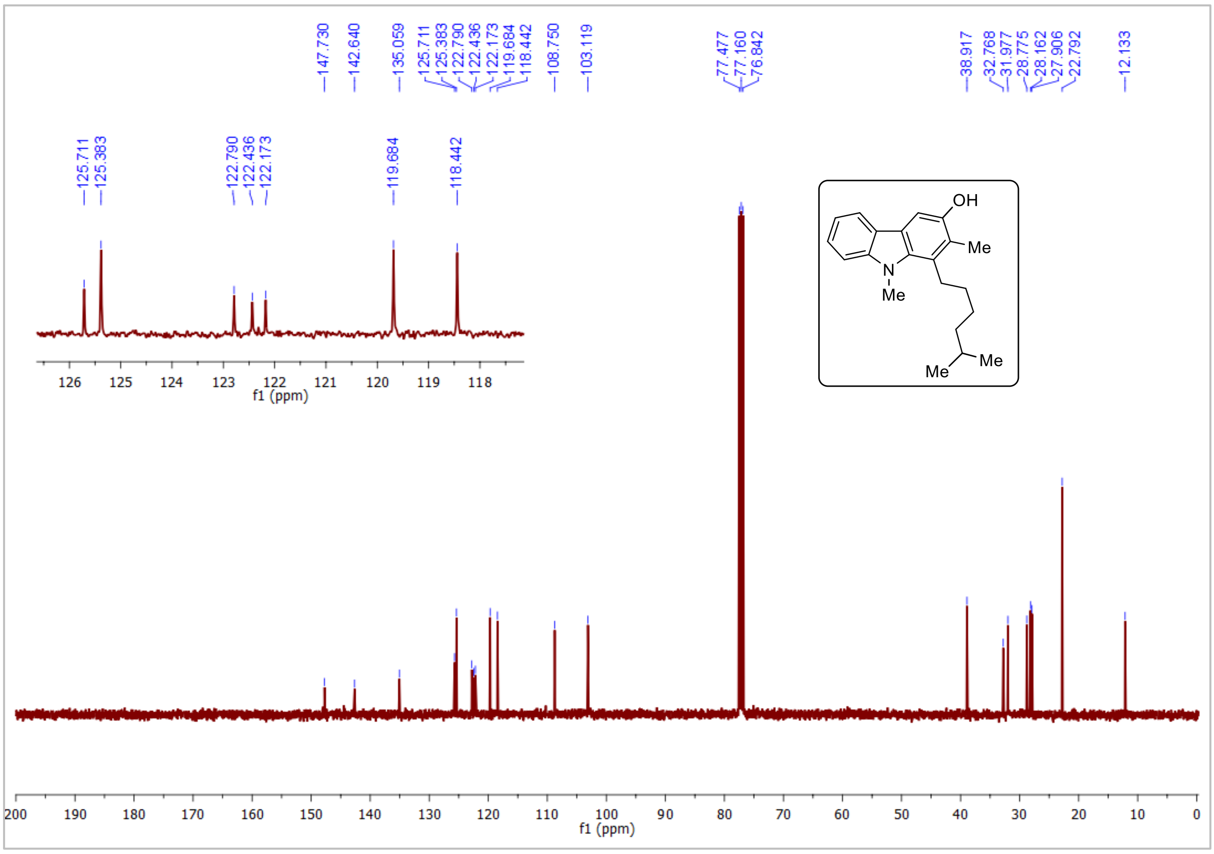

^{1}H NMR (400 MHz, $CDCl_3$) and ^{13}C[^{1}H] NMR (100 MHz, $CDCl_3$) of **(3)**

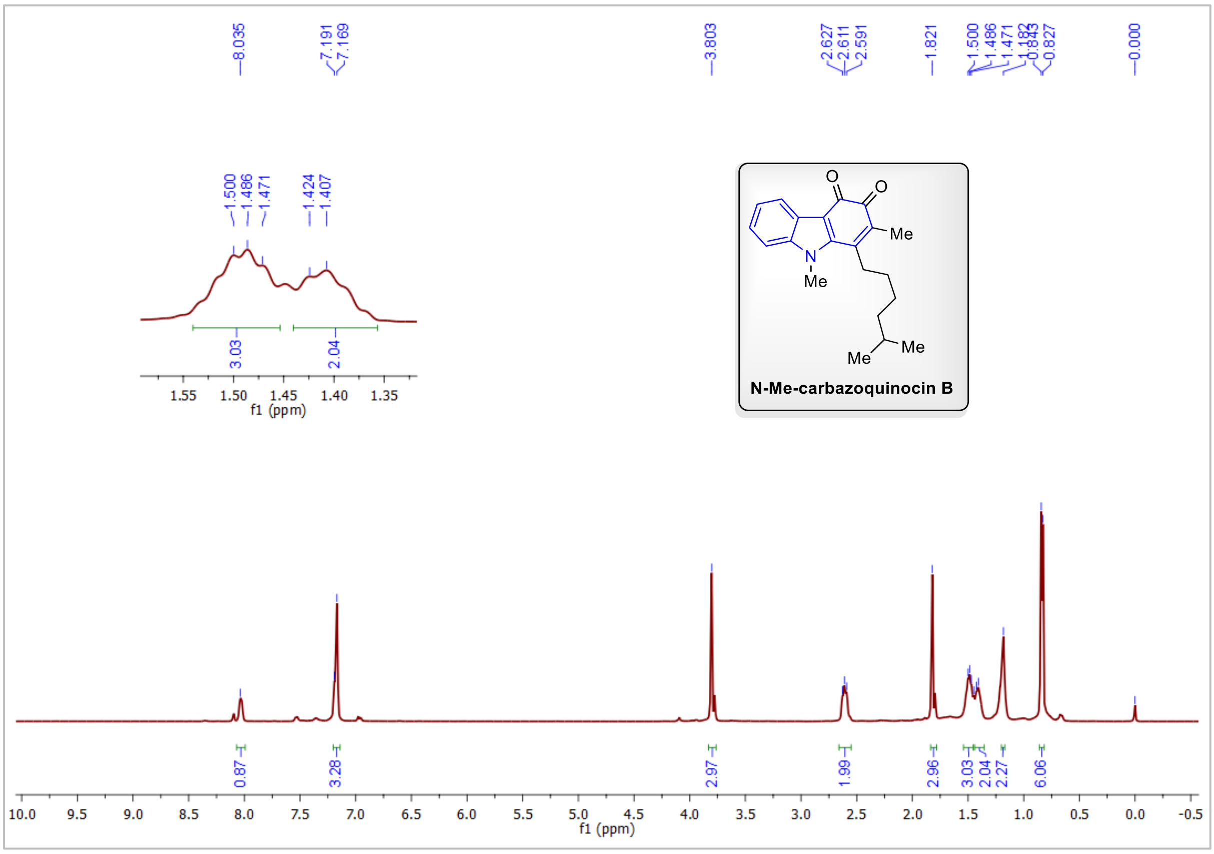

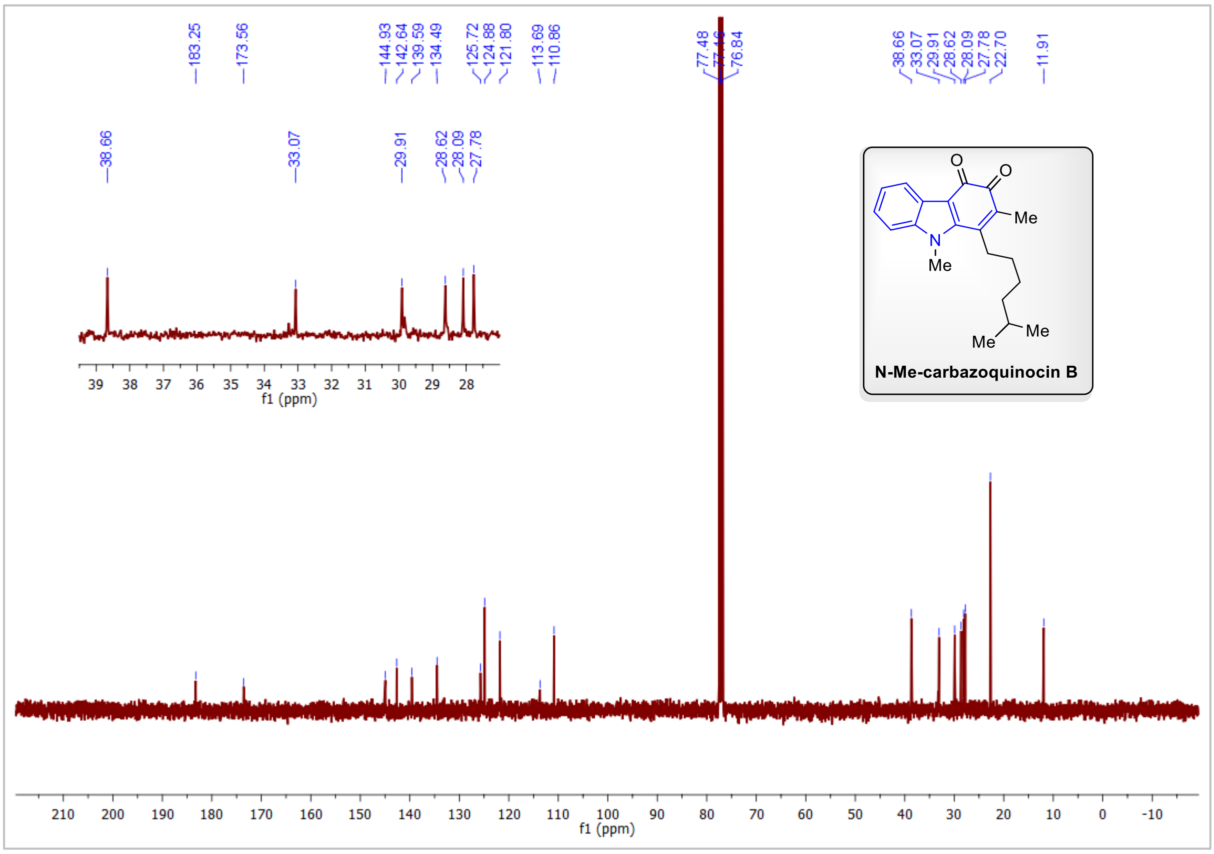

^{1}H NMR (400 MHz, $CDCl_3$) and **$^{13}C[^1H]$ NMR** (100 MHz, $CDCl_3$) of **(11b)**

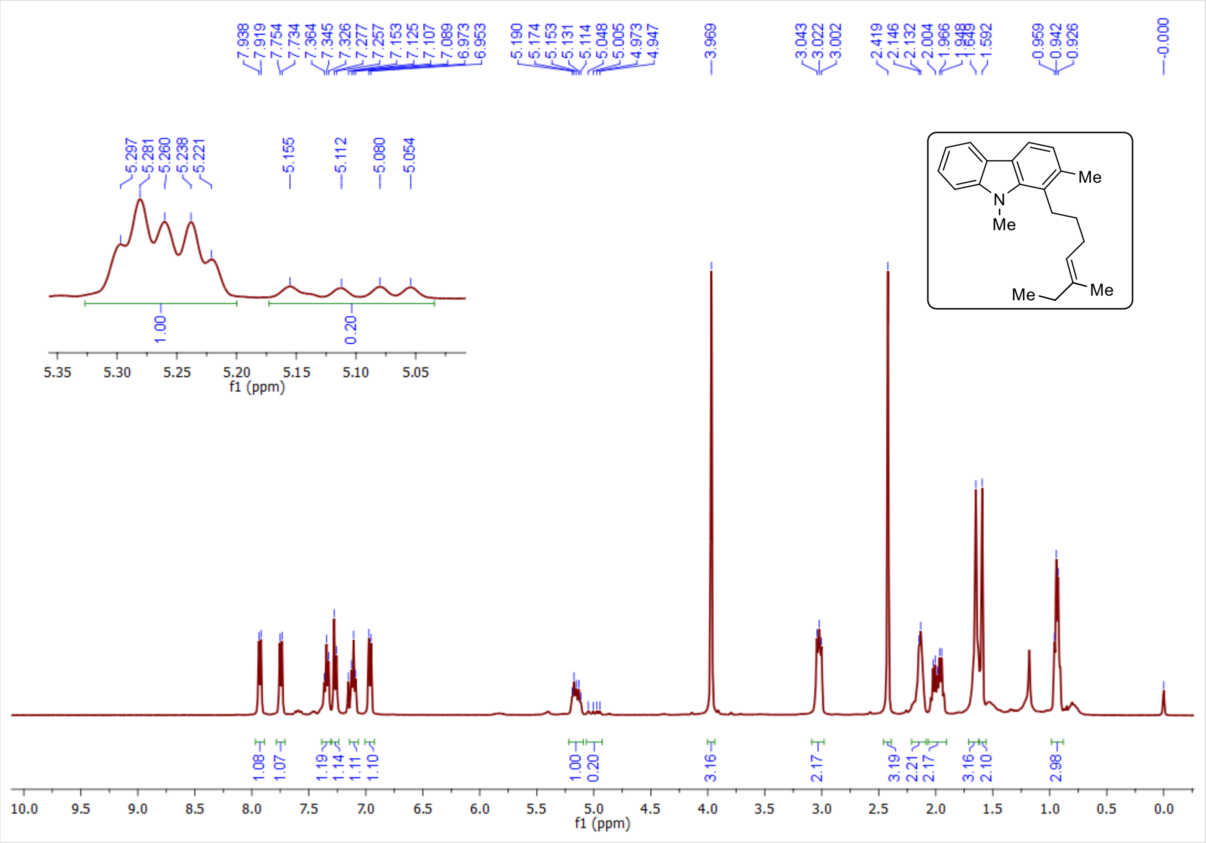

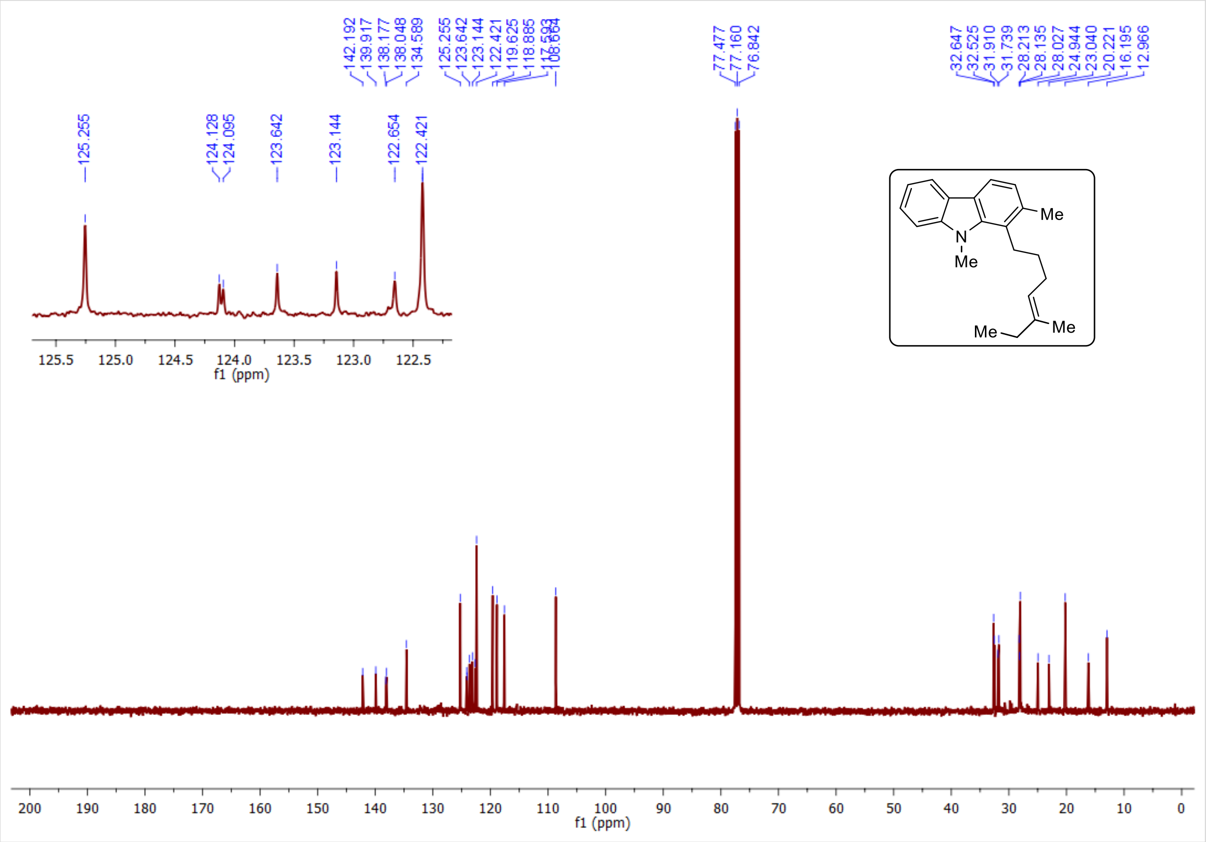

^{1}H **NMR** (400 MHz, $CDCl_3$) and $^{13}C[^{1}H]$ **NMR** (100 MHz, $CDCl_3$) of **(12b)**

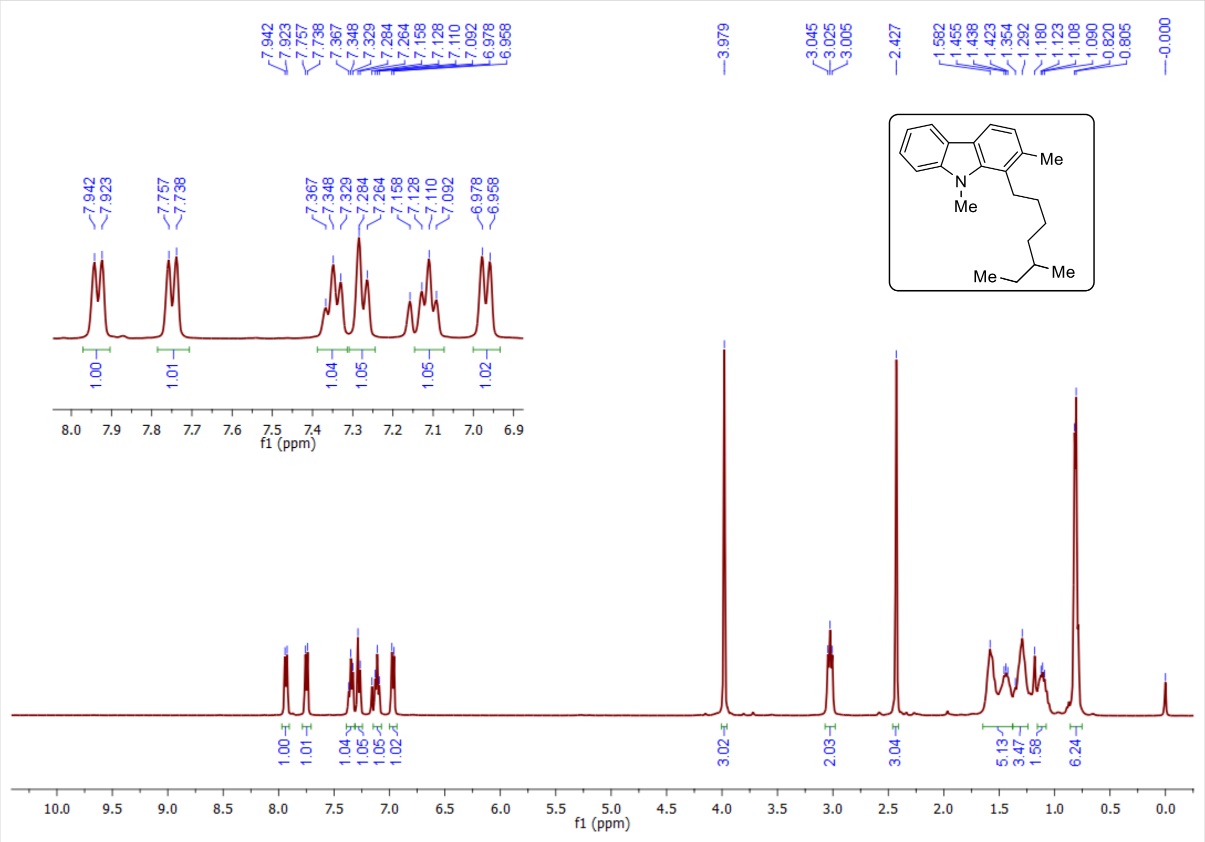

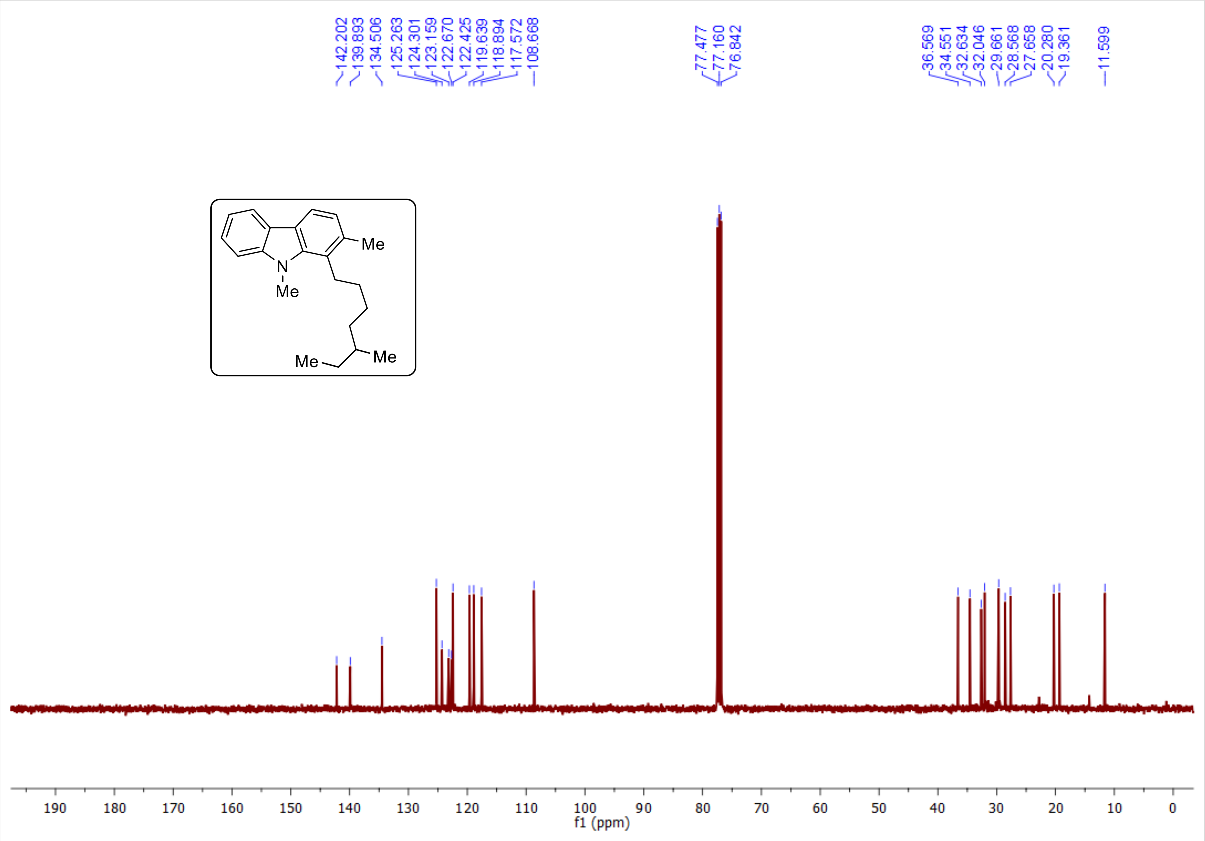

^{1}H NMR (400 MHz, $CDCl_3$) and **^{13}C[^{1}H] NMR** (100 MHz, $CDCl_3$) of **(13b)**

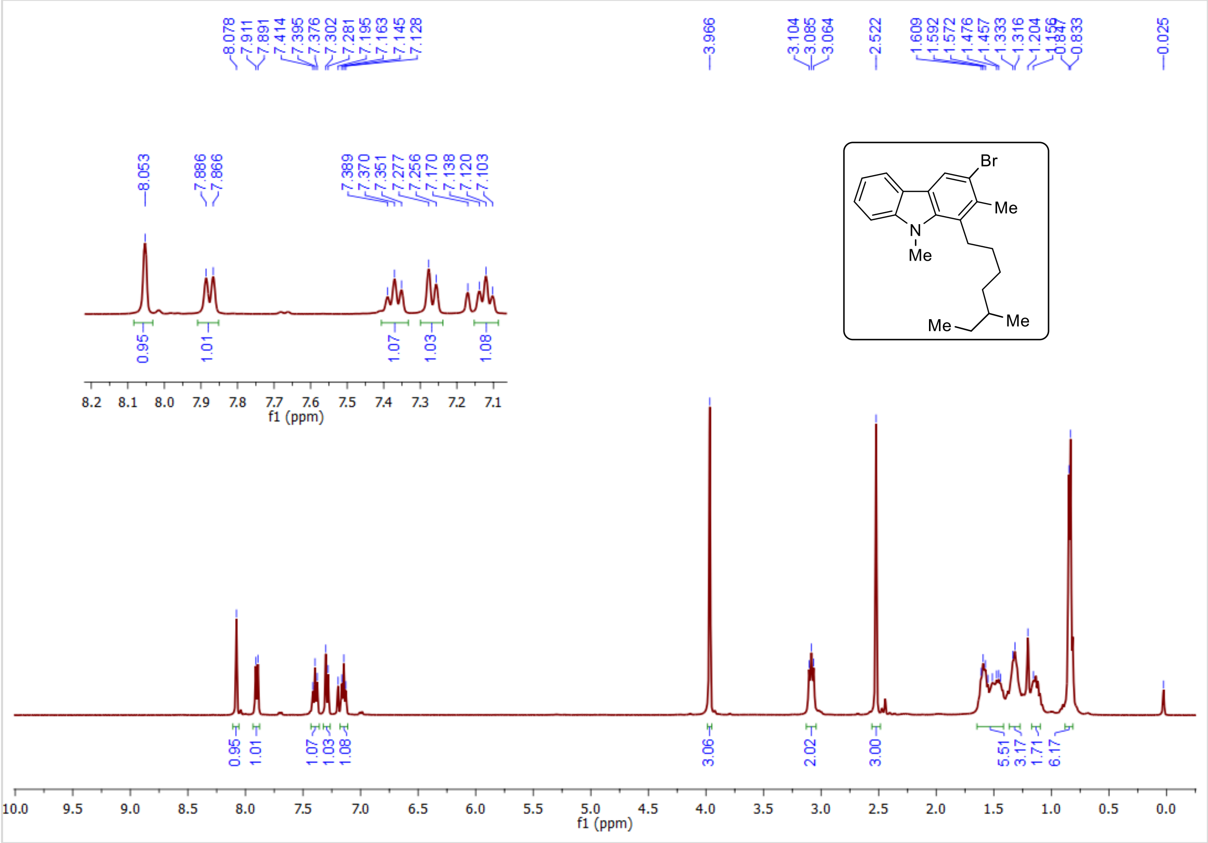

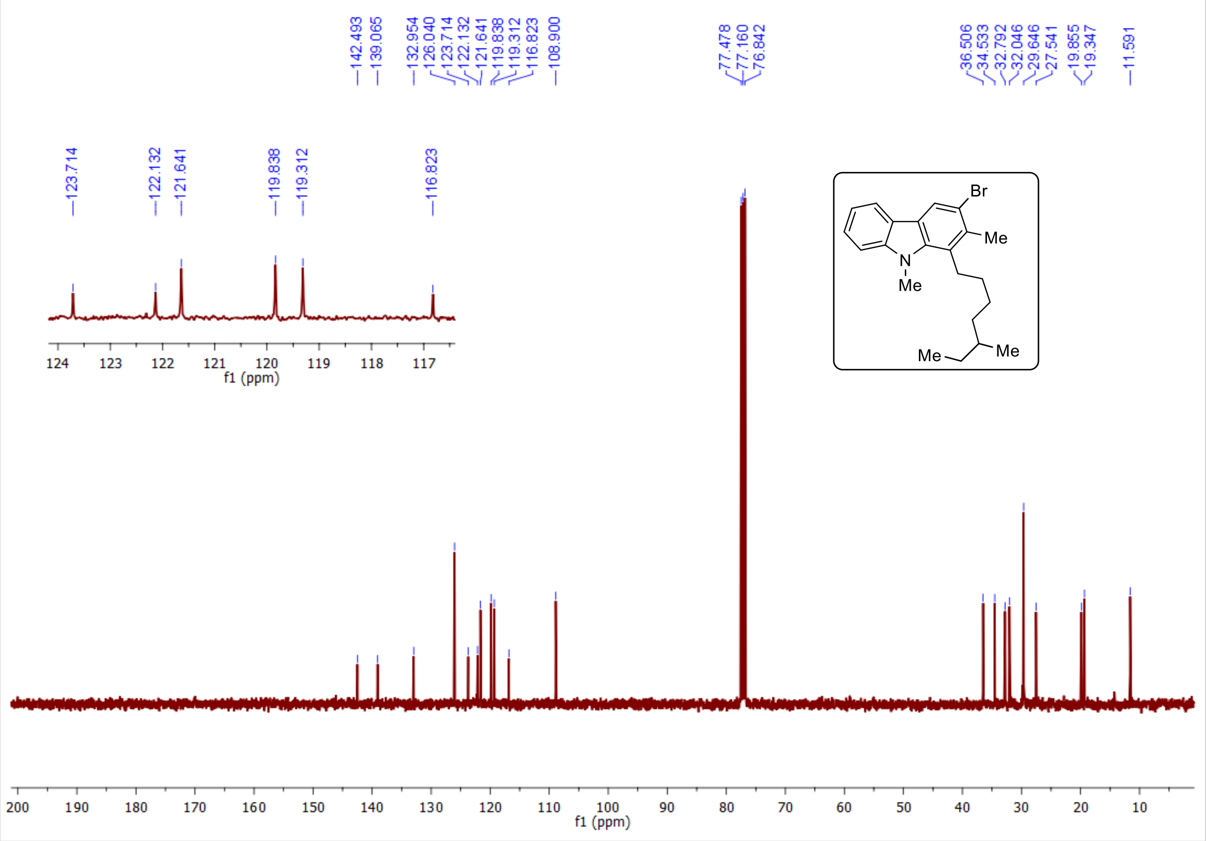

^{1}H NMR (500 MHz, $CDCl_3$) and ^{13}C[^{1}H] NMR (125 MHz, $CDCl_3$) of **(14b)**

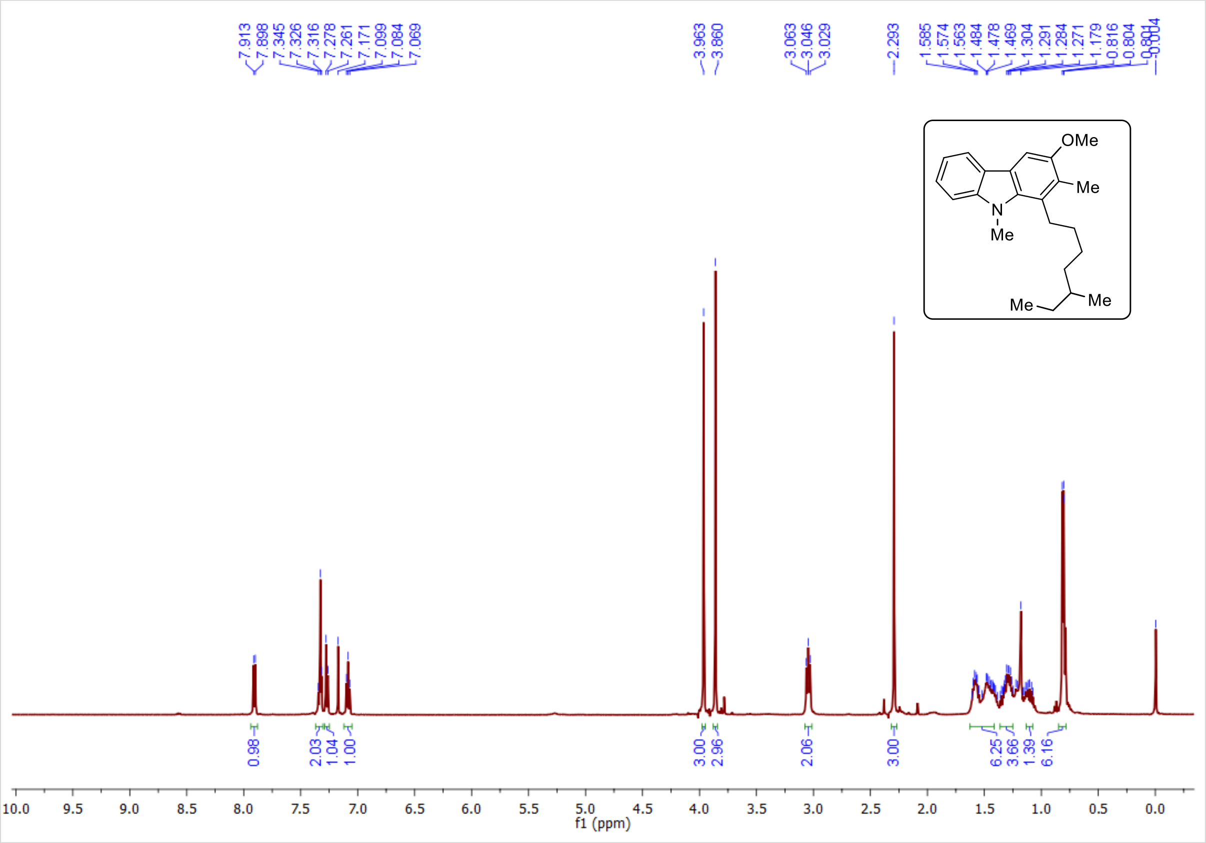

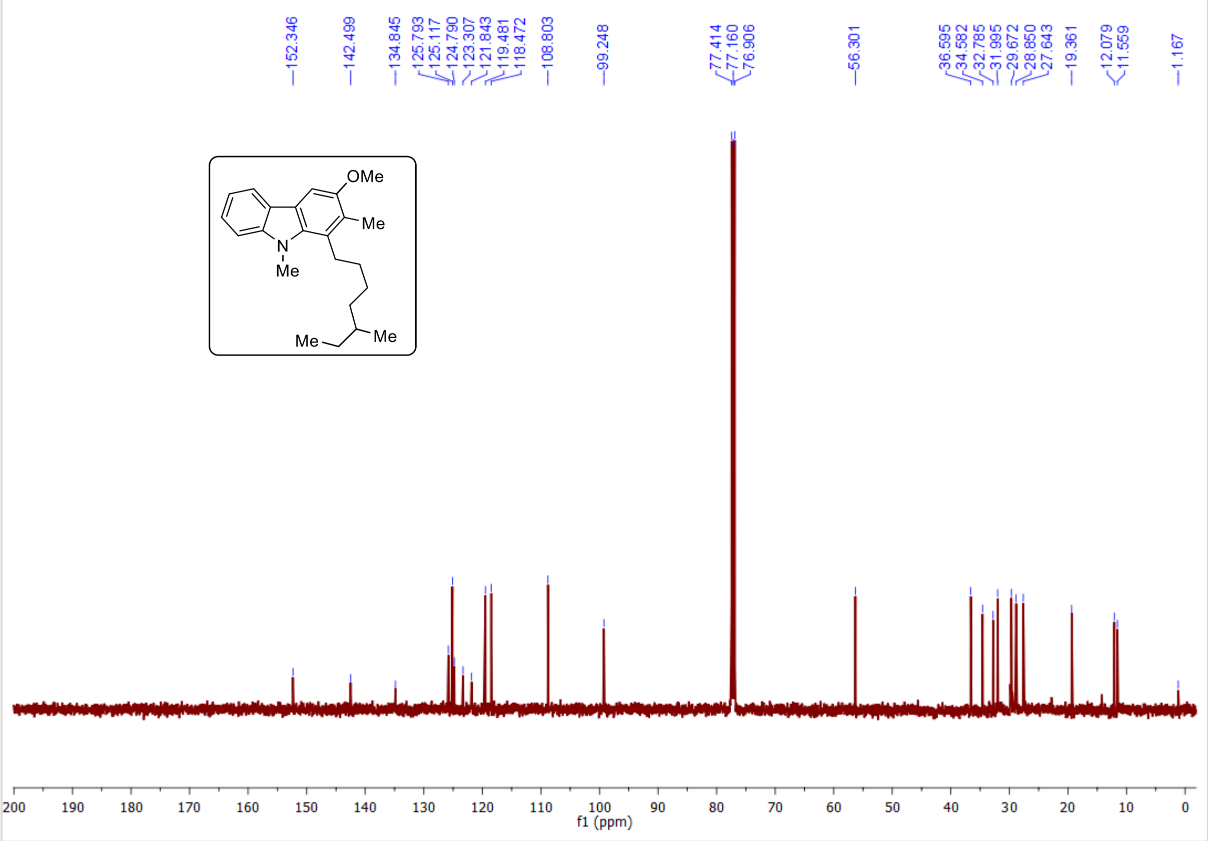

^{1}H NMR (400 MHz, $CDCl_3$) and **^{13}C[^{1}H] NMR** (100 MHz, $CDCl_3$) of **(15b)**

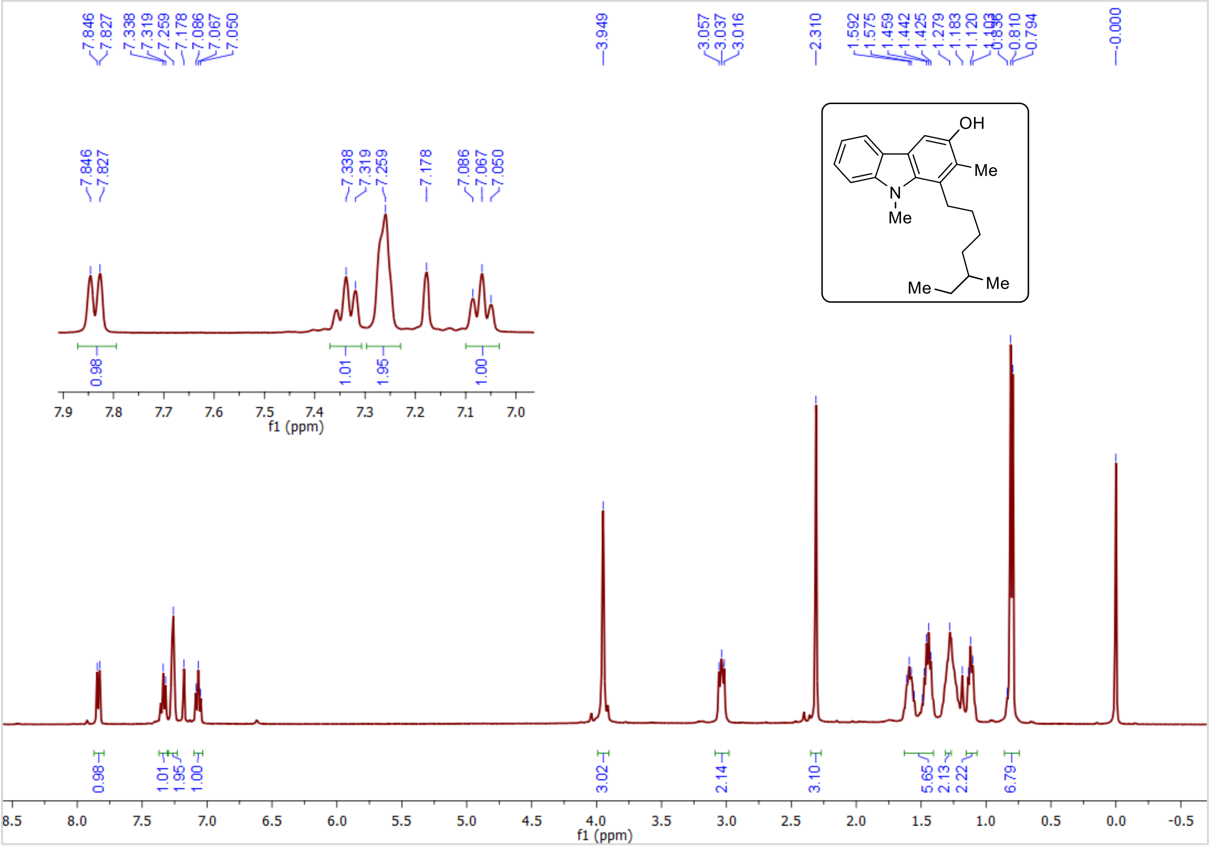

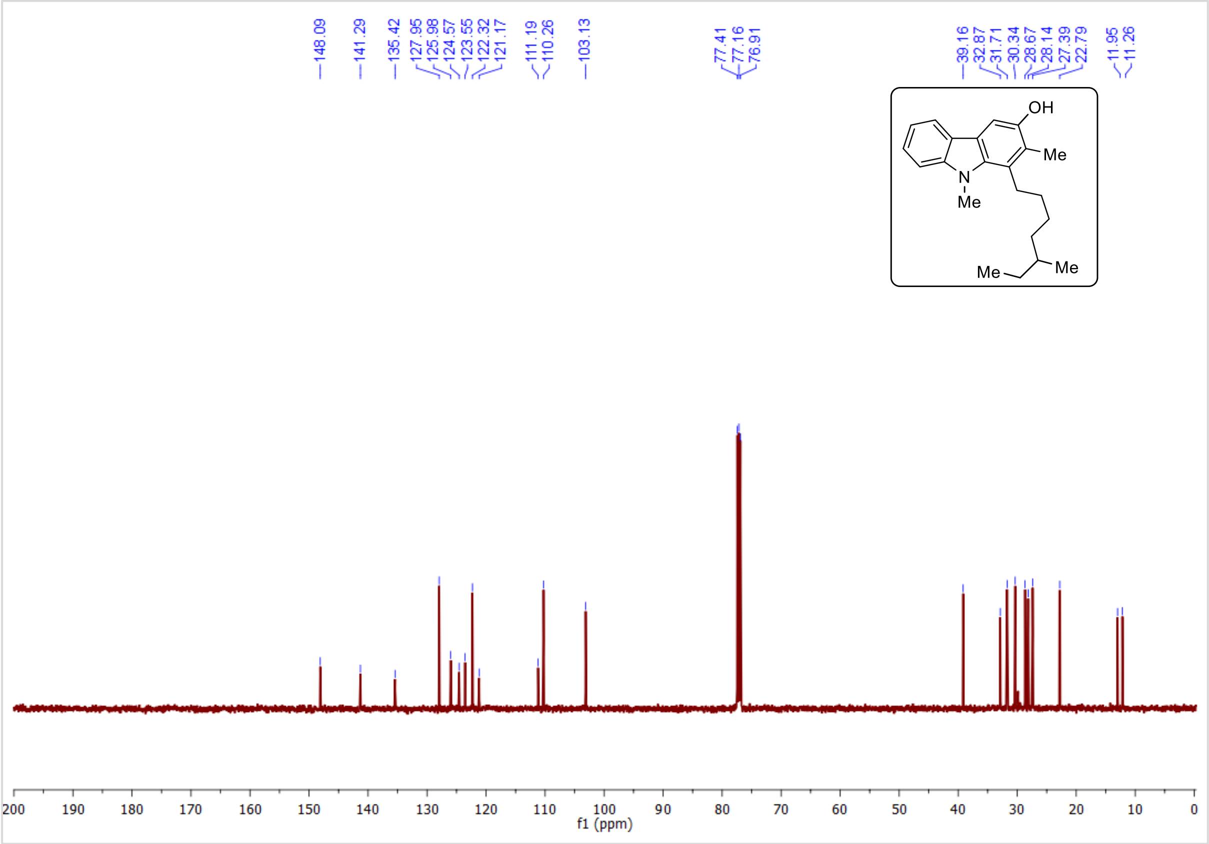

^{1}H **NMR** (500 MHz, $CDCl_3$) and $^{13}C[^{1}H]$ **NMR** (125 MHz, $CDCl_3$) of **(4)**

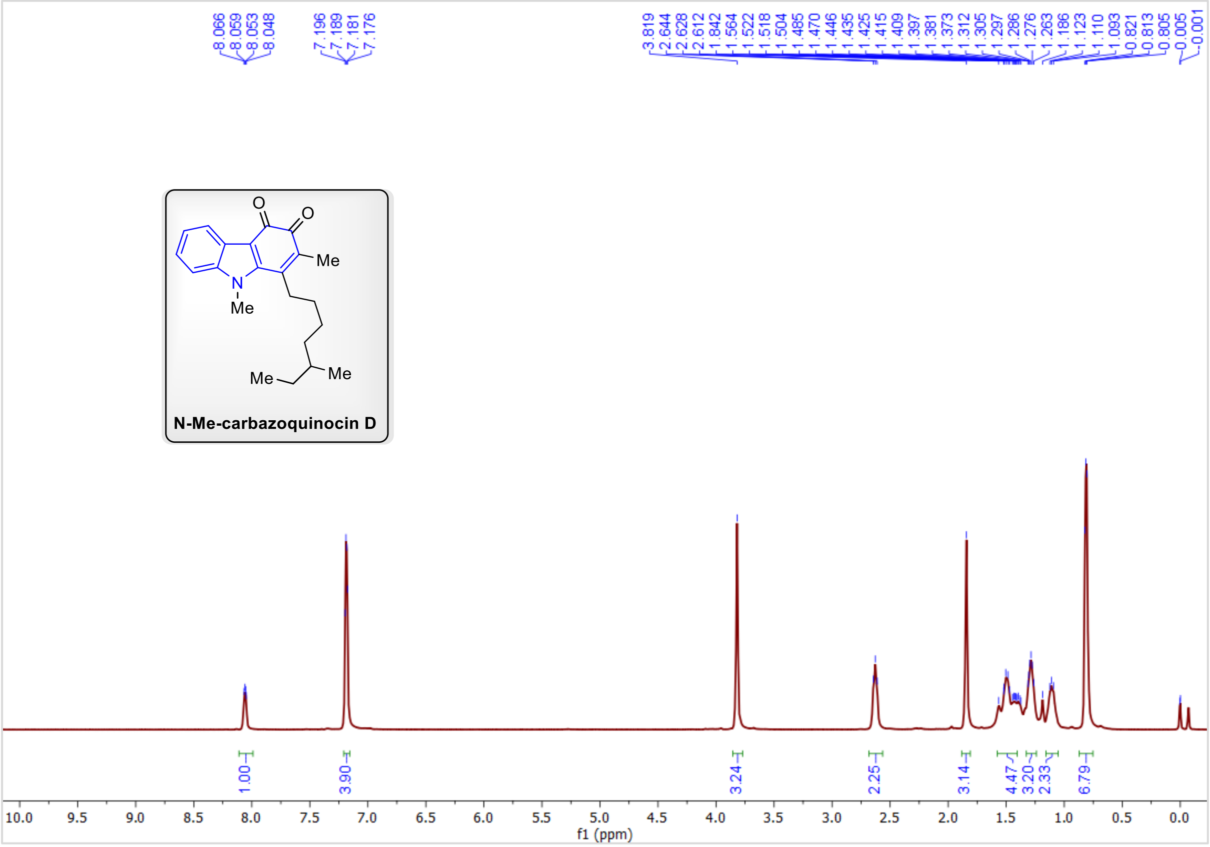

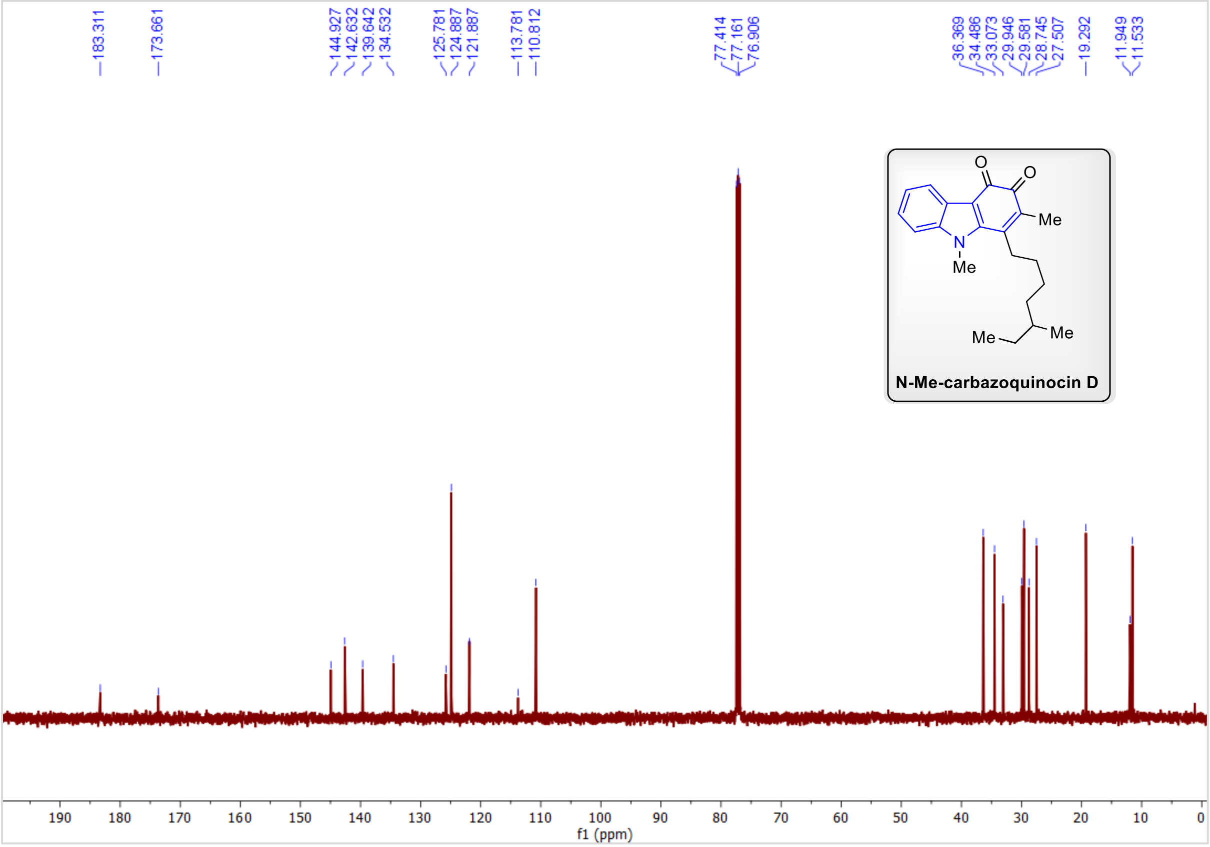

^{1}H NMR (500 MHz, $CDCl_3$) and ^{13}C[^{1}H] NMR (125 MHz, $CDCl_3$) of (**11c**)

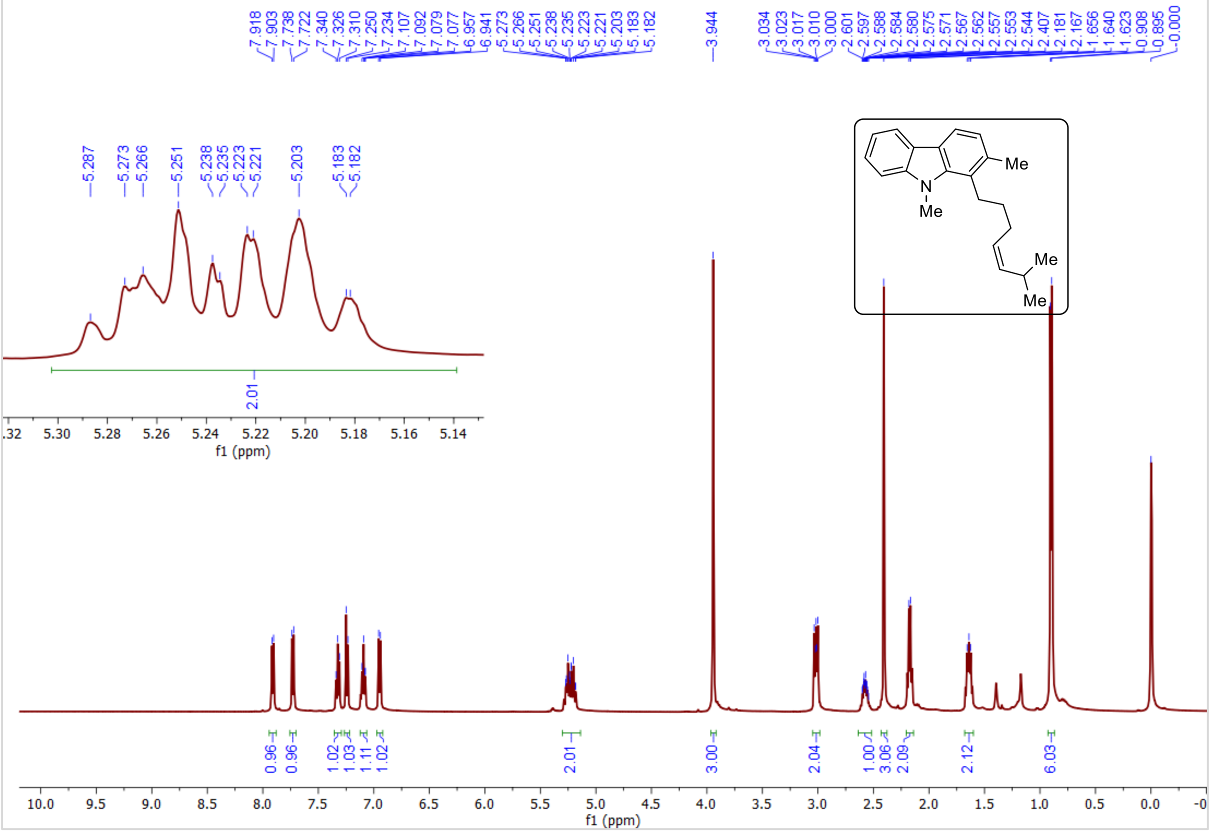

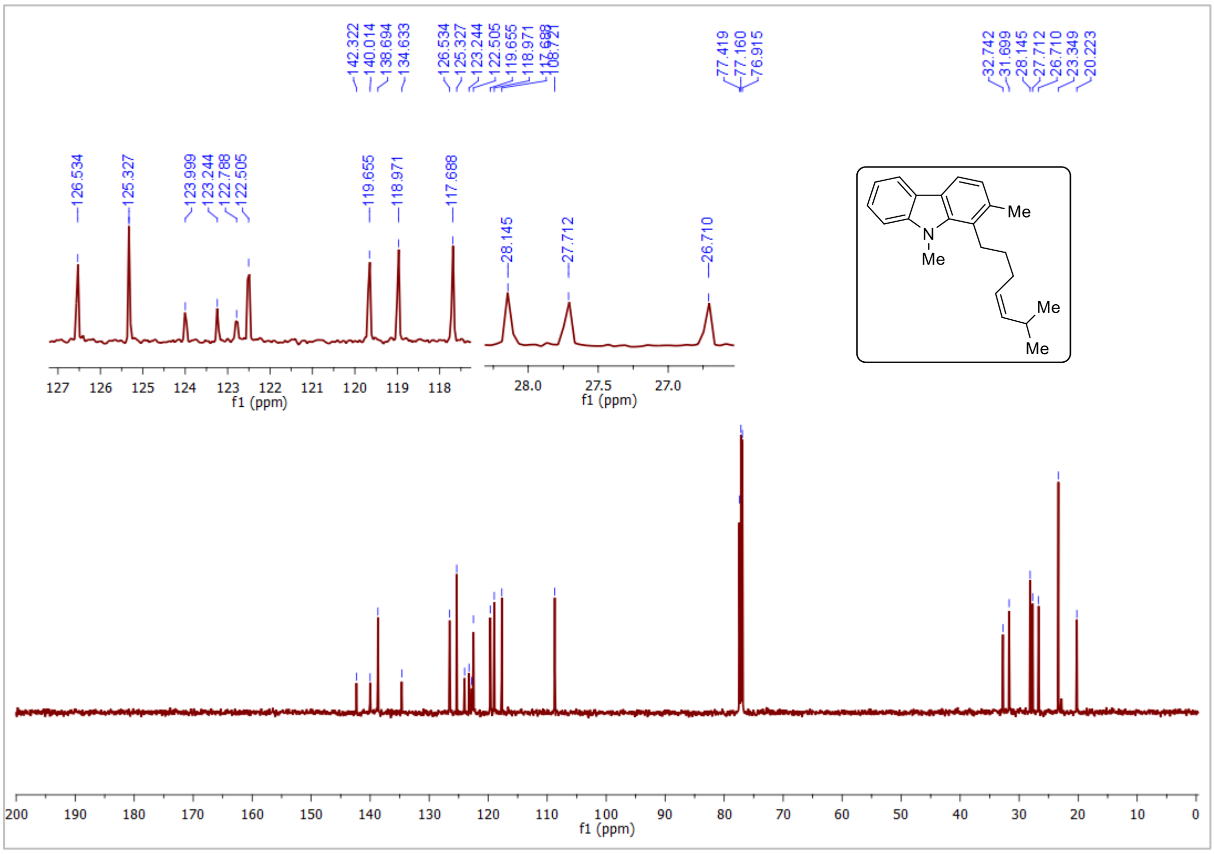

^{1}H NMR (500 MHz, $CDCl_3$) and **^{13}C[^{1}H] NMR** (125 MHz, $CDCl_3$) of **(12c)**

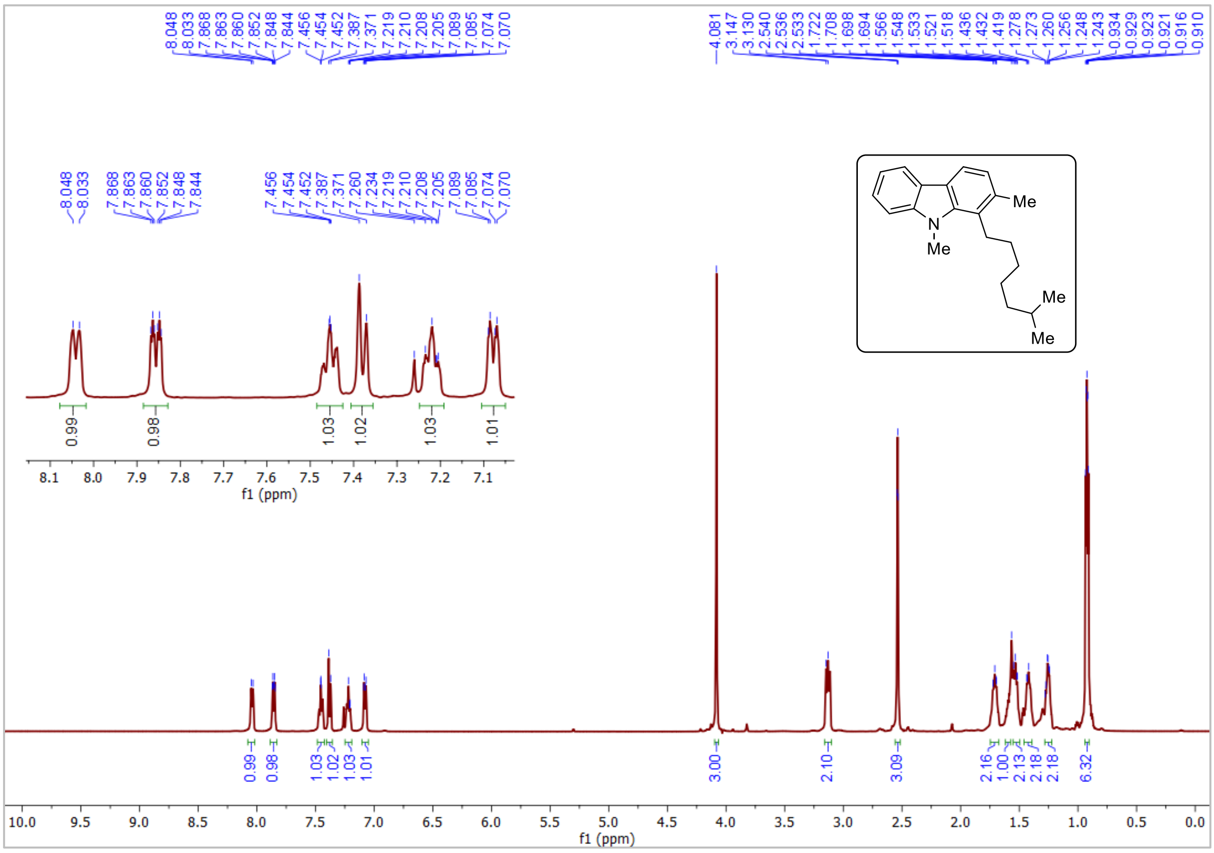

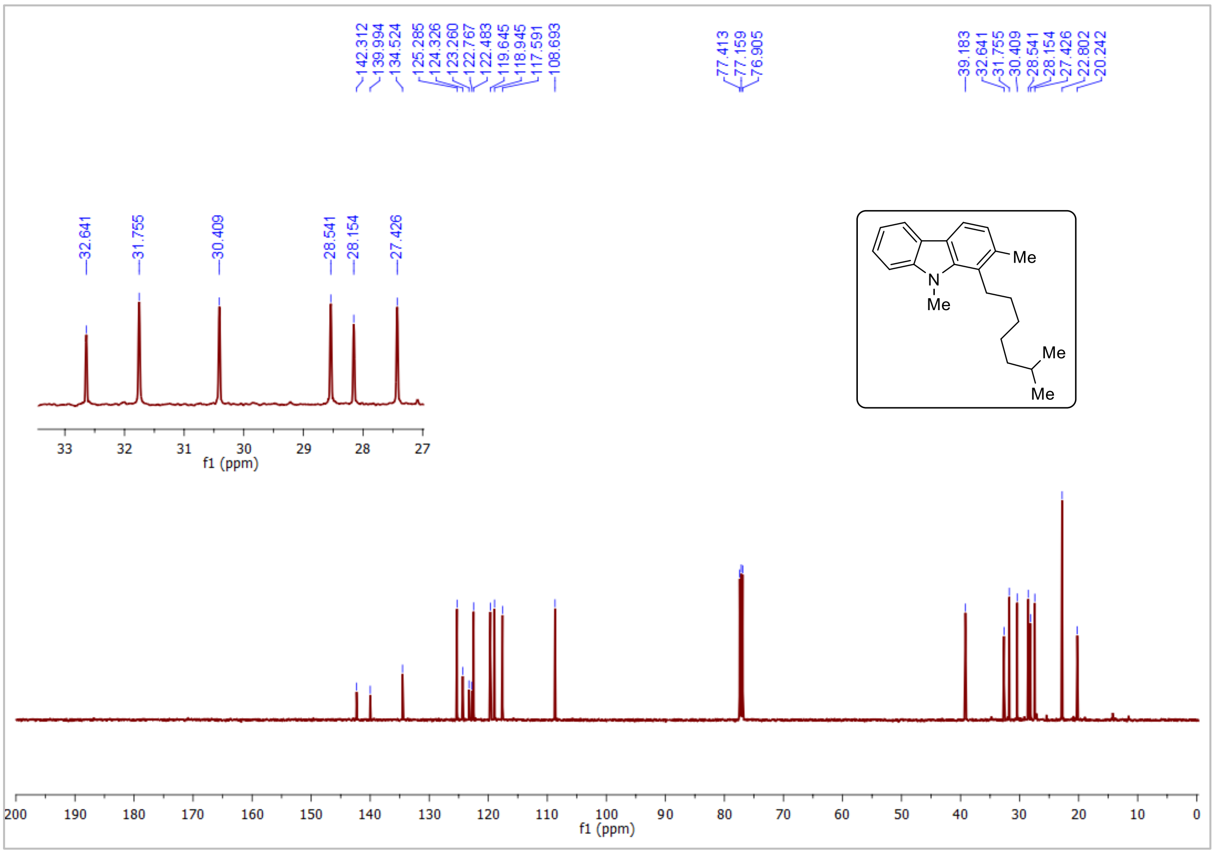

^{1}**H NMR** (500 MHz, $CDCl_3$) and ^{13}**C[^{1}H] NMR** (125 MHz, $CDCl_3$) of **(13c)**

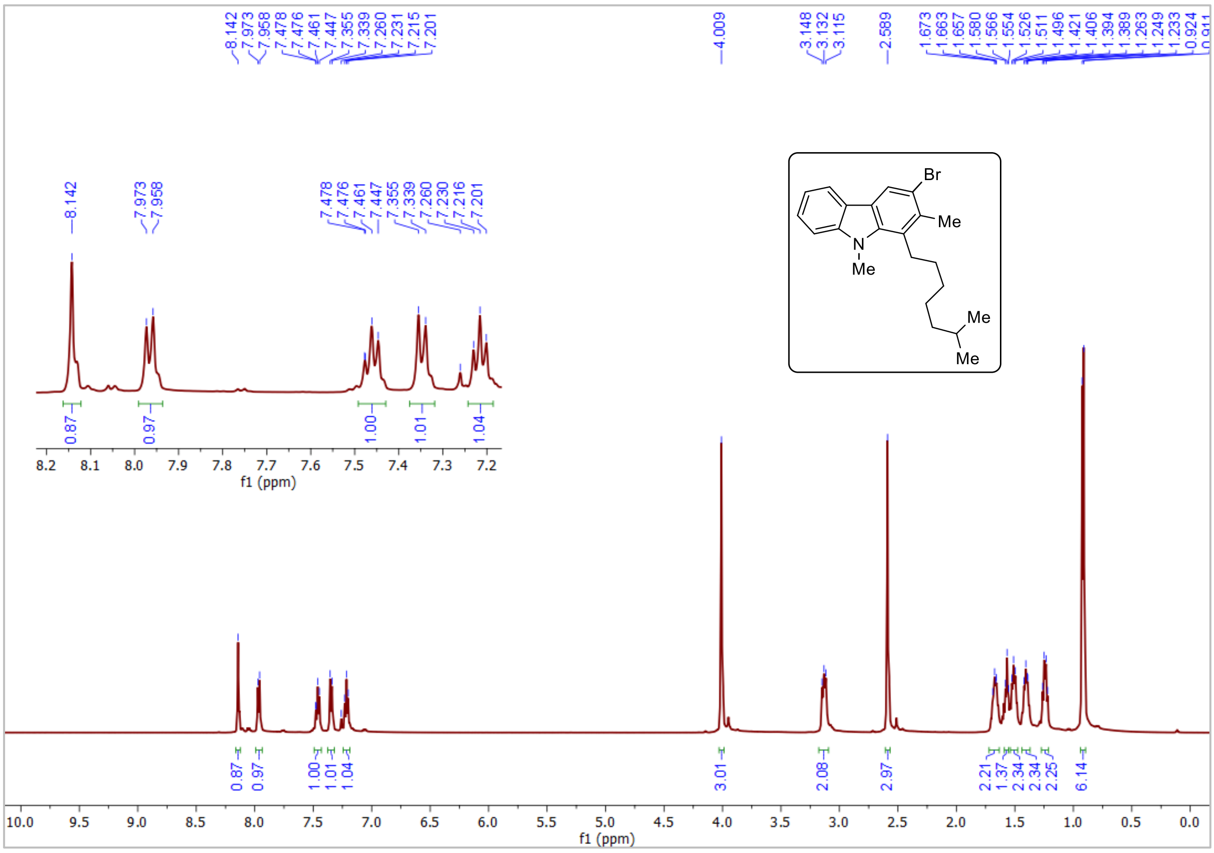

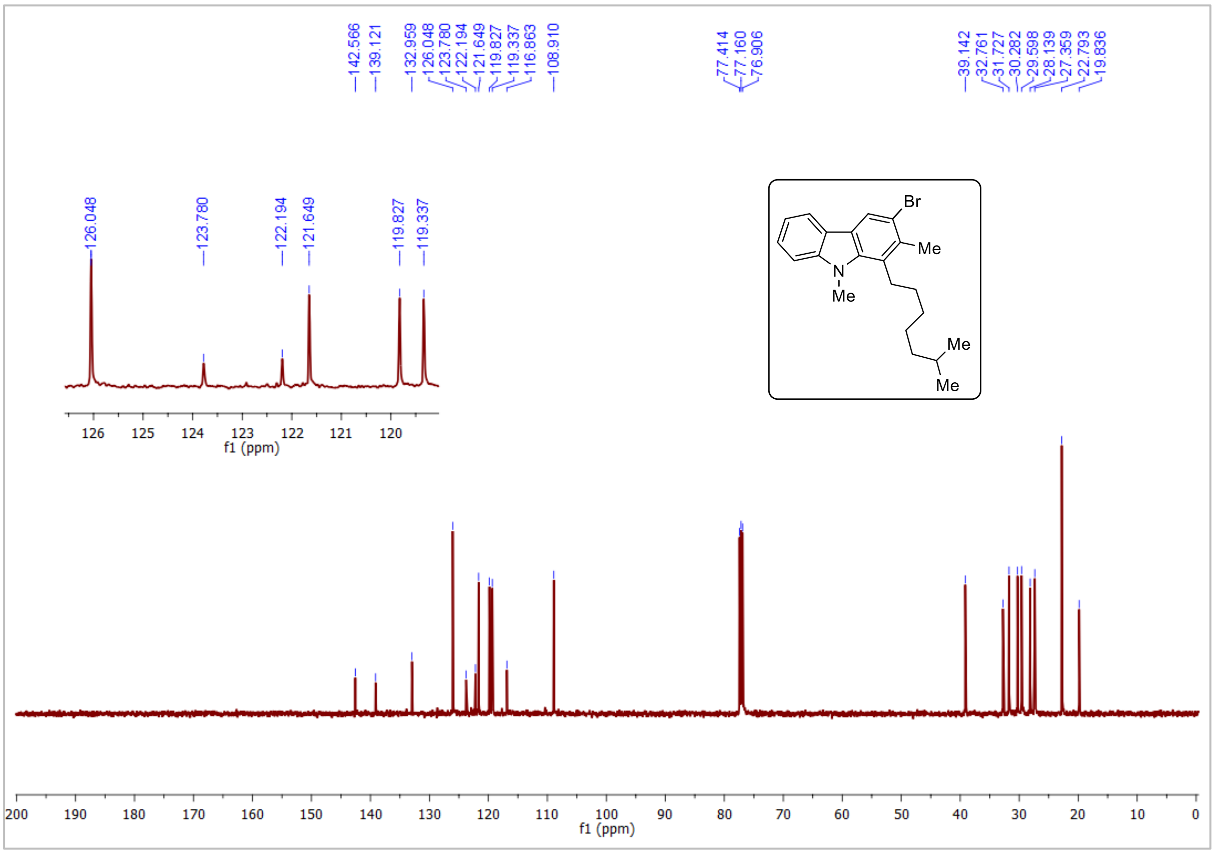

^{1}H NMR (500 MHz, $CDCl_3$) and **^{13}C[^{1}H] NMR** (125 MHz, $CDCl_3$) of **(14c)**

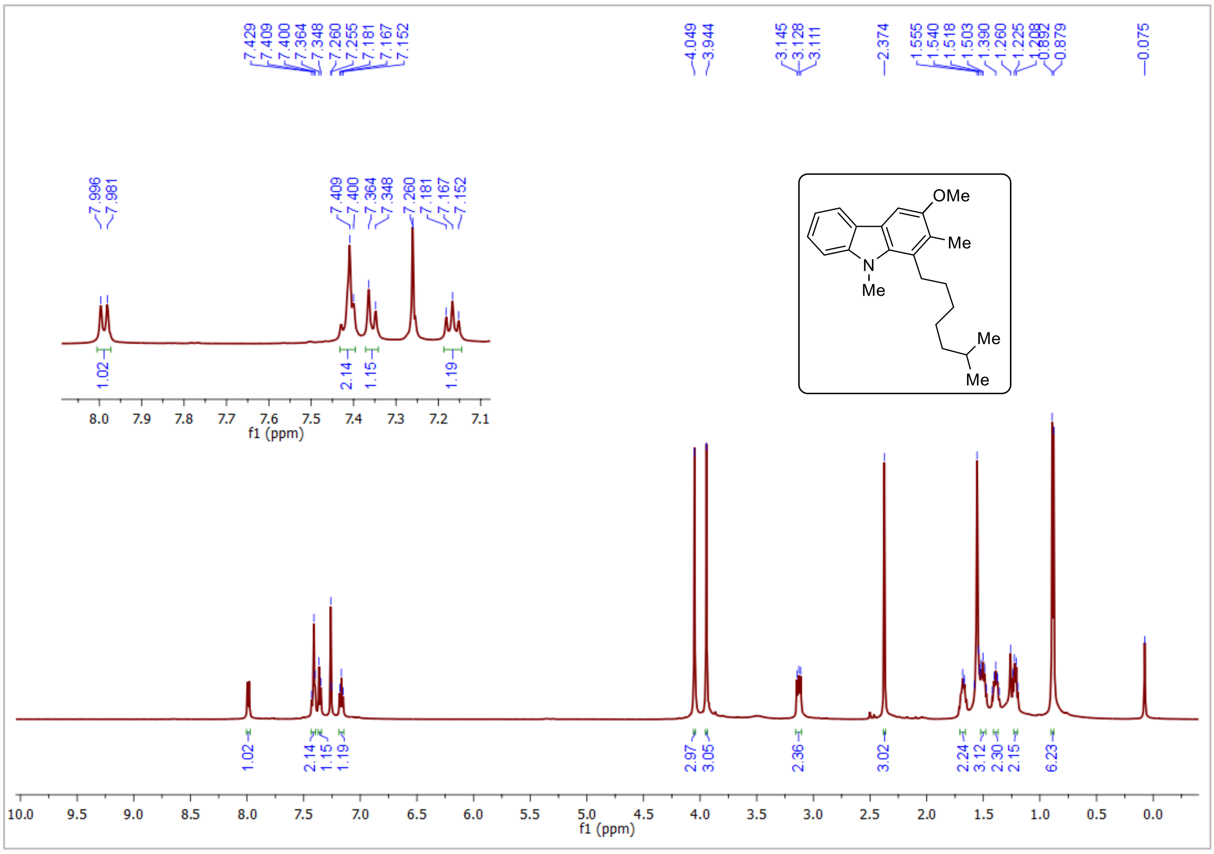

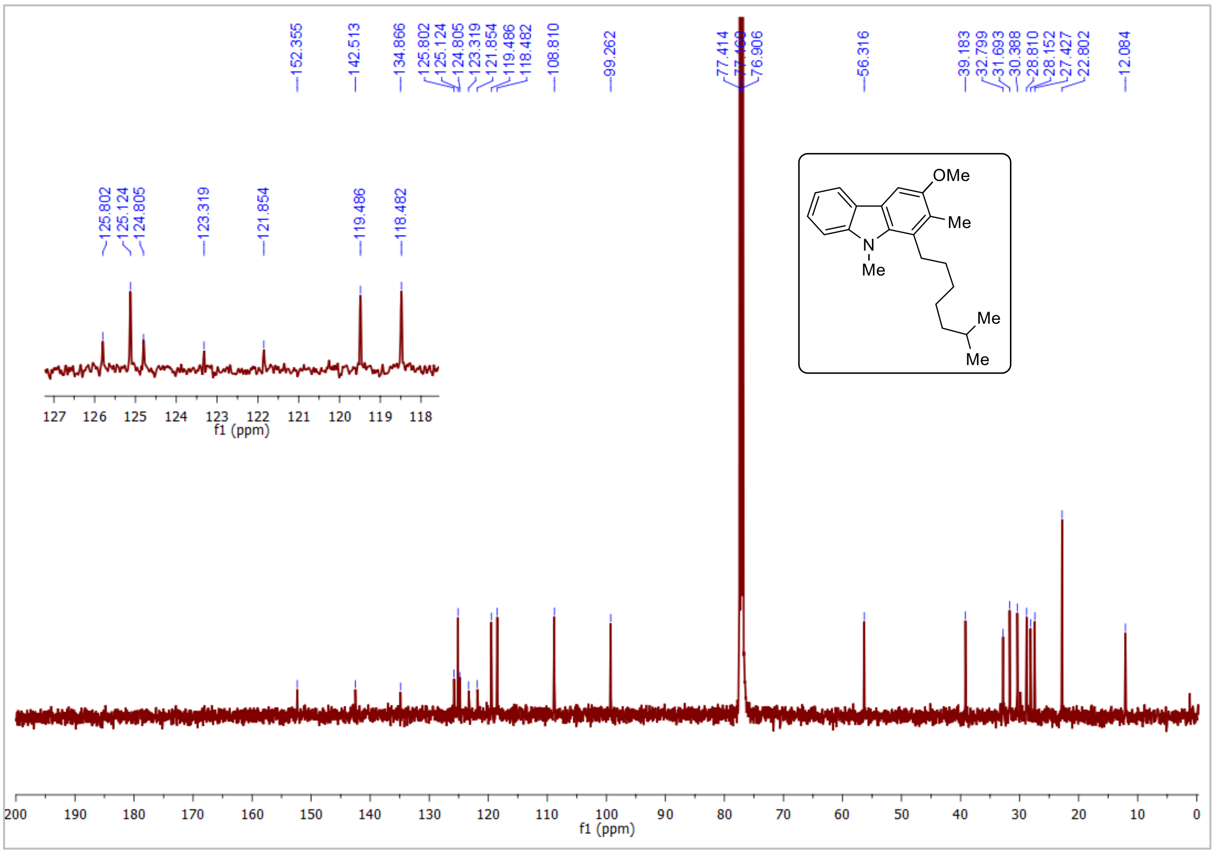

^{1}H NMR (500 MHz, $CDCl_3$) and **^{13}C[^{1}H] NMR** (125 MHz, $CDCl_3$) of **(15c)**

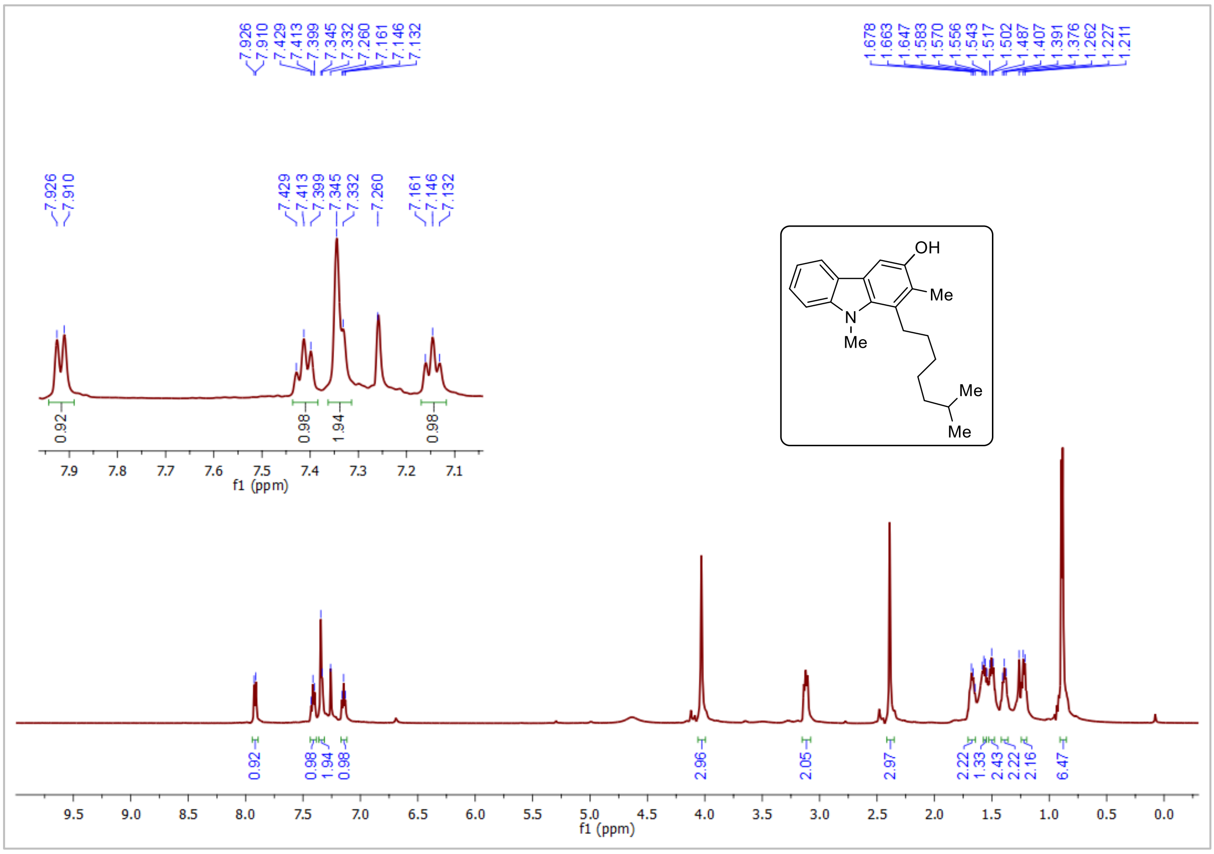

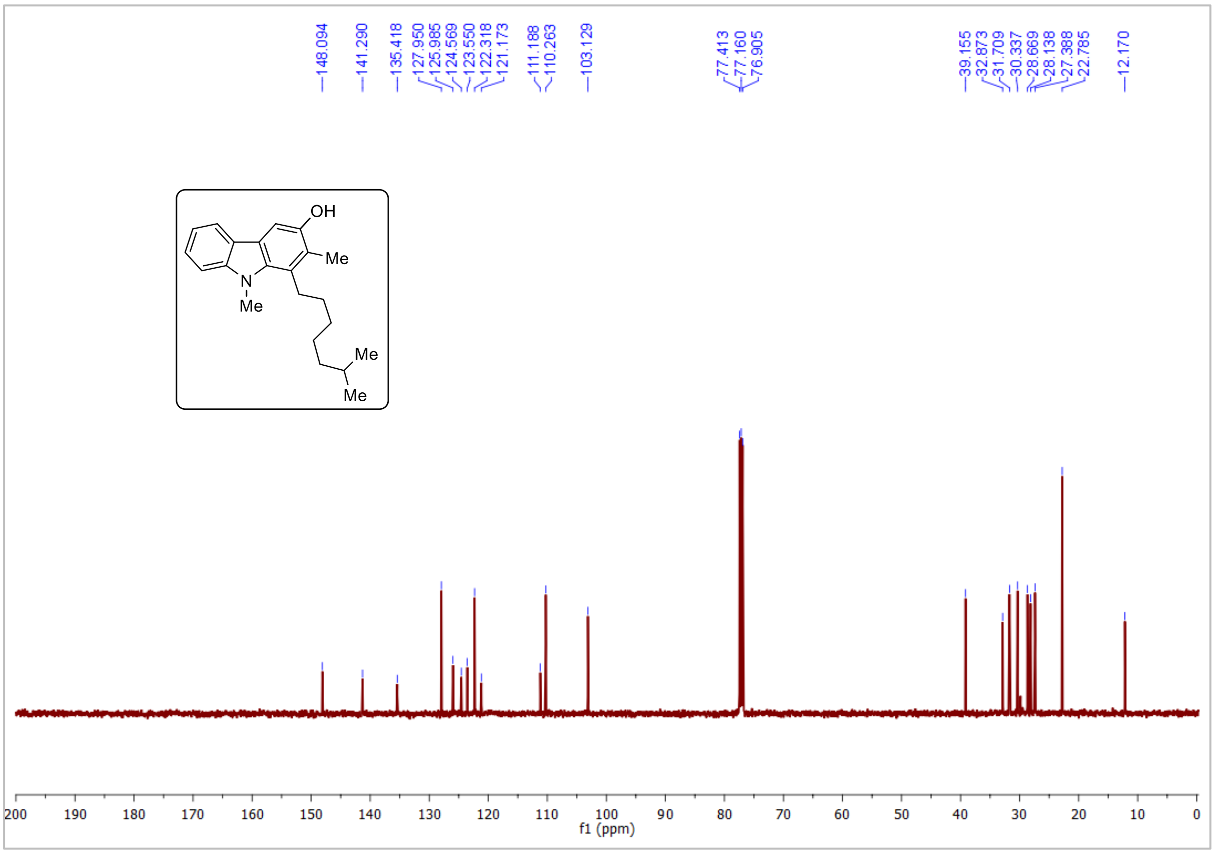

^{1}H NMR (500 MHz, $CDCl_3$) and **^{13}C[^{1}H] NMR** (125 MHz, $CDCl_3$) of **(5)**

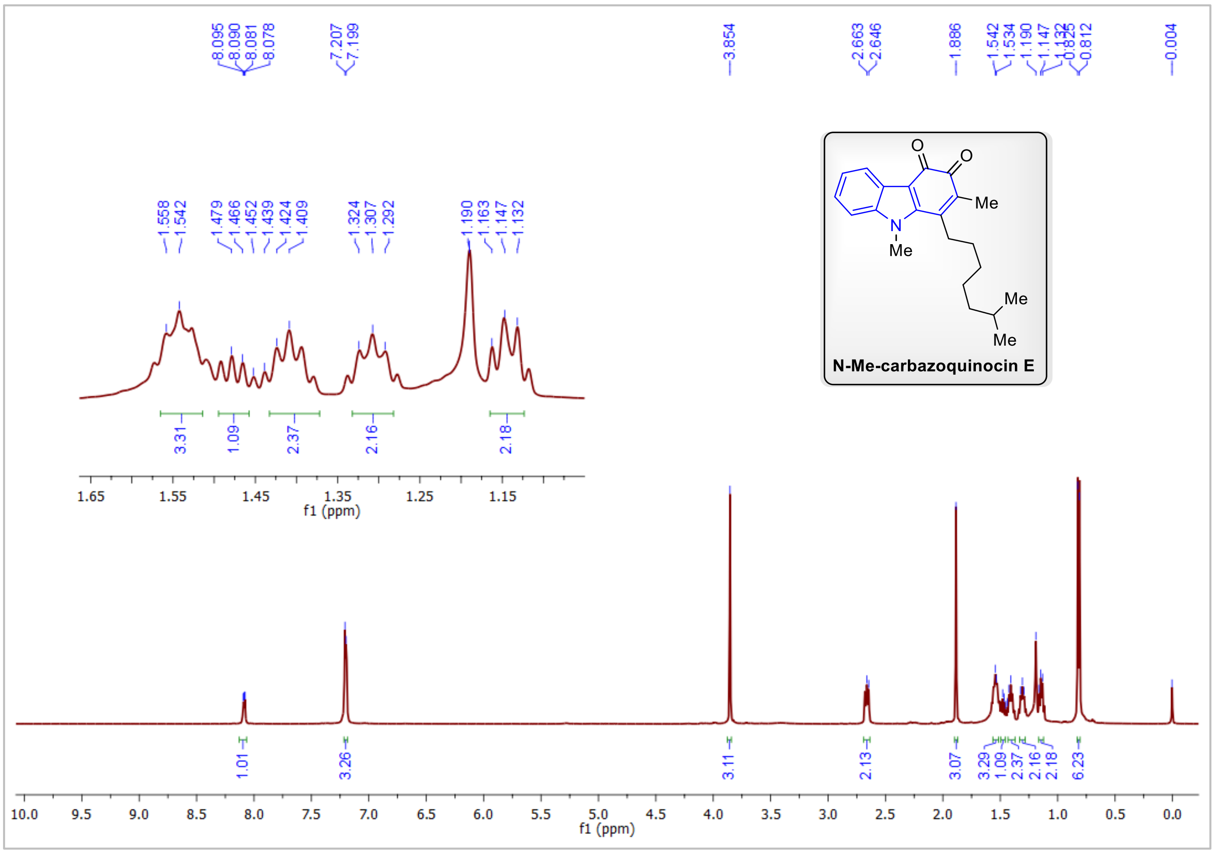

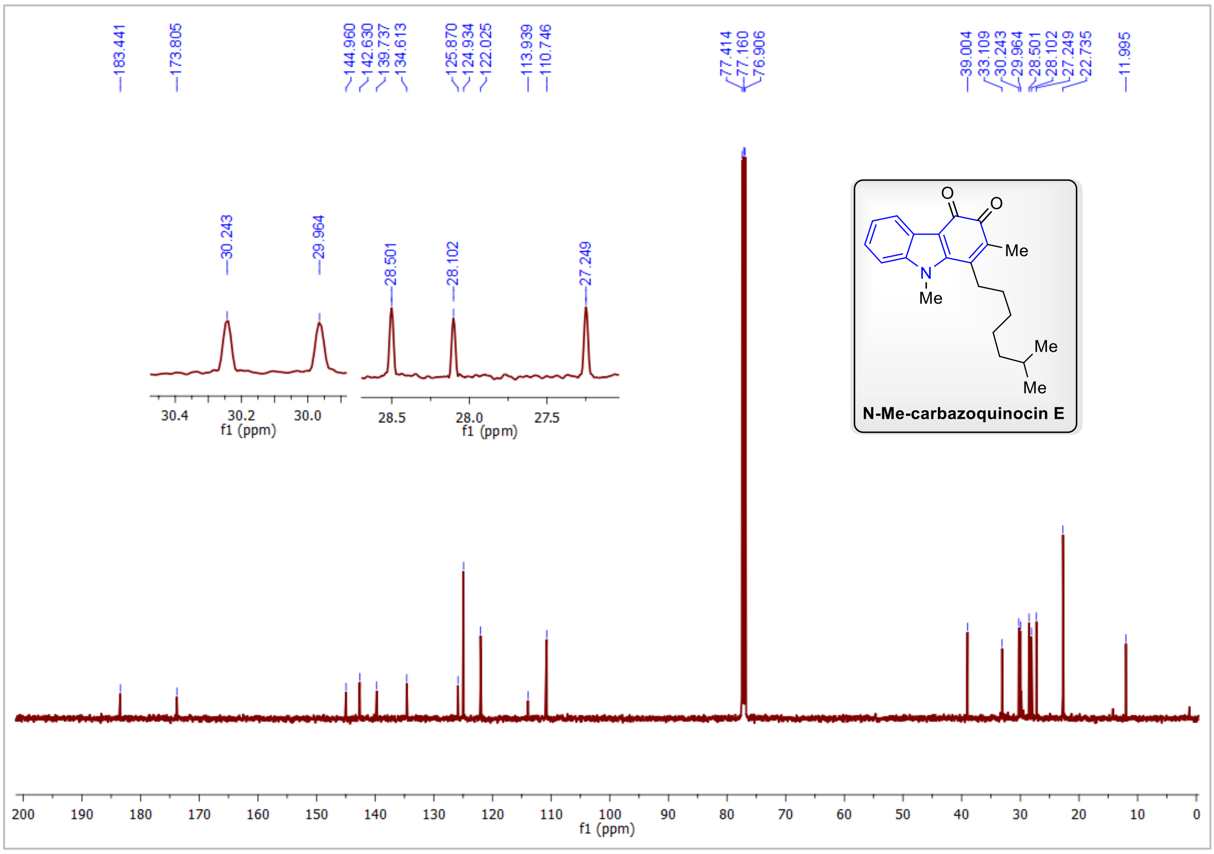

^{1}**H NMR** (500 MHz, CDCl$_3$) and ^{13}**C[^{1}H] NMR** (125 MHz, CDCl$_3$) of **(11d)**

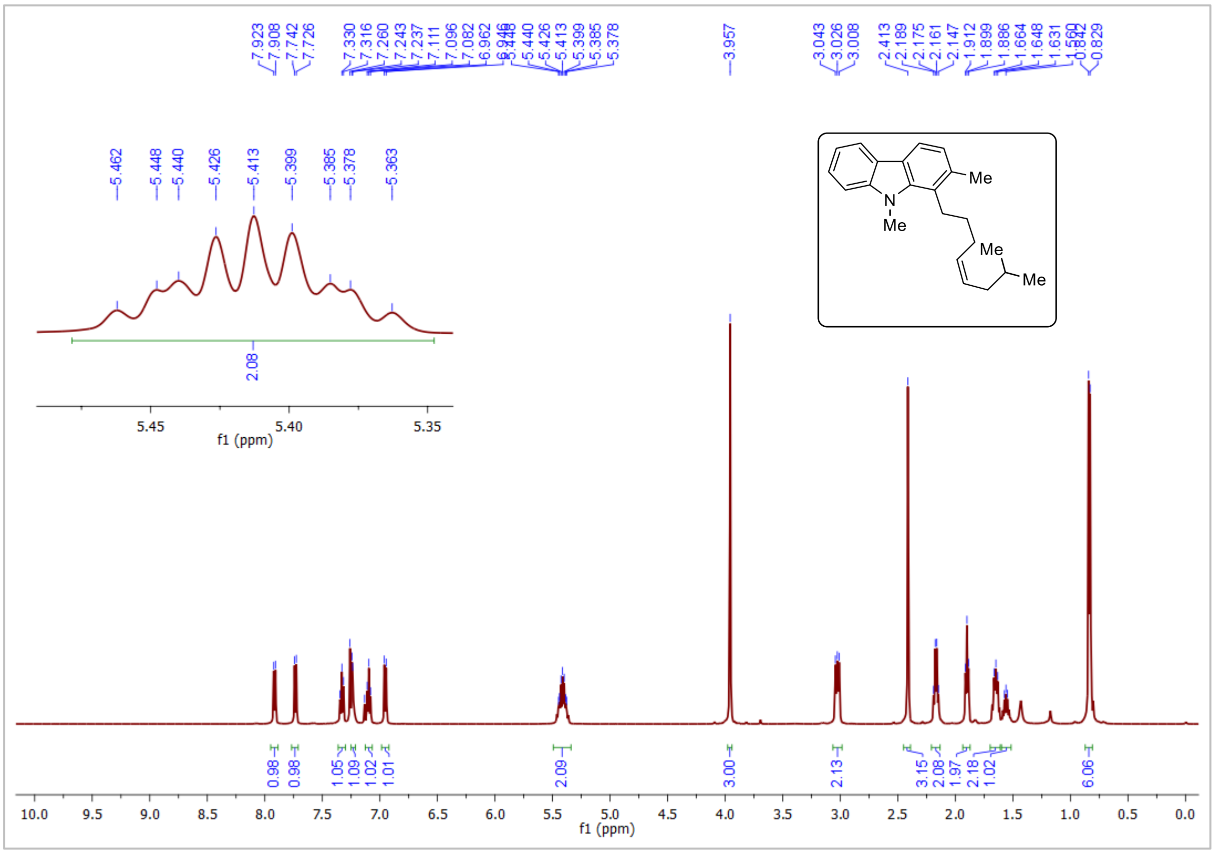

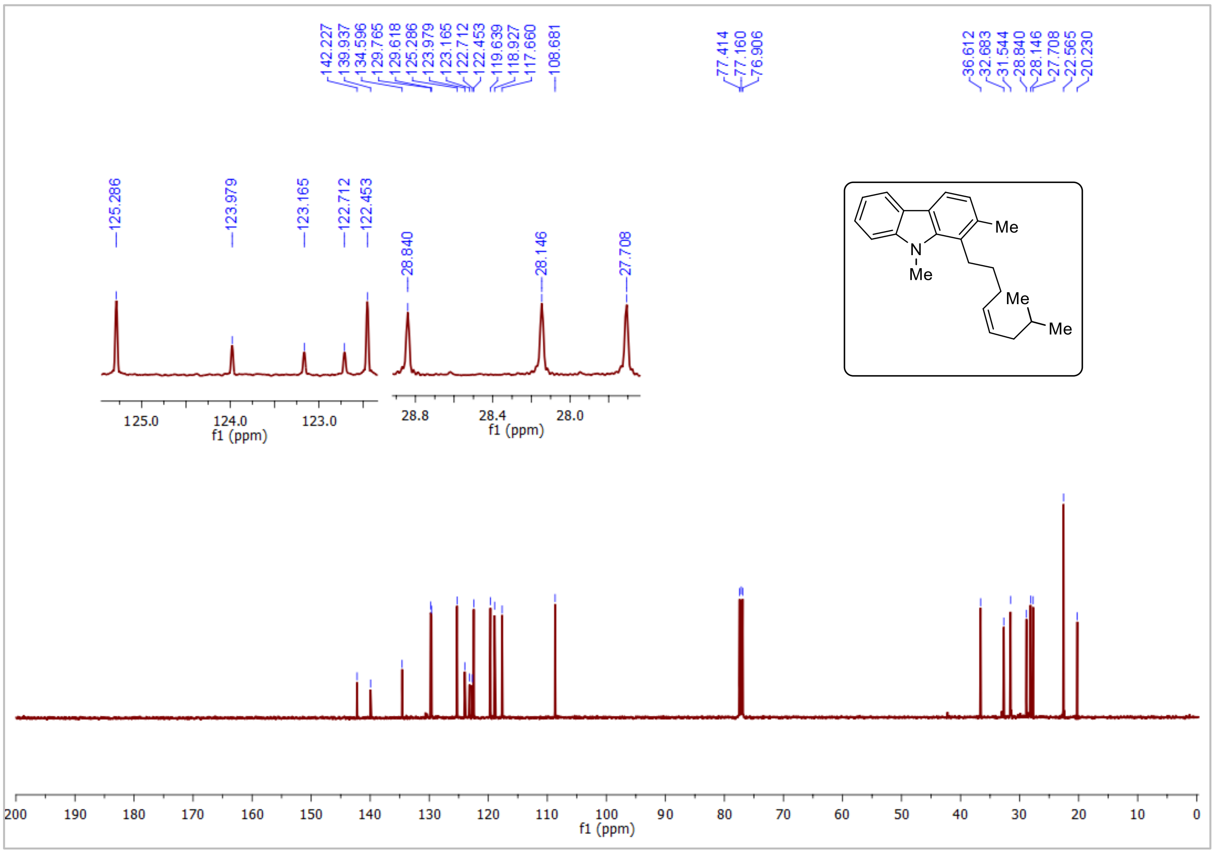

^{1}H NMR (500 MHz, $CDCl_3$) and **^{13}C[^{1}H] NMR** (125 MHz, $CDCl_3$) of **(12d)**

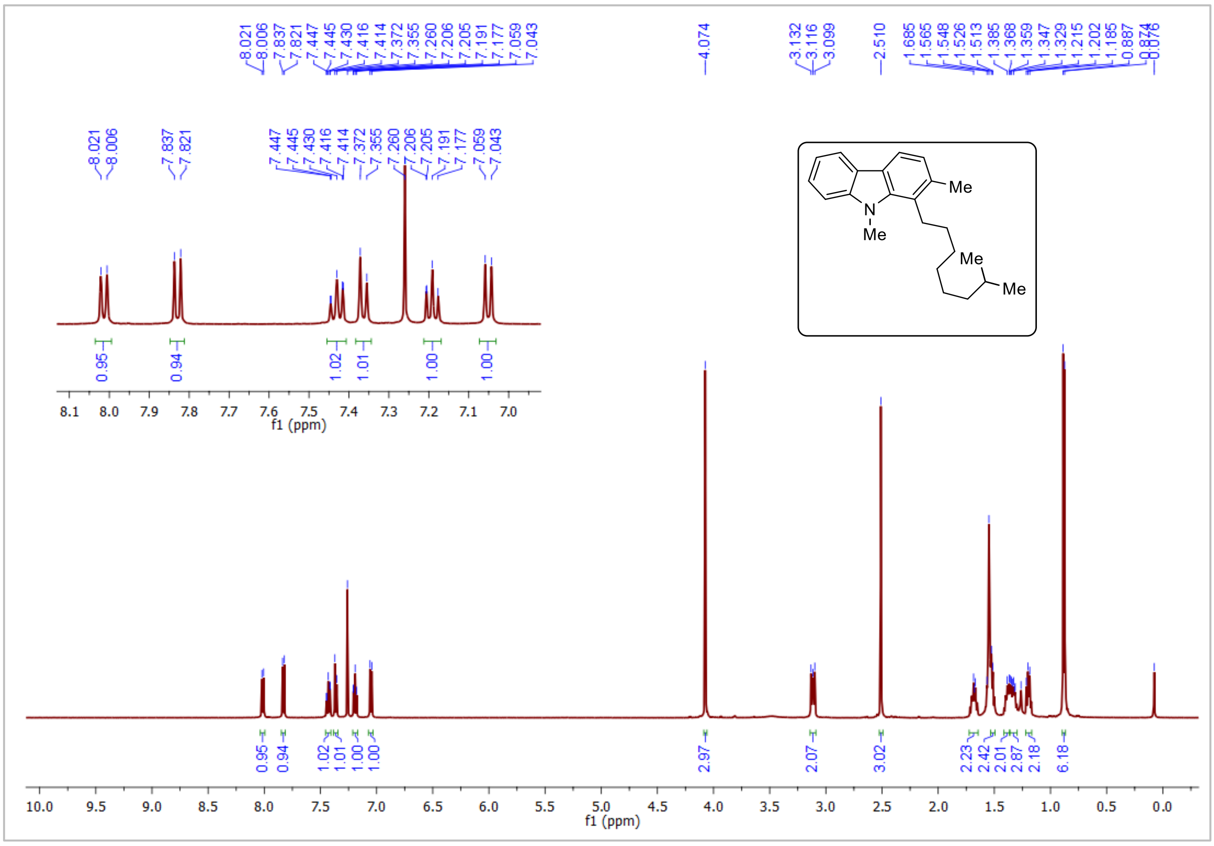

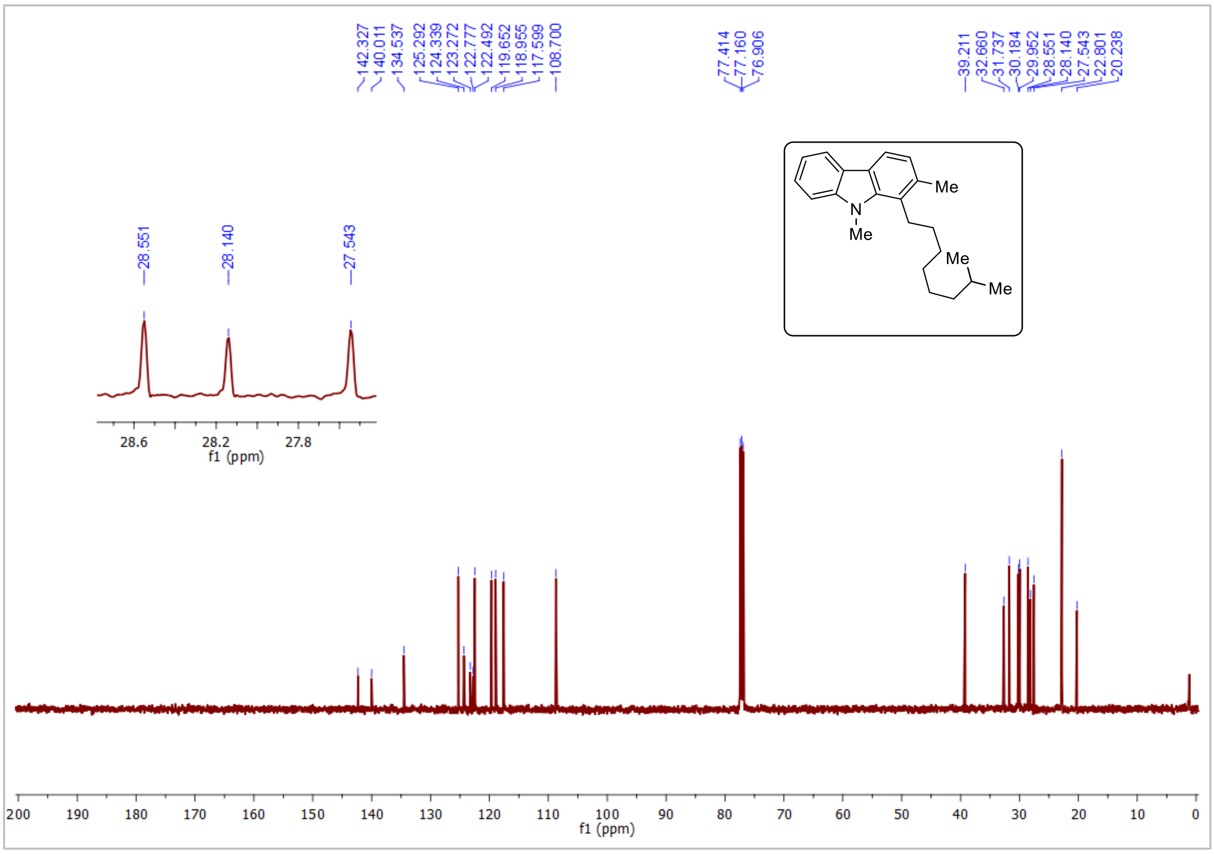

^{1}H NMR (500 MHz, CDCl$_3$) and ^{13}C[^{1}H] NMR (125 MHz, CDCl$_3$) of **(13d)**

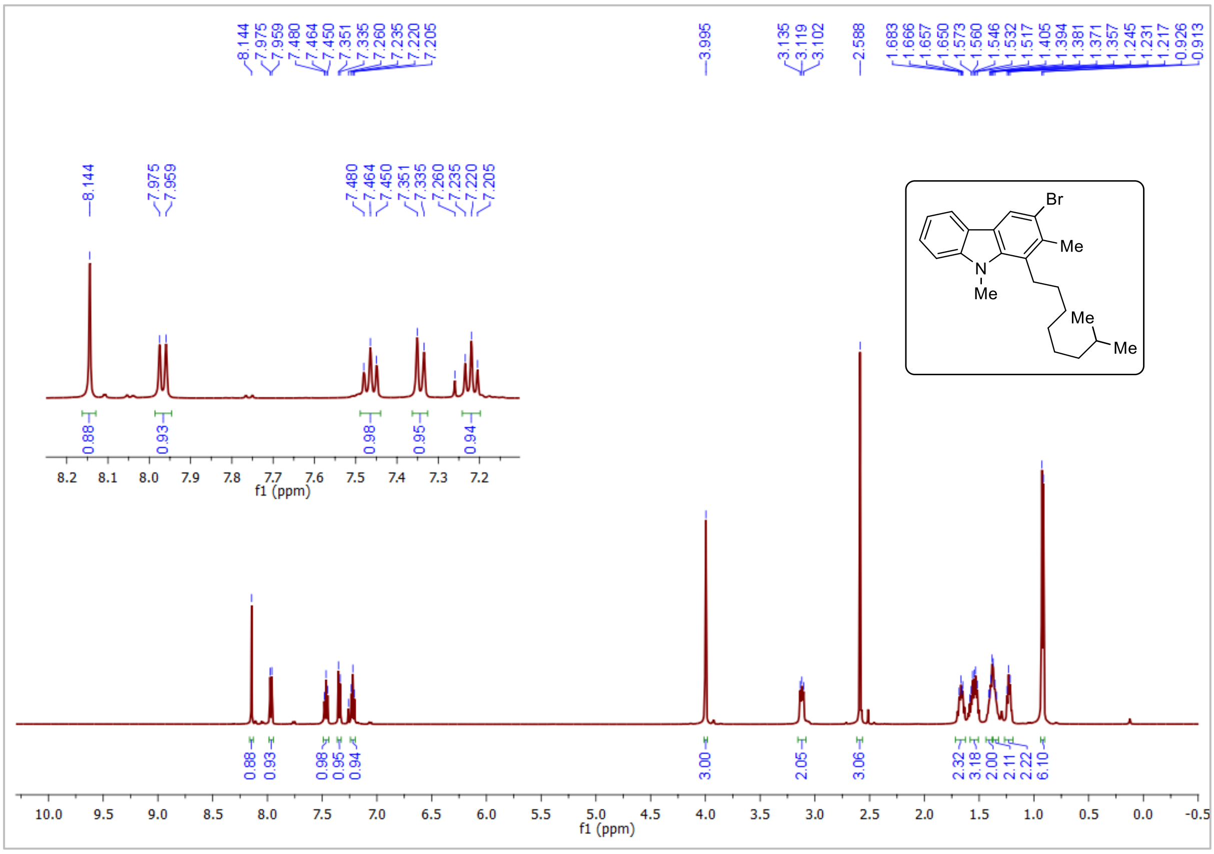

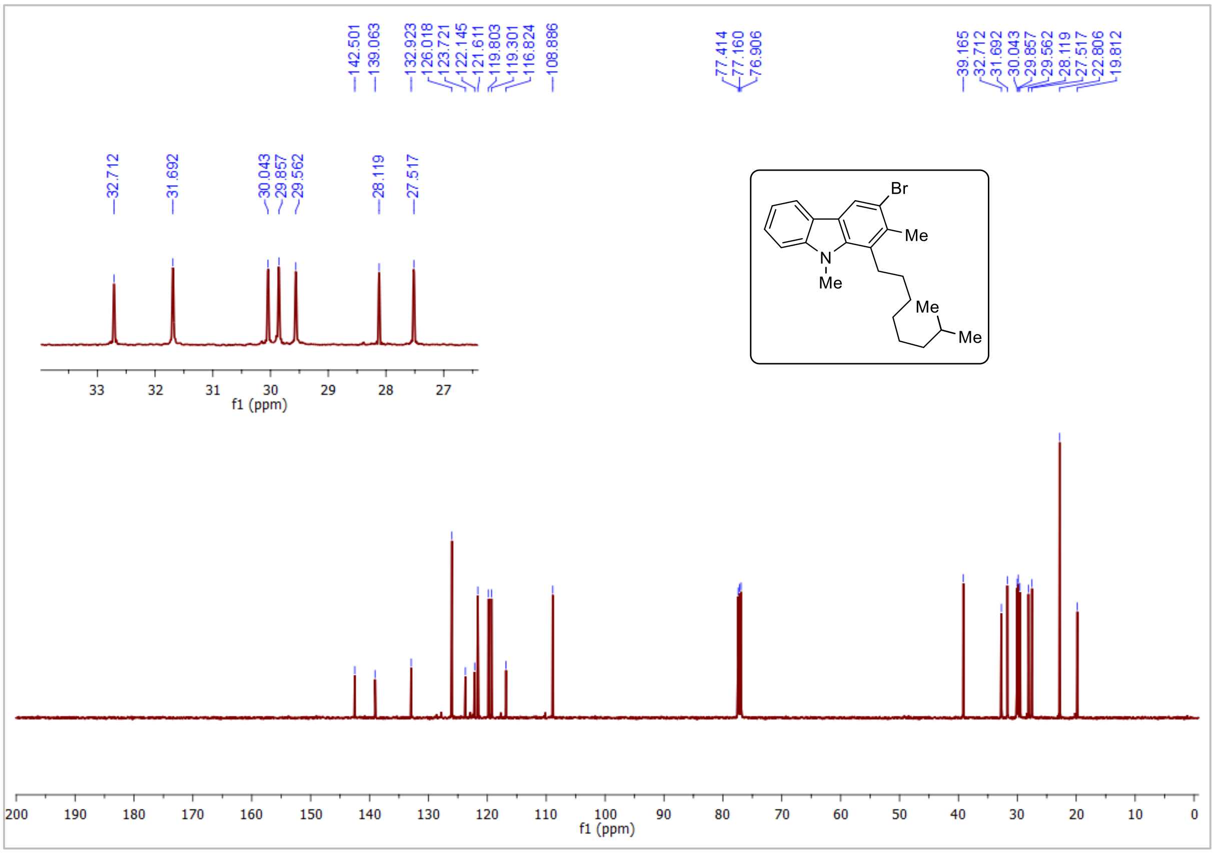

^{1}H NMR (400 MHz, $CDCl_3$) and **^{13}C[^{1}H] NMR** (100 MHz, $CDCl_3$) of **(14d)**

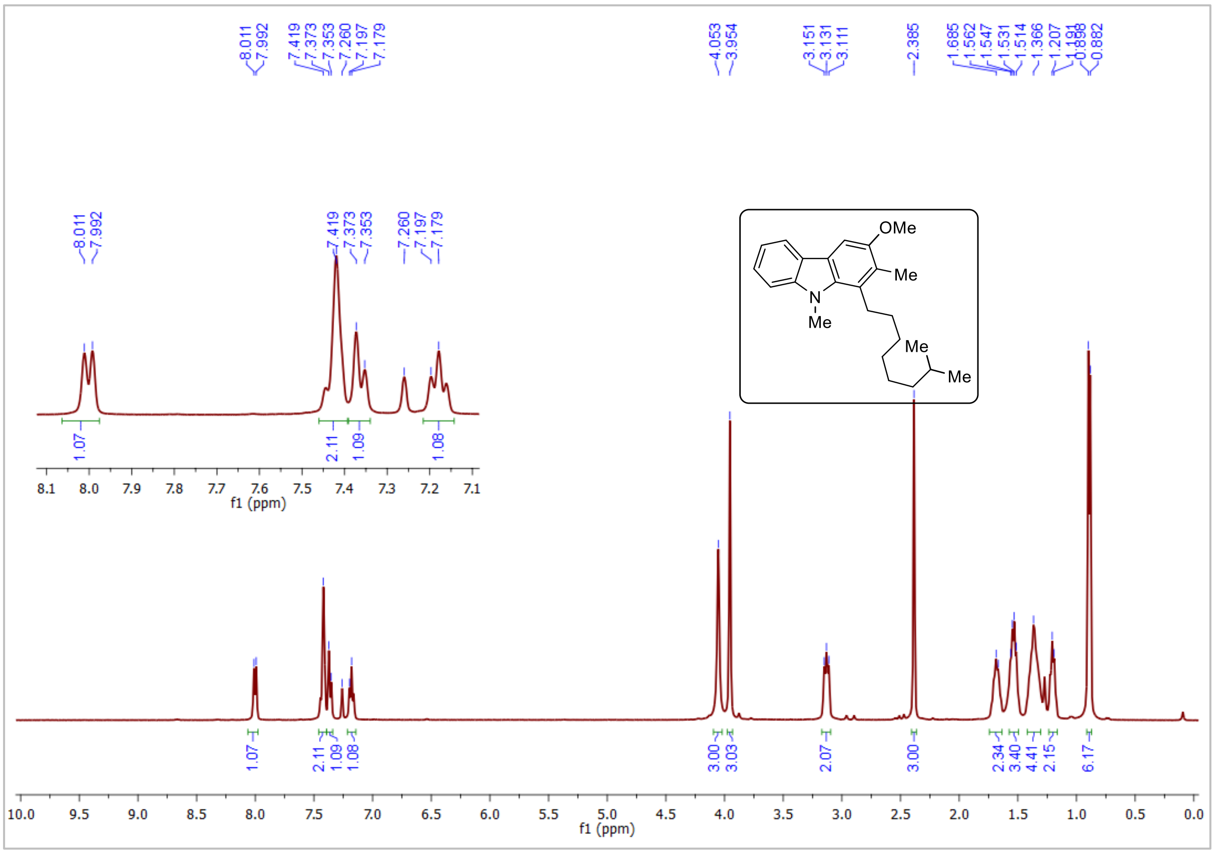

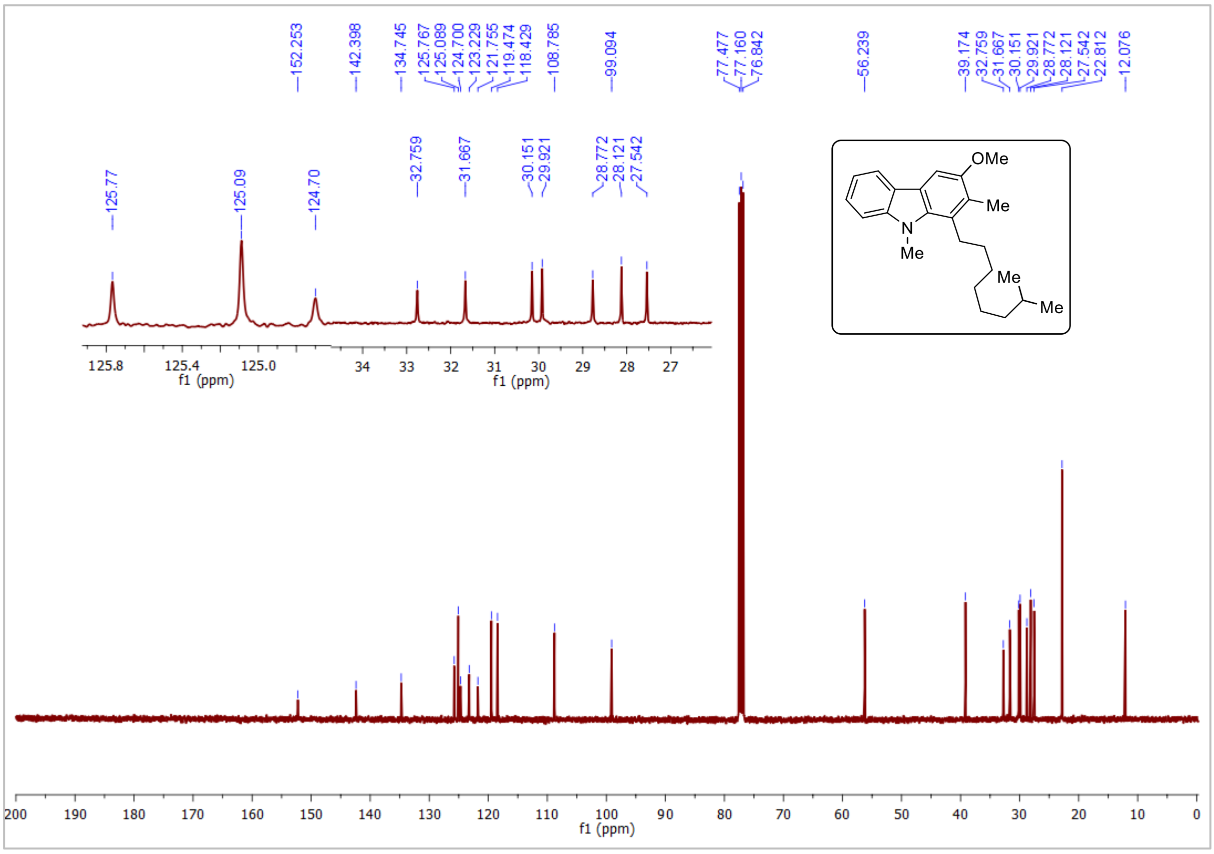

^{1}**H NMR** (400 MHz, $CDCl_3$) and ^{13}**C[^{1}H] NMR** (100 MHz, $CDCl_3$) of **(15d)**

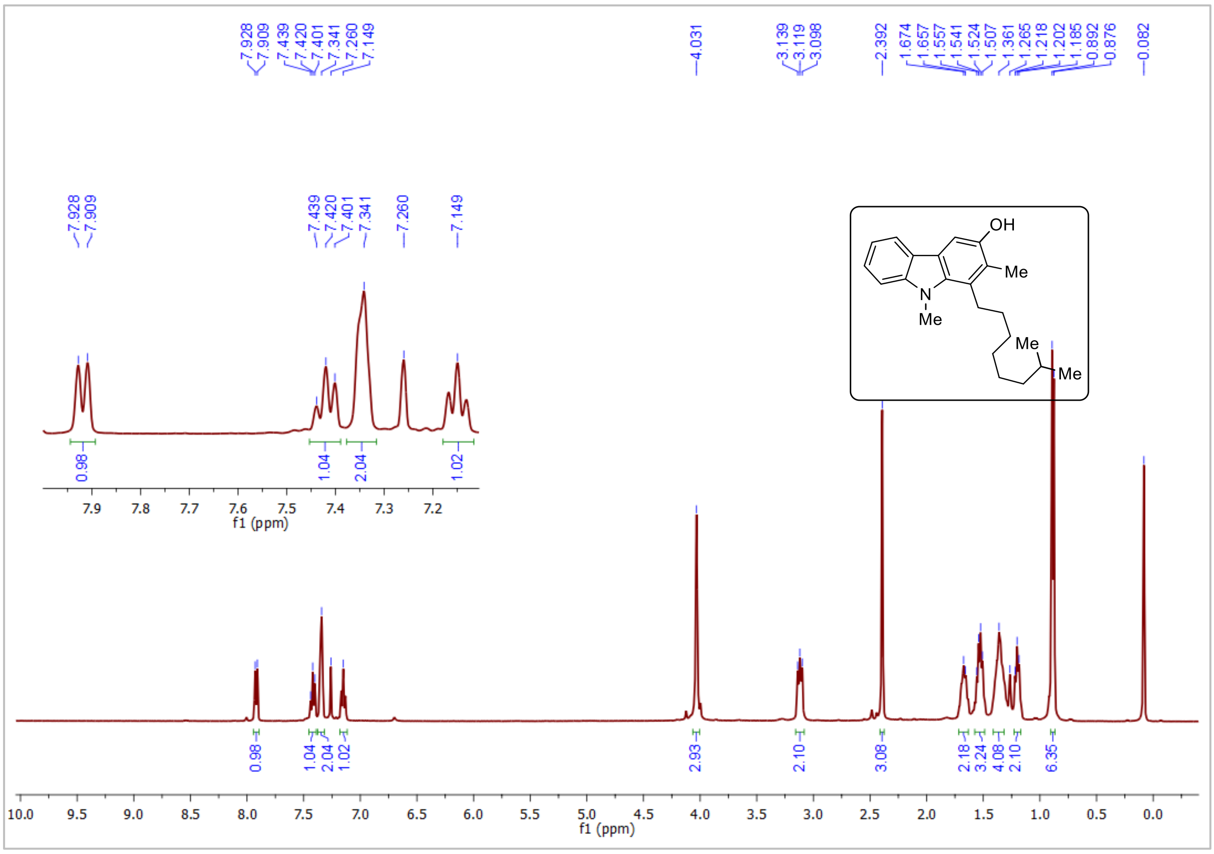

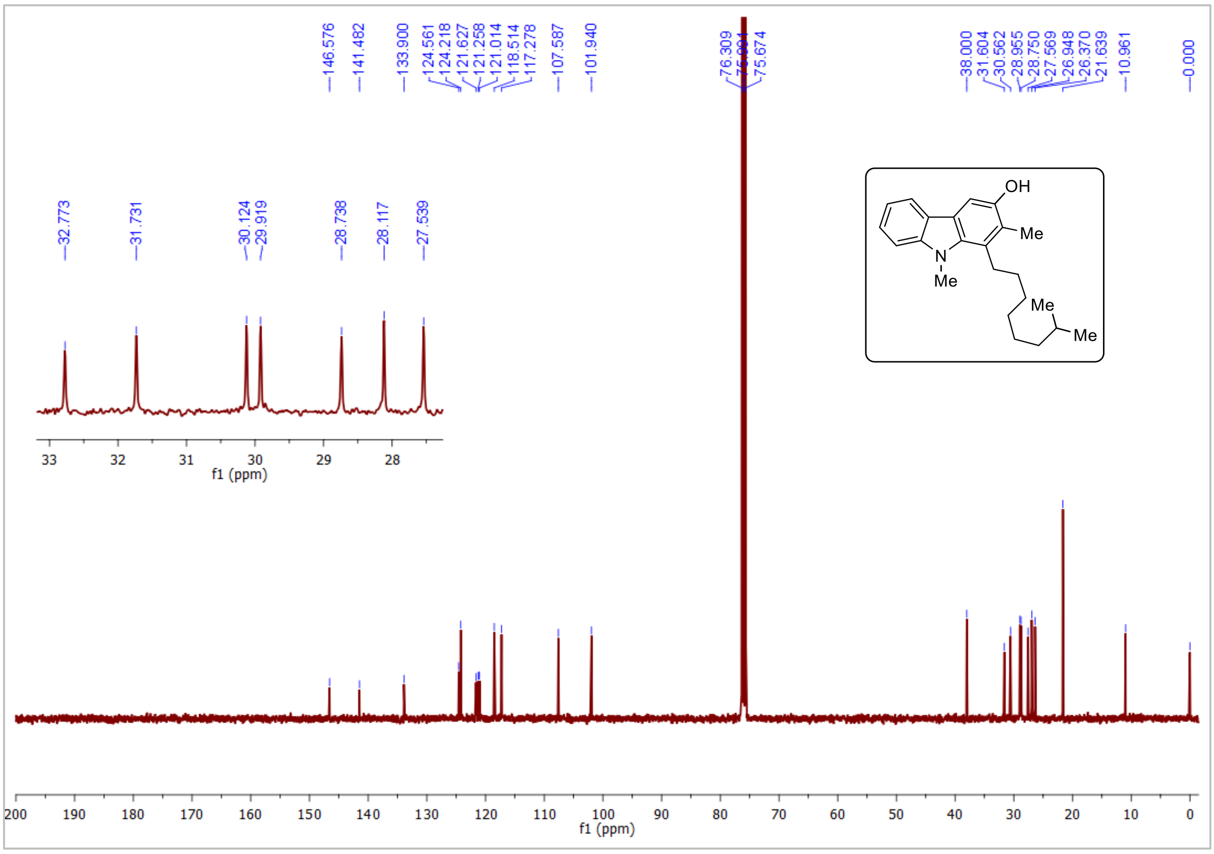

^{1}H NMR (400 MHz, $CDCl_3$) and ^{13}C[^{1}H] NMR (100 MHz, $CDCl_3$) of (6)

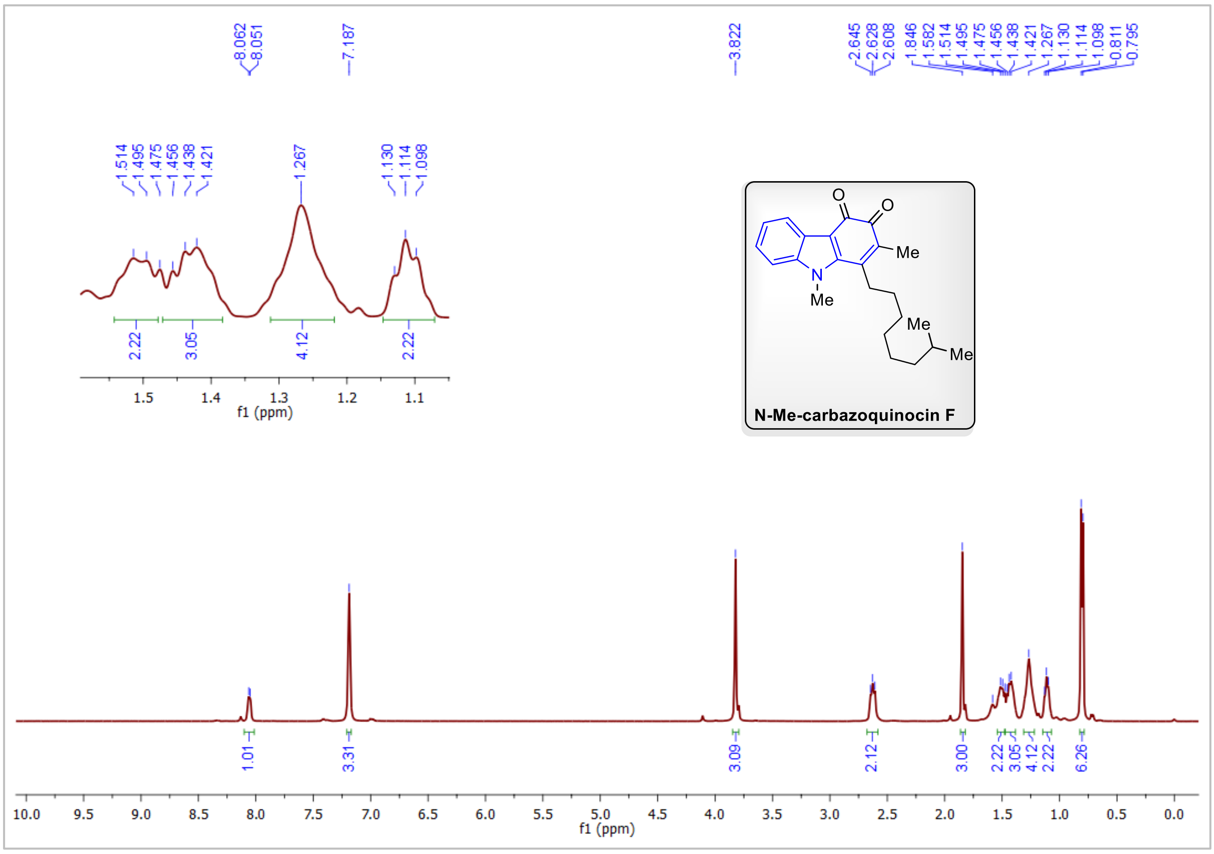

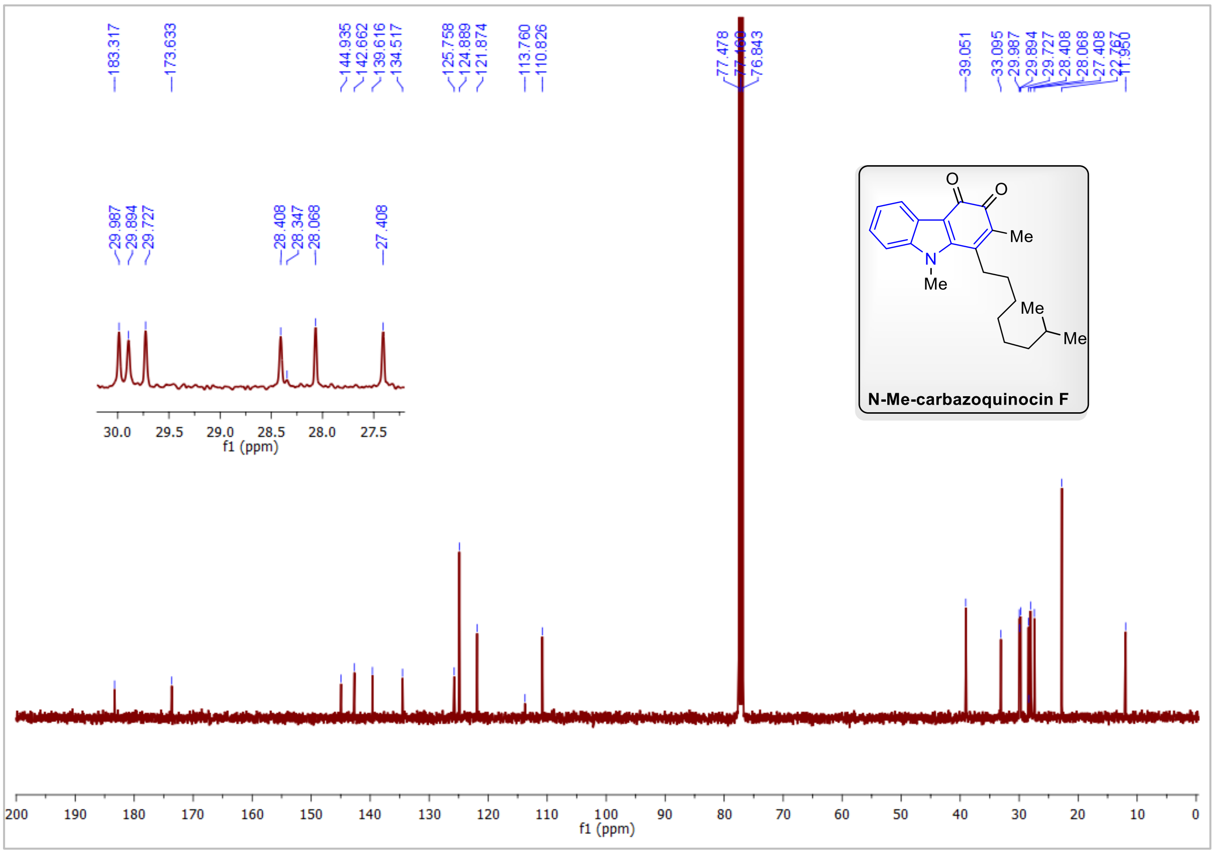

^{1}H NMR (400 MHz, $CDCl_3$) and ^{13}C[^{1}H] NMR (100 MHz, $CDCl_3$) of (17)

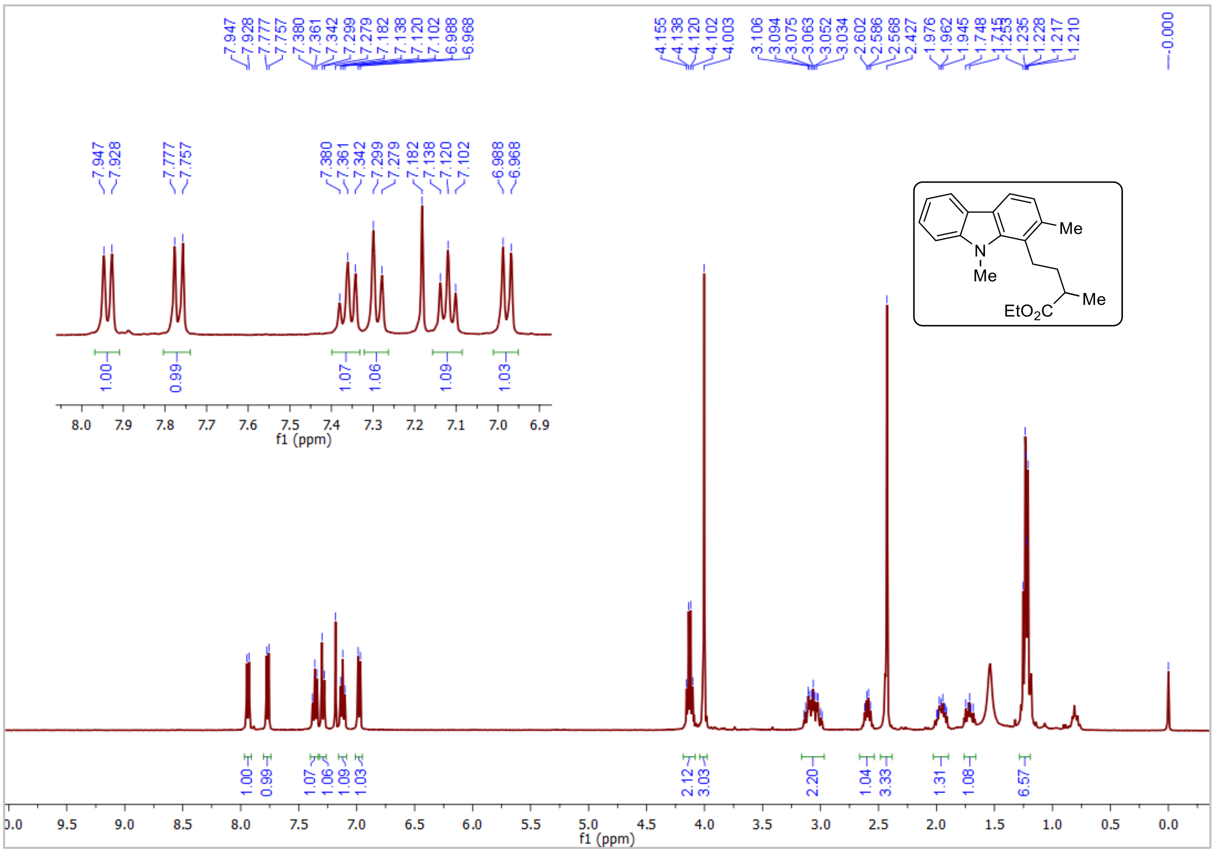

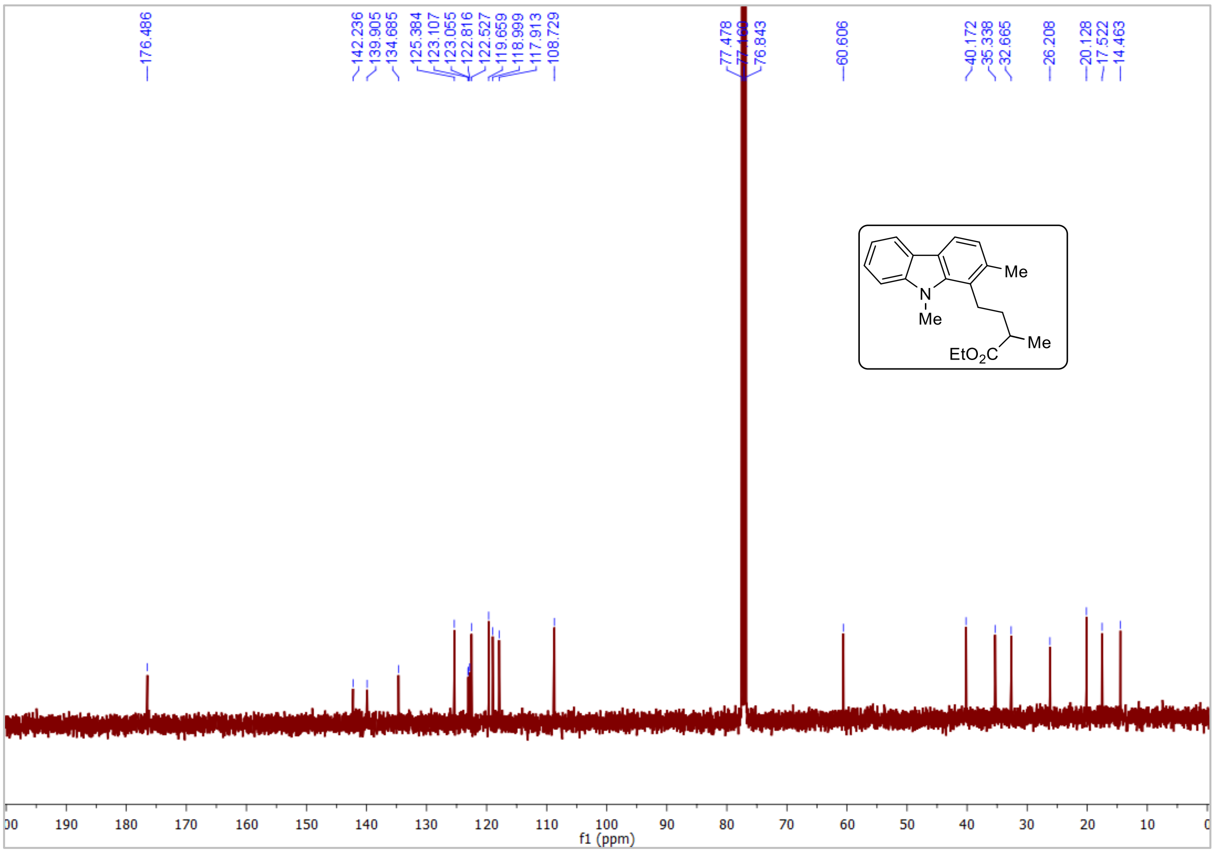

^{1}H NMR (500 MHz, $CDCl_3$) and **^{13}C[^{1}H] NMR** (125 MHz, $CDCl_3$) of **(18)**

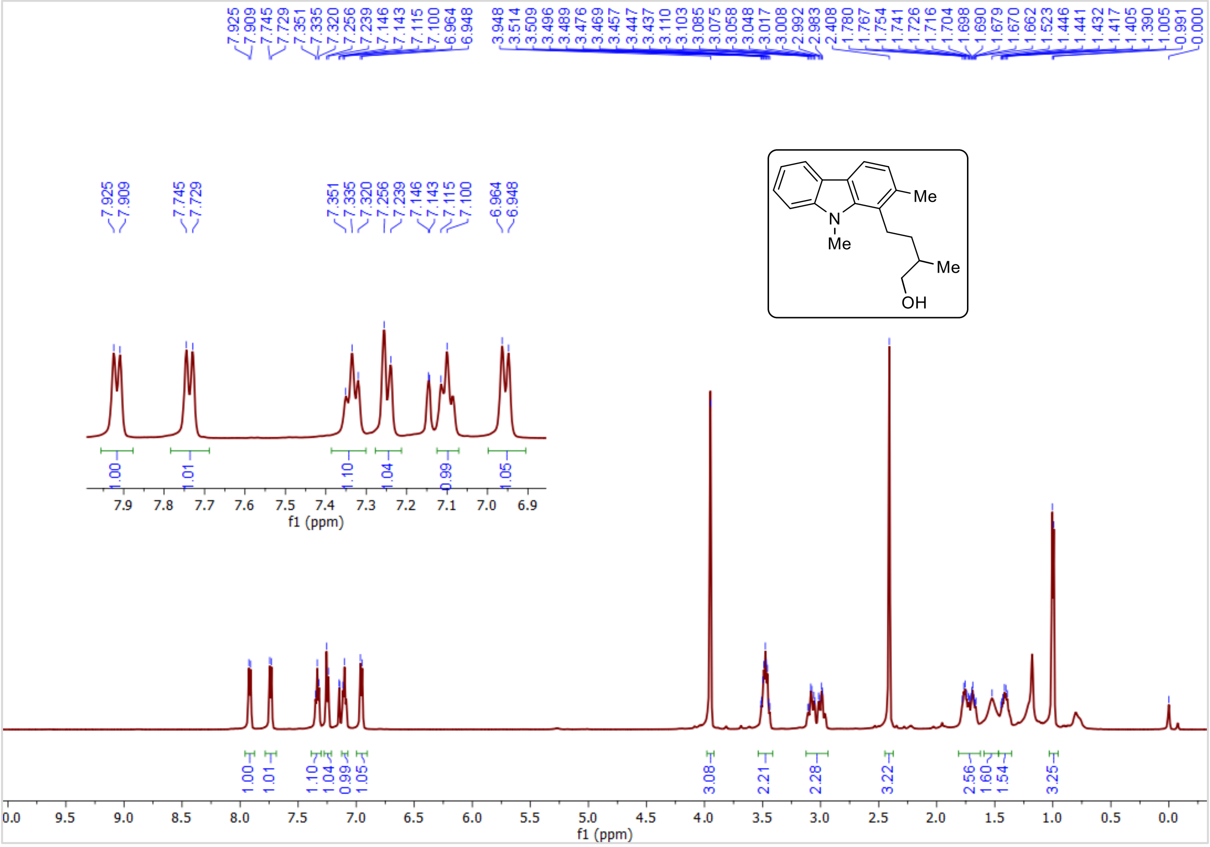

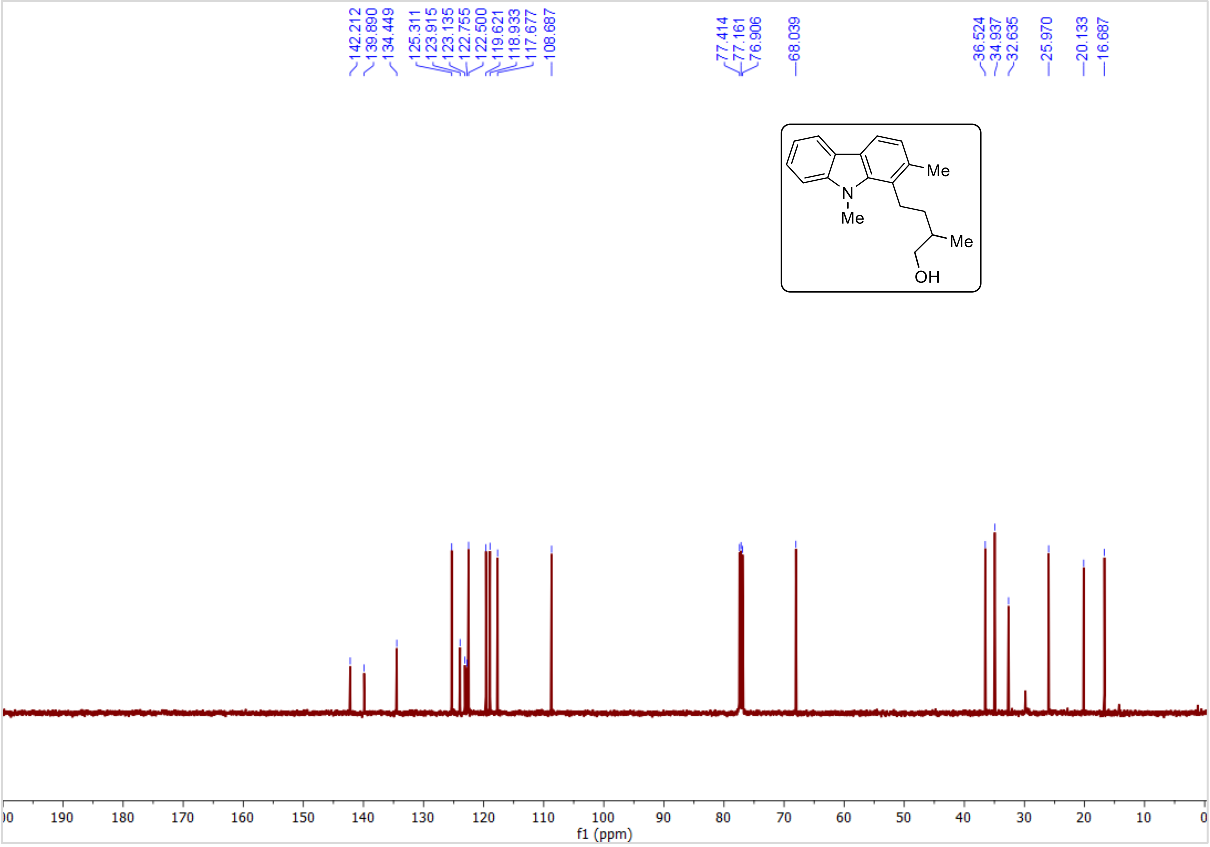

^{1}**H NMR** (400 MHz, $CDCl_3$) and ^{13}**C[^{1}H] NMR** (100 MHz, $CDCl_3$) of **(19)**

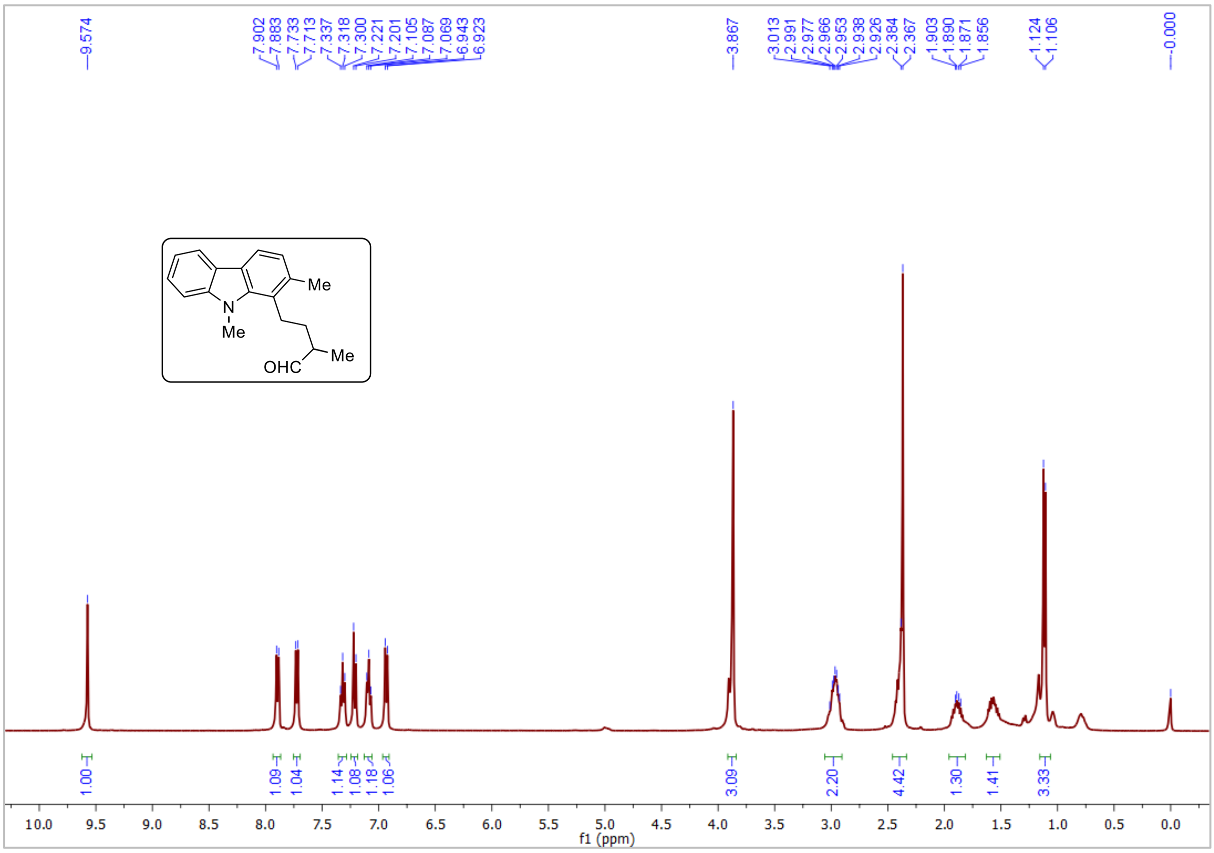

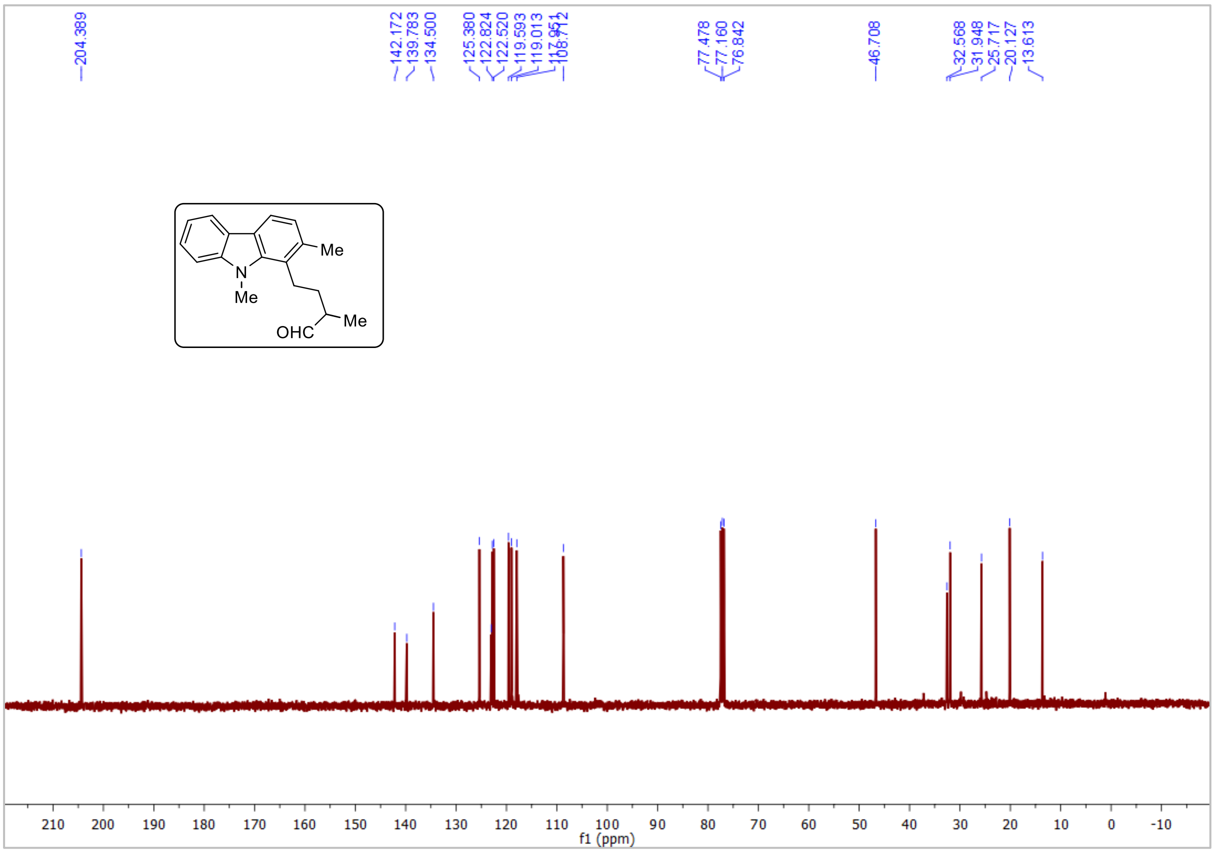

¹H NMR (400 MHz, $CDCl_3$) and **¹³C[¹H] NMR** (100 MHz, $CDCl_3$) of **(20)**

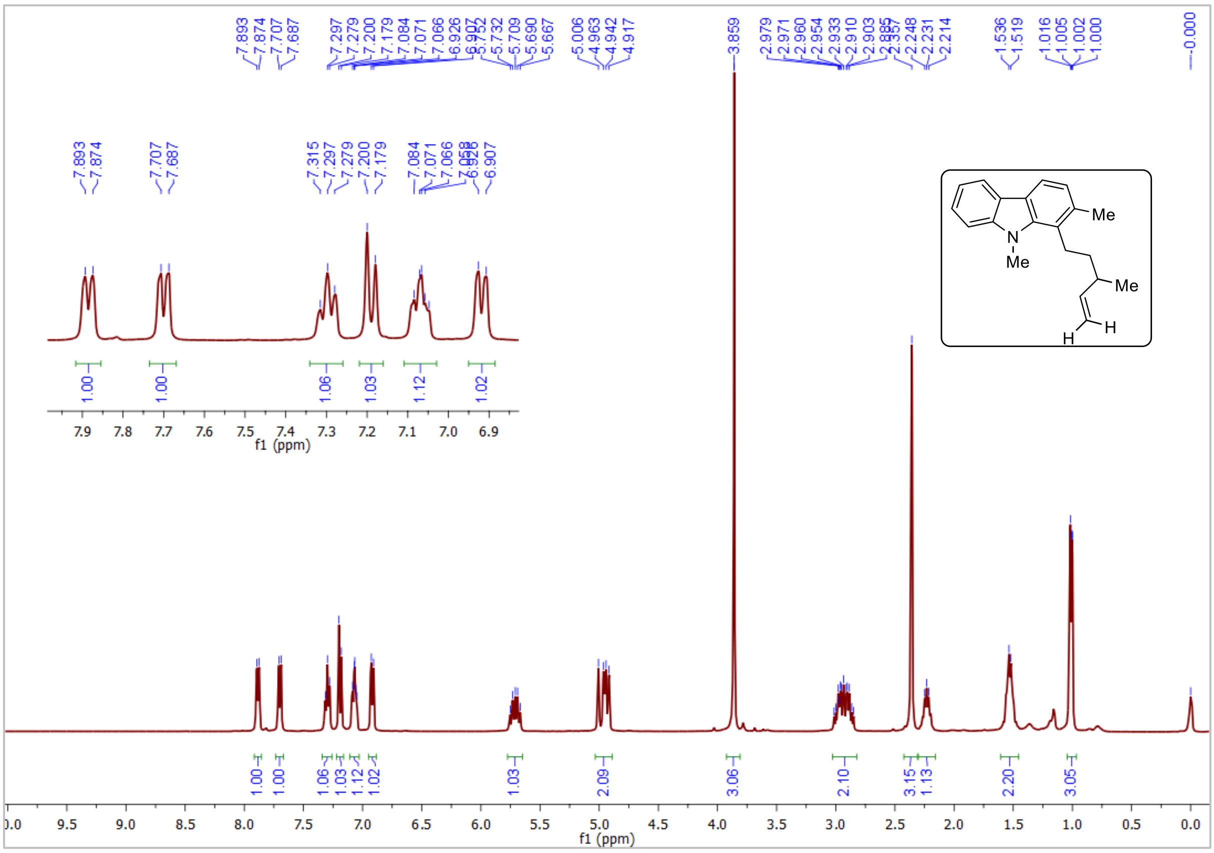

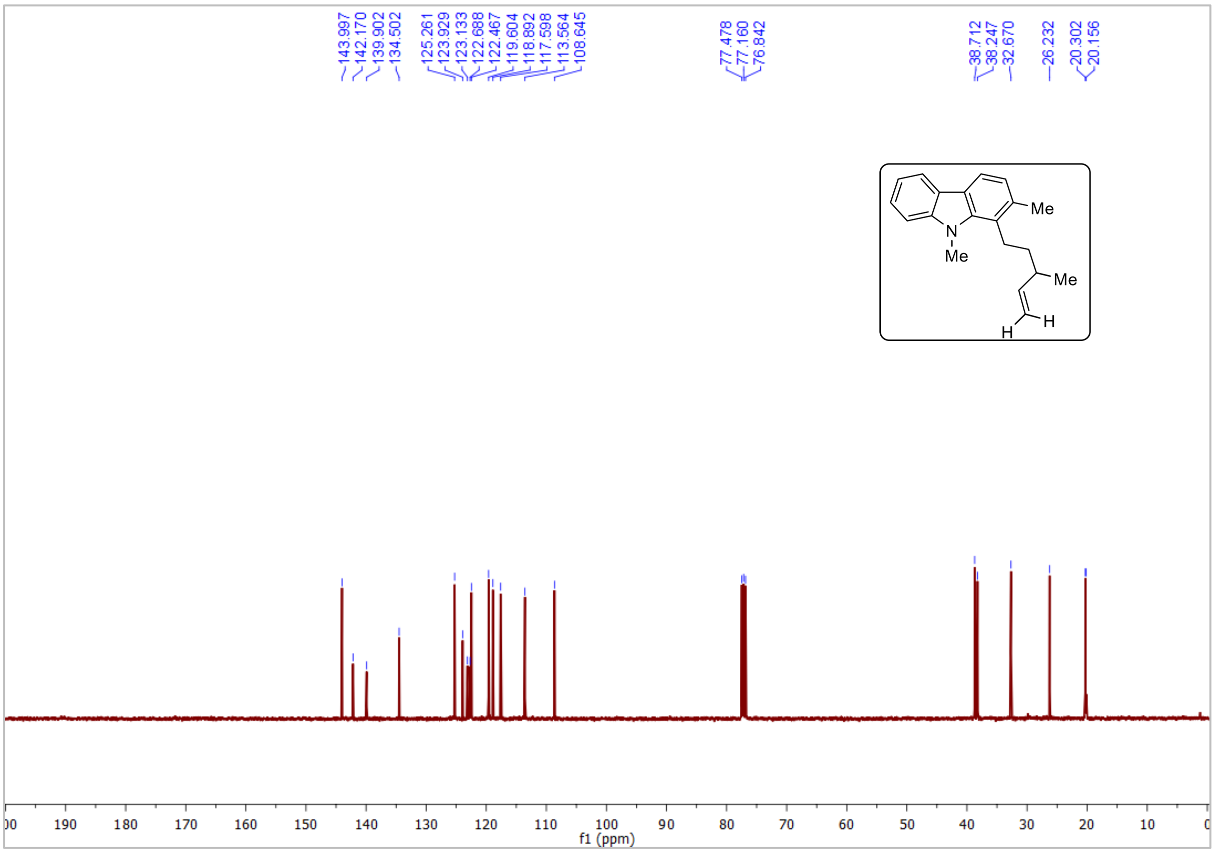

^{1}H NMR (400 MHz, $CDCl_3$) and **^{13}C[^{1}H] NMR** (100 MHz, $CDCl_3$) of **(21)**

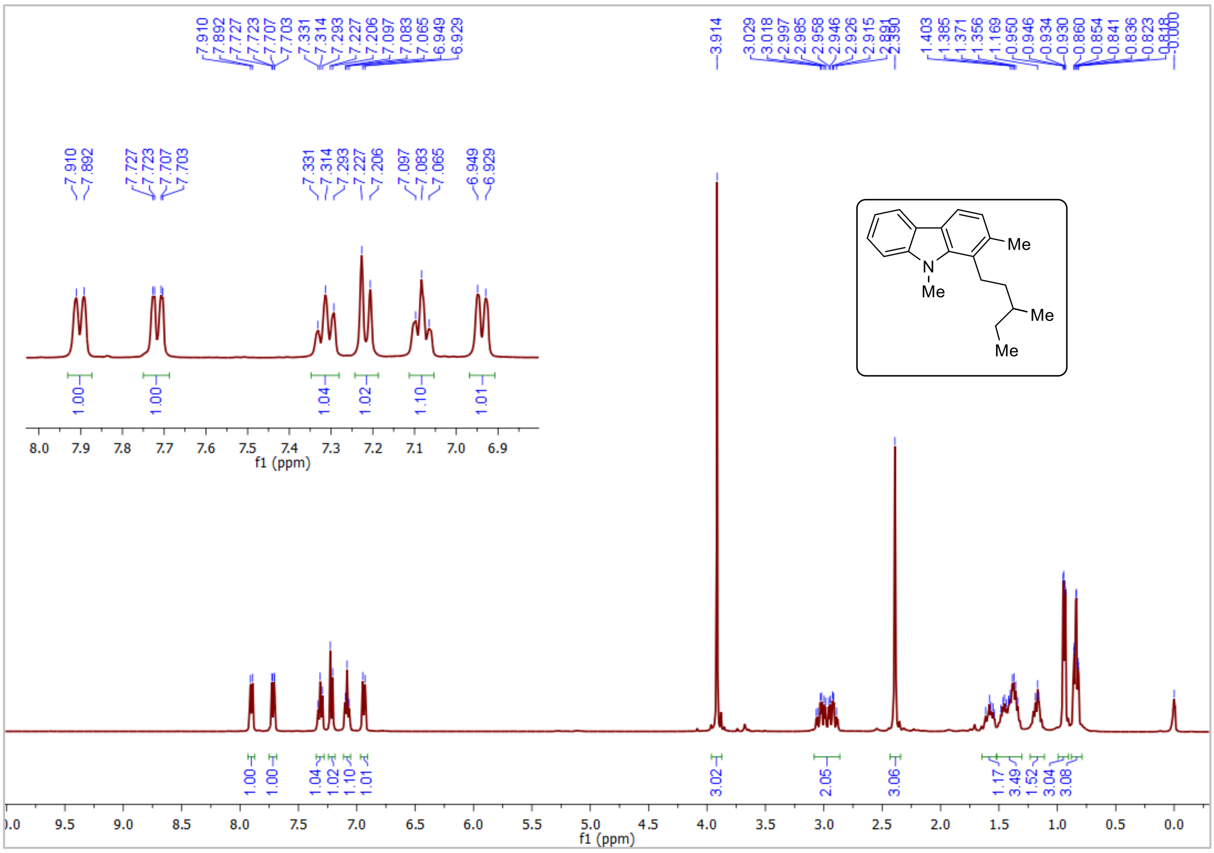

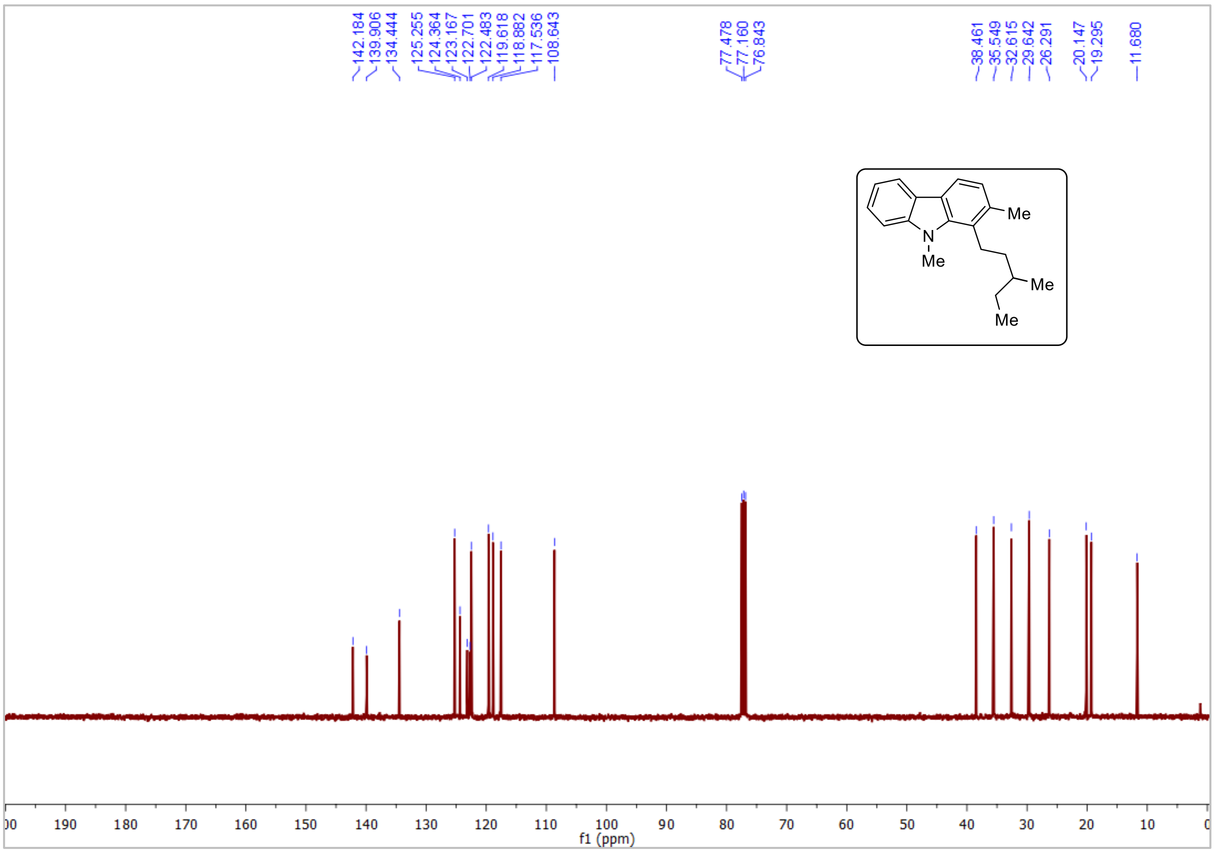

^{1}H NMR (400 MHz, $CDCl_3$) and **^{13}C[^{1}H] NMR** (100 MHz, $CDCl_3$) of **(22)**

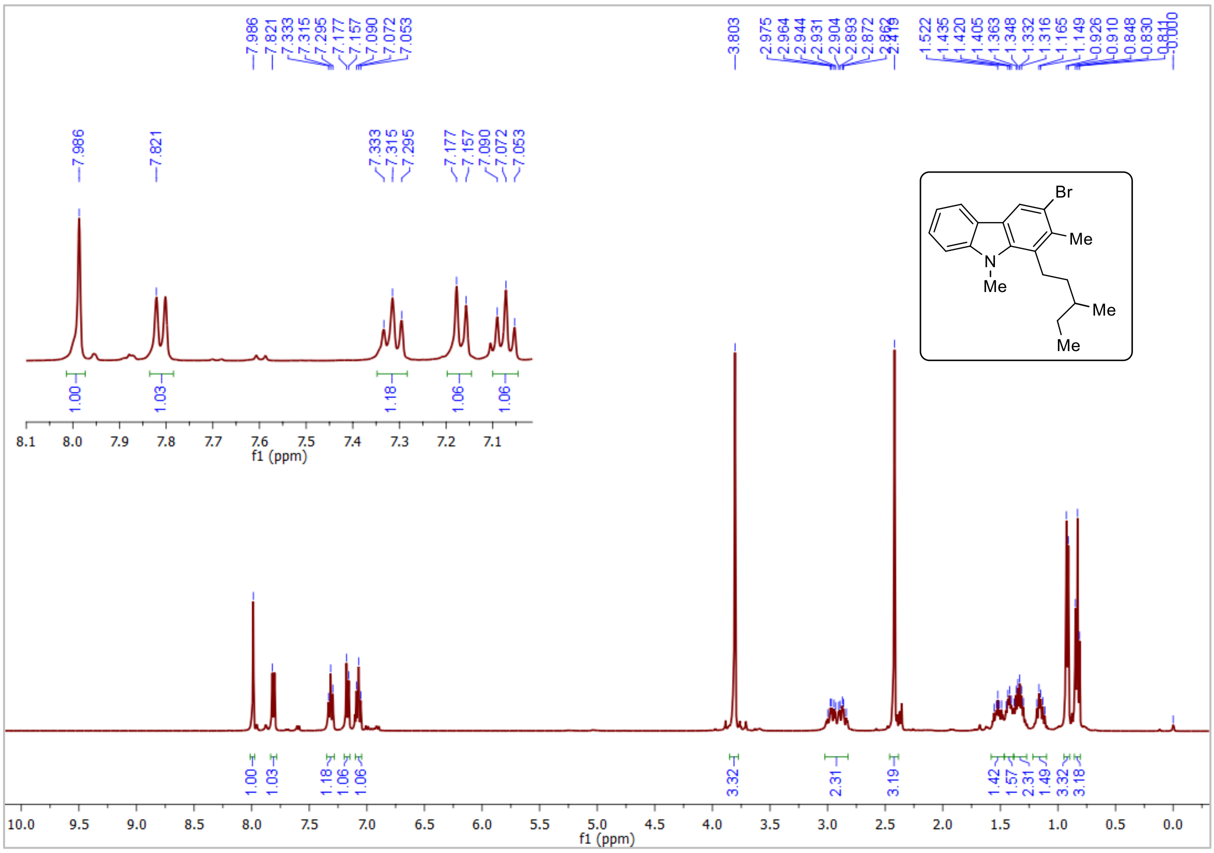

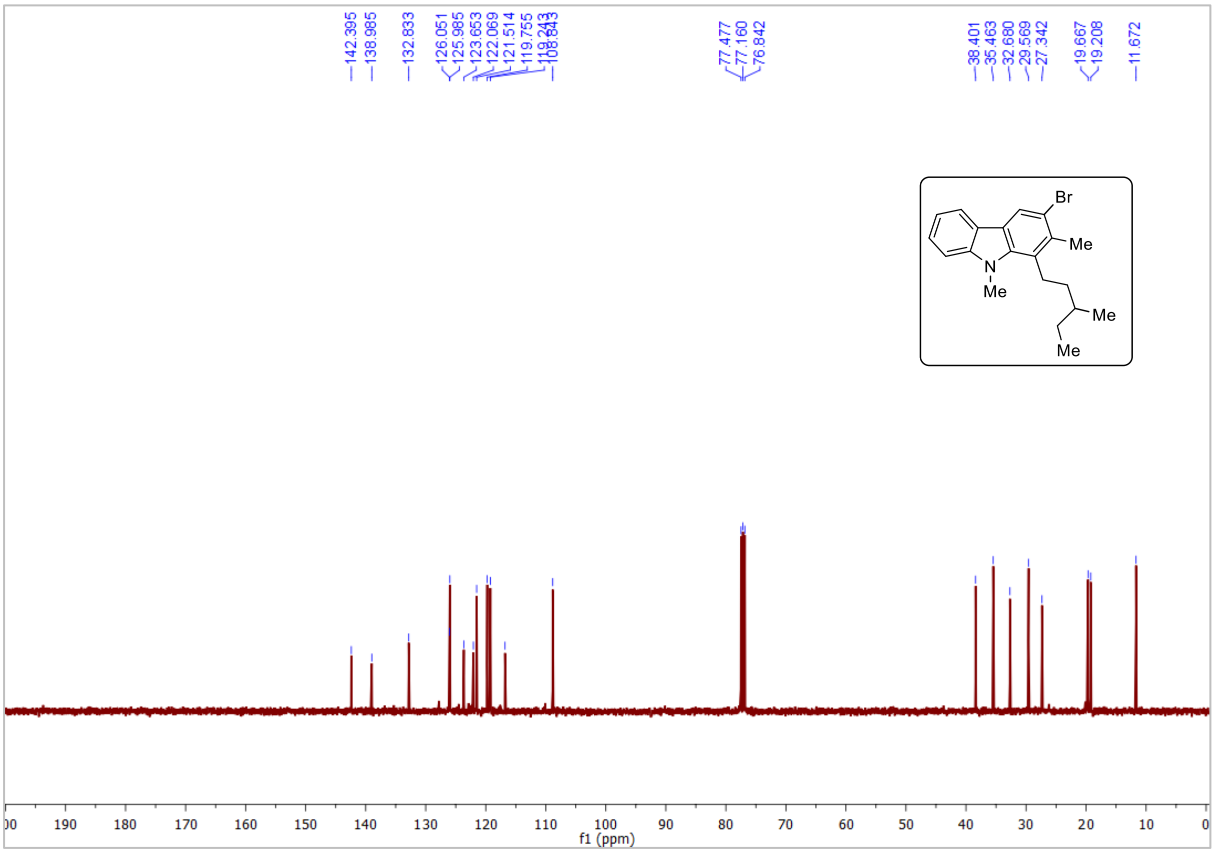

^{1}**H NMR** (400 MHz, CDCl$_3$) and ^{13}**C[^{1}H] NMR** (100 MHz, CDCl$_3$) of **(23)**

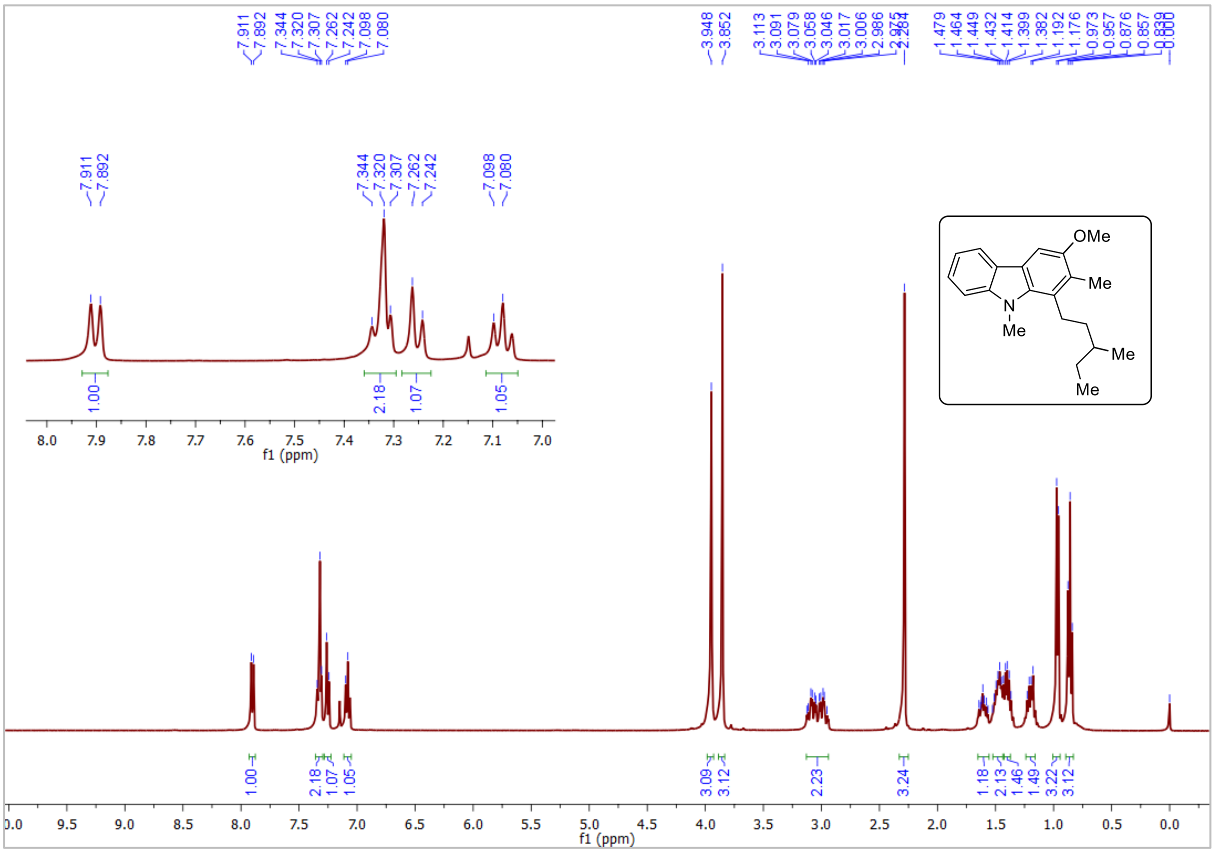

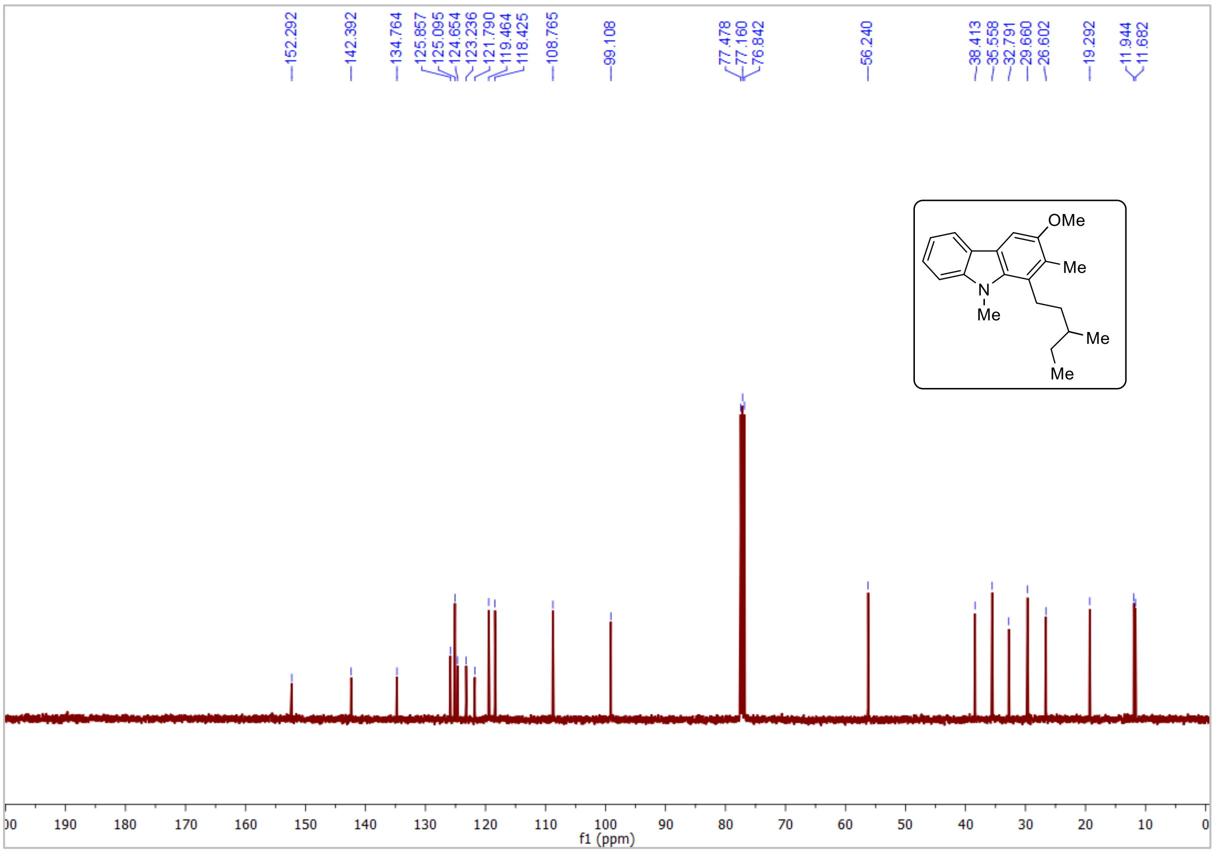

¹H NMR (500 MHz, $CDCl_3$) and **¹³C[¹H] NMR** (125 MHz, $CDCl_3$) of **(24)**

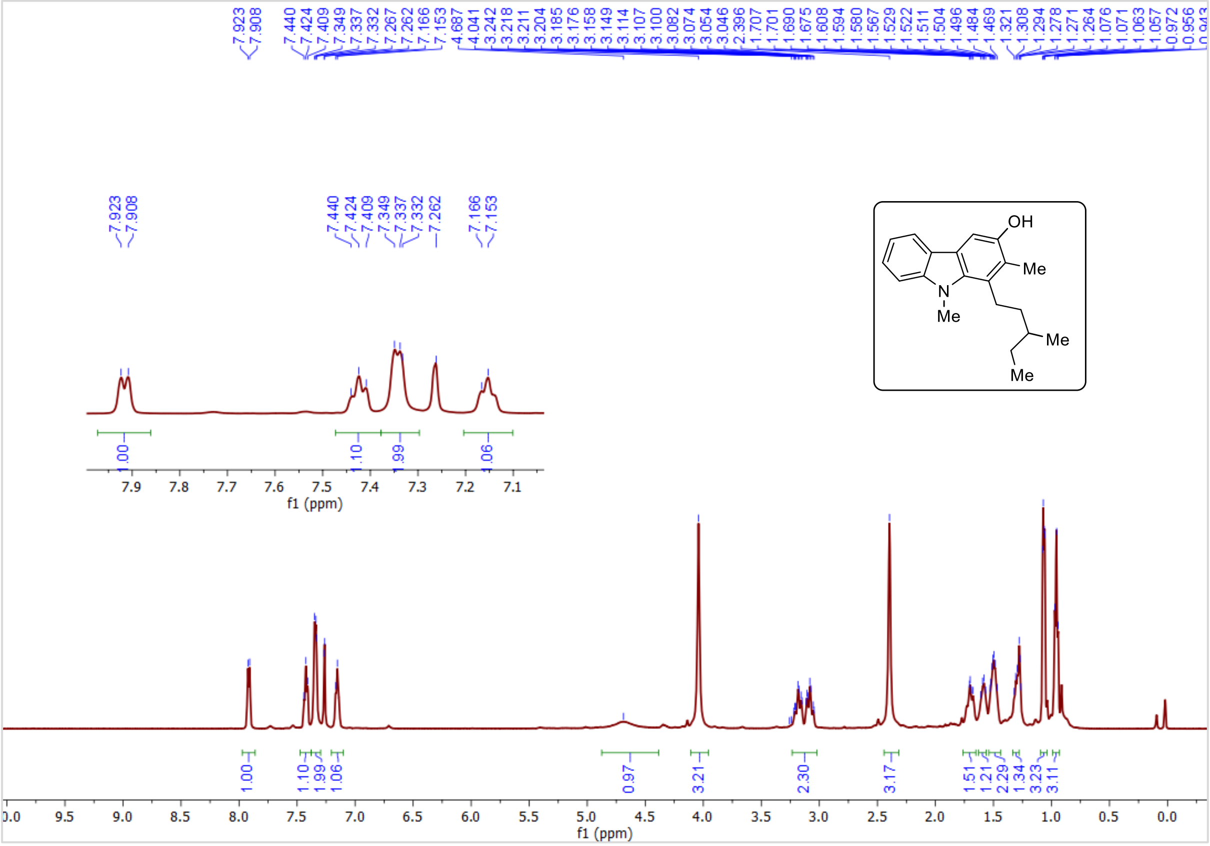

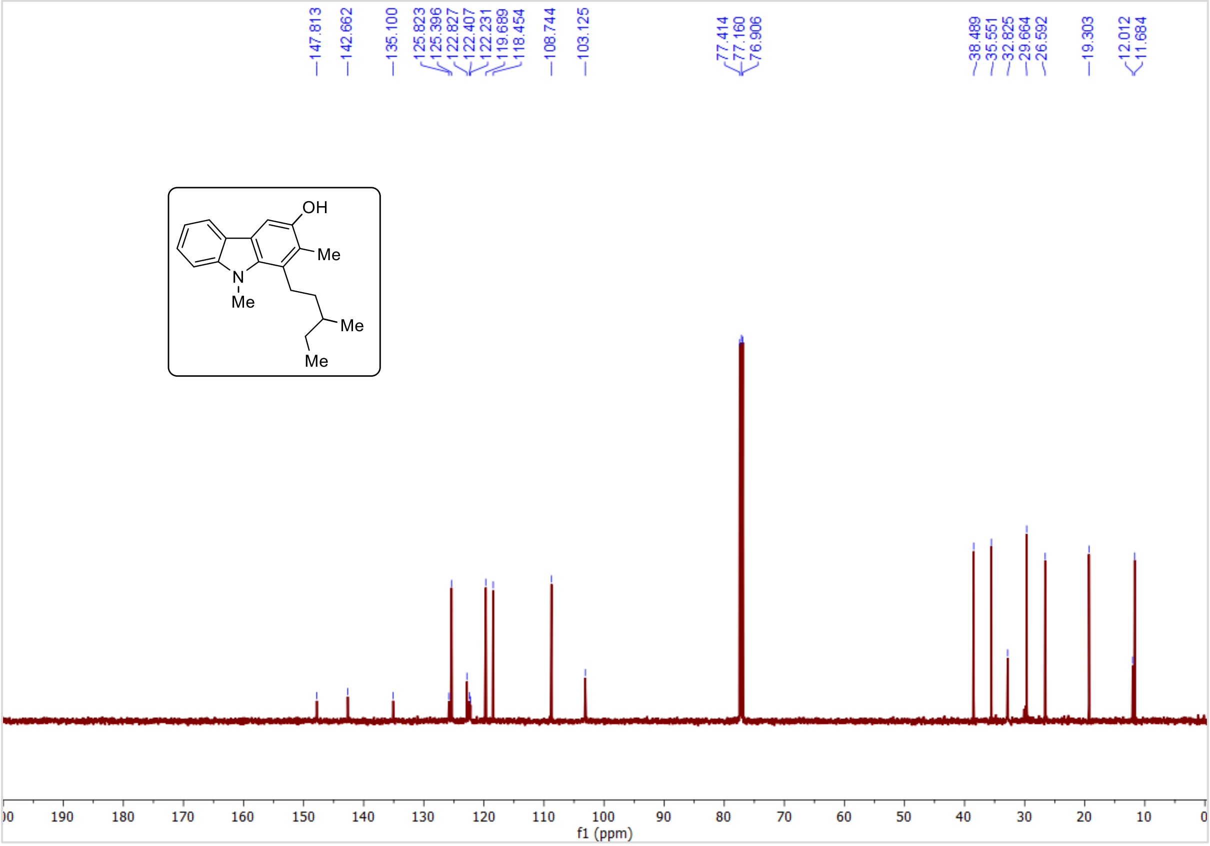

^{1}H NMR (400 MHz, $CDCl_3$) and **$^{13}C[^{1}H]$ NMR** (100 MHz, $CDCl_3$) of **(7)**

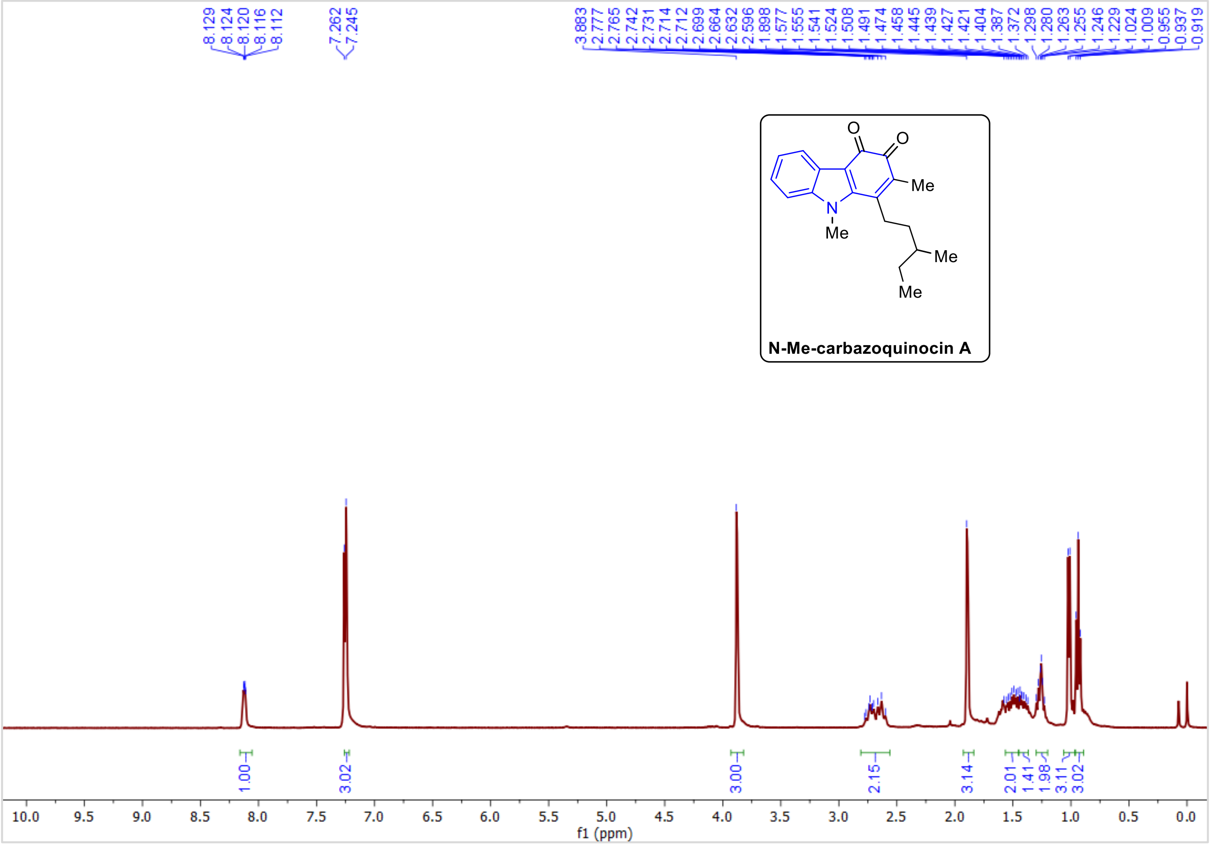

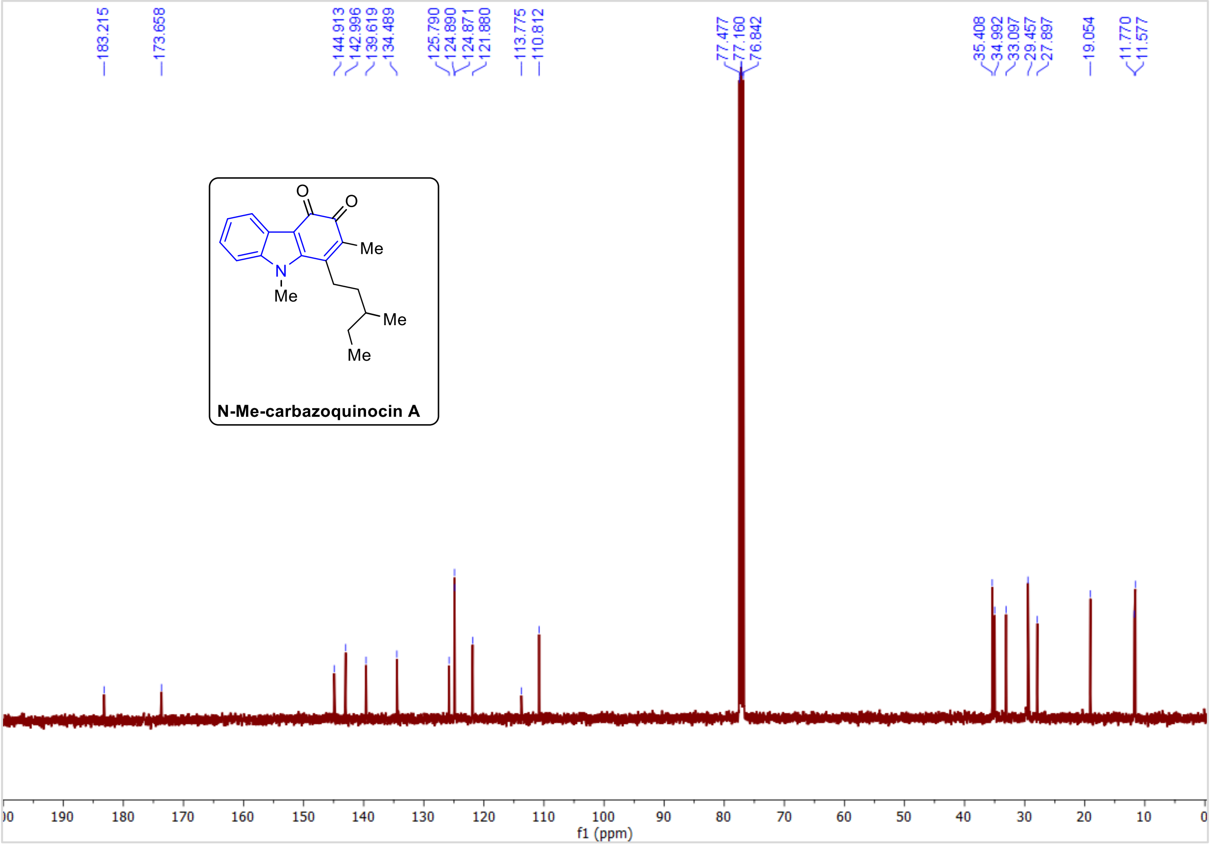

^{1}H NMR (400 MHz, $CDCl_3$) and **^{13}C[^{1}H] NMR** (100 MHz, $CDCl_3$) of **(S_1)**

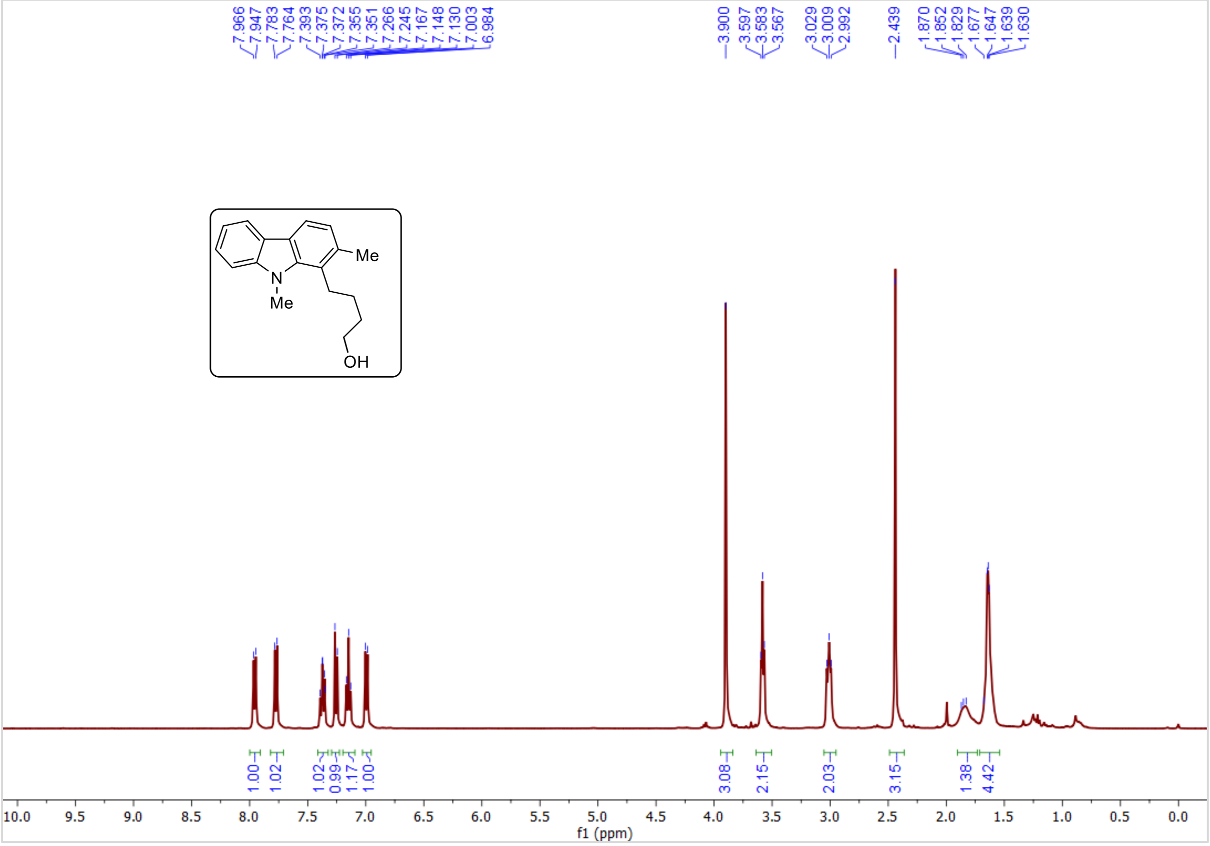

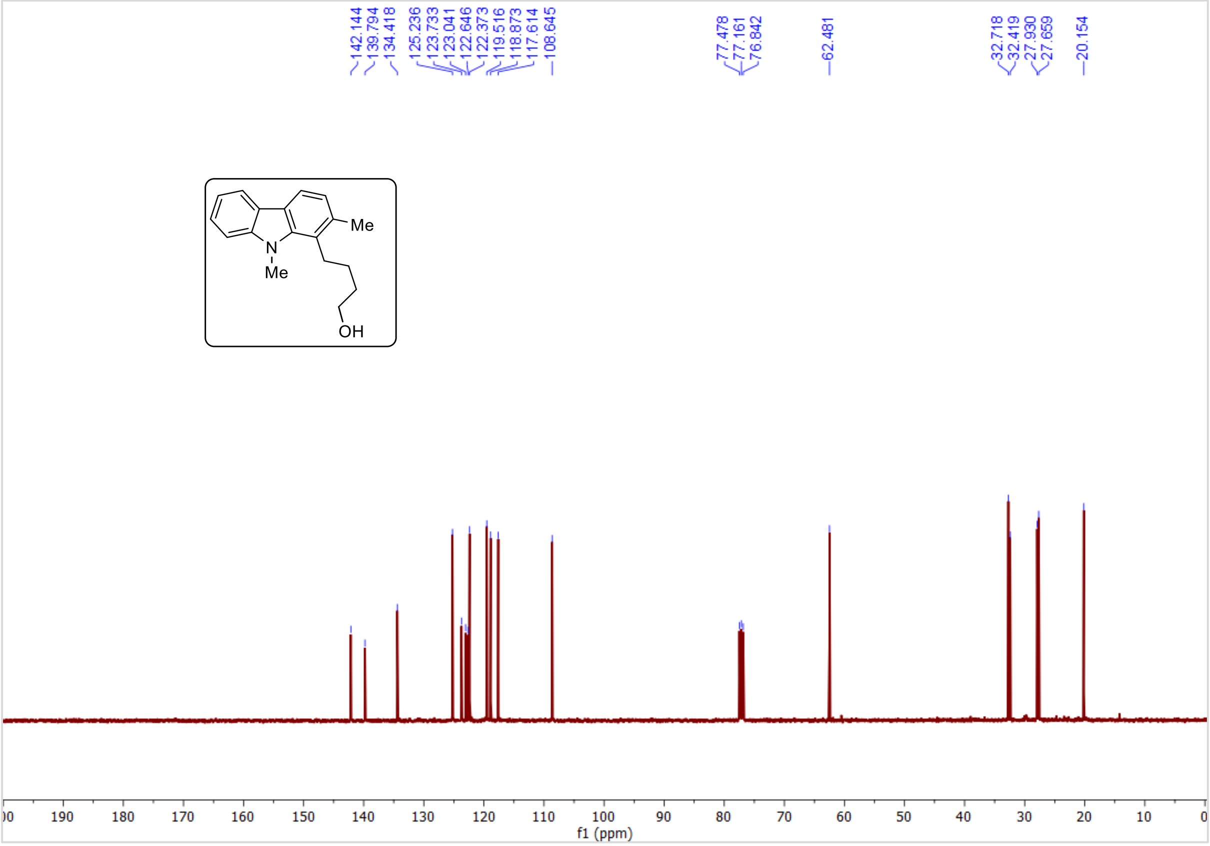

Supplement: RA-014-D4RA05347H-s001 [file RA-014-D4RA05347H-s001.pdf]
